# *O*-GlcNAcylation controls pro-fibrotic transcriptional regulatory signaling in myofibroblasts

**DOI:** 10.1038/s41419-024-06773-9

**Published:** 2024-06-03

**Authors:** Ninon Very, Clémence Boulet, Céline Gheeraert, Alexandre Berthier, Manuel Johanns, Mohamed Bou Saleh, Loïc Guille, Fabrice Bray, Jean-Marc Strub, Marie Bobowski-Gerard, Francesco P. Zummo, Emmanuelle Vallez, Olivier Molendi-Coste, Eloise Woitrain, Sarah Cianférani, David Montaigne, Line Carolle Ntandja-Wandji, Laurent Dubuquoy, Julie Dubois-Chevalier, Bart Staels, Philippe Lefebvre, Jérôme Eeckhoute

**Affiliations:** 1 Univ. Lille, Inserm, CHU Lille, Institut Pasteur de Lille, U1011-EGID, Lille, France; 2 Univ. Lille, Inserm, CHU Lille, U1286 - INFINITE – Institute for Translational Research in Inflammation, Lille, France; 3 Miniaturization for Synthesis, Analysis & Proteomics, UAR 3290, CNRS, University of Lille, Villeneuve d’Ascq Cedex, France; 4 Laboratoire de Spectrométrie de Masse BioOrganique, CNRS UMR7178, Univ. Strasbourg, IPHC, Infrastructure Nationale de Protéomique ProFI - FR2048, Strasbourg, France

**Keywords:** Glycosylation, Liver fibrosis, Transcriptomics

## Abstract

Tissue injury causes activation of mesenchymal lineage cells into wound-repairing myofibroblasts (MFs), whose uncontrolled activity ultimately leads to fibrosis. Although this process is triggered by deep metabolic and transcriptional reprogramming, functional links between these two key events are not yet understood. Here, we report that the metabolic sensor post-translational modification *O*-linked β-D-N-acetylglucosaminylation (*O*-GlcNAcylation) is increased and required for myofibroblastic activation. Inhibition of protein *O*-GlcNAcylation impairs archetypal myofibloblast cellular activities including extracellular matrix gene expression and collagen secretion/deposition as defined in vitro and using ex vivo and in vivo murine liver injury models. Mechanistically, a multi-omics approach combining proteomic, epigenomic, and transcriptomic data mining revealed that *O-*GlcNAcylation controls the MF transcriptional program by targeting the transcription factors Basonuclin 2 (BNC2) and TEA domain transcription factor 4 (TEAD4) together with the Yes-associated protein 1 (YAP1) co-activator. Indeed, inhibition of protein *O*-GlcNAcylation impedes their stability leading to decreased functionality of the BNC2/TEAD4/YAP1 complex towards promoting activation of the MF transcriptional regulatory landscape. We found that this involves *O*-GlcNAcylation of BNC2 at Thr^455^ and Ser^490^ and of TEAD4 at Ser^69^ and Ser^99^. Altogether, this study unravels protein *O-*GlcNAcylation as a key determinant of myofibroblastic activation and identifies its inhibition as an avenue to intervene with fibrogenic processes.

## Introduction

Myofibroblasts (MFs) are cornerstone cells in the processes of wound healing and tissue repair after injury. MFs emerge in injured tissues through a process called myofibroblastic activation where local “quiescent” fibroblasts or mesenchymal-like cells change phenotype to acquire the ability to produce and organize collagen/extracellular matrix (ECM) into scar tissue [1]. Unrestrained activity of MFs triggers fibrosis, a pathological condition characterized by the accumulation of excessive and structurally abnormal (ECM), which can affect virtually any organ and is detrimental to their functions [1, 2]. While fibrotic-related diseases, which could account for as much as 45% of all-cause mortality rates in developed countries, represent a major burden to human health, only few therapeutic strategies are currently available [3, 4]. Therefore, in a context where the medical need for effective anti-fibrotic therapies is high, a better knowledge of the molecular mechanisms involved in fibrosis development is urgently needed.

While responses to injury operates in an organ- and condition-specific manner, myofibroblastic activation involves conserved core cellular and molecular pathways [2]. Indeed, we and others have contributed to define that myofibroblastic activation is underlain by a deep transcriptional reprogramming [5, 6], which is triggered by canonical signals including the Transforming growth factor (TGF)-β and mechanotransducer Hippo/Yes-associated protein 1 (YAP1) signaling pathways [5, 7]. These pathways converge onto transcription factors (TFs), which establish the myofibroblastic transcriptional program including TFs of the Small mothers against decapentaplegic (SMAD) [8, 9], SNAI [10, 11], TWIST [12, 13], and TEA domain (TEAD) [14–16] families as well as Basonuclin 2 (BNC2), which we recently identified as a myofibroblast identity TF [6].

Myofibroblastic activation also involves deep metabolic reprogramming which allows to produce sufficient energy to support cell proliferation and biosynthetic processes [17, 18]. Key changes in MF metabolism consists in increased glucose and glutamine uptake together with a concomitant shift towards aerobic glycolysis (also known as the Warburg effect) and glutaminolysis [19–21]. These phenomena were first described in cancer cells where they are accompanied by a rise in activity of the hexosamine biosynthetic pathway (HBP) and downstream protein *O*-linked β-d-*N*-acetylglucosaminylation (*O*-GlcNAcylation) [22, 23]. Indeed, *O*-GlcNAcylation is a ubiquitous and dynamic process tightly regulated by the intracellular concentrations of the UDP-GlcNAc nucleotide-sugar donor, which is synthesized through HBP at the crossroad of glucose and glutamine metabolisms [24, 25]. UDP-GlcNAc is used by the ubiquitous enzyme *O*-GlcNAc transferase (OGT) to transfer GlcNAc monosaccharides to serine and threonine residues of intracellular proteins. This post-translational modification (PTM) regulates target protein interactions, stability, subcellular localization, enzymatic or transcriptional regulatory activities [22, 26]. In this context, we hypothesized that myofibroblastic activation could rely upon increased protein *O*-GlcNAcylation. To date, only sparse and conflicting reports with regard to the role and mechanisms of action of protein *O*-GlcNAcylation in MFs have been published [27–29]. Here, we have used liver MFs in the name of hepatic stellate cells (HSCs) as our main model to thoroughly assess the role of protein *O*-GlcNAcylation in MFs.

## Results

### *O*-GlcNAcylation is increased during myofibroblastic activation

**Fig. 1:**
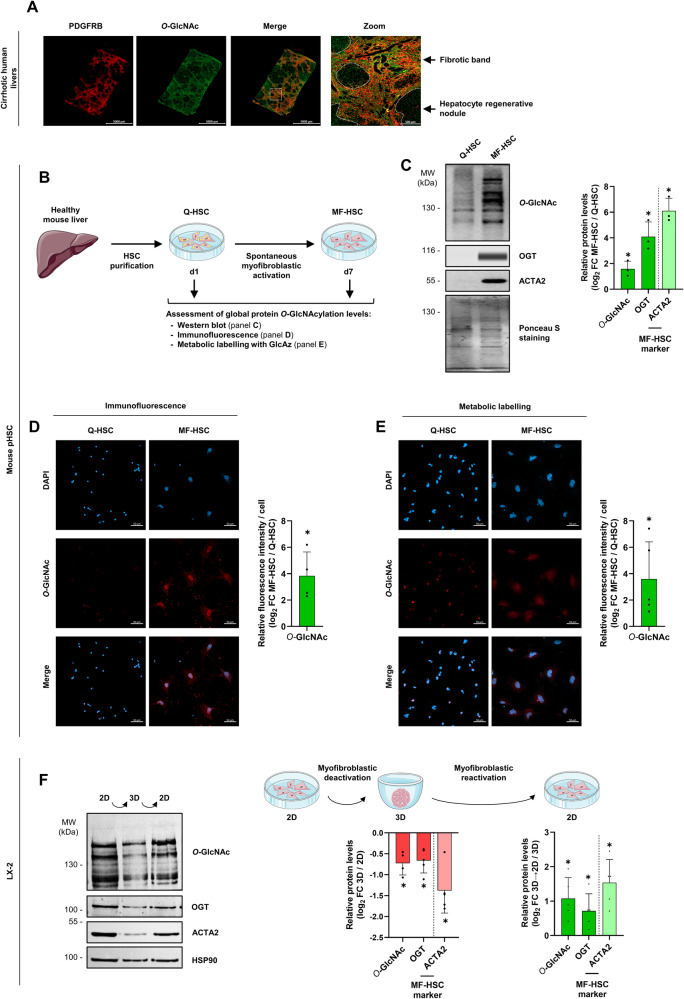
*O*-GlcNAcylation is increased during myofibroblastic HSC activation. **A** Representative images from co-immunostaining for the fibroblast marker PDGFRB (red) and *O*-GlcNAcylated proteins (green) in paraffin-embedded human liver sections from patients with alcohol-related cirrhosis (*n* = 10 biologically independent samples). White squares indicate regions for which a zoom image is provided on the right. Scale bars=5,000 µm for images showing entire sections and 500 µm for zoom image. Data obtained with livers from additional donors are shown in Supplementary Fig. 1A. **B** Graphical representation of experimental set-ups used in panels **C**–**E** to analyze *O*-GlcNAcylation in purified quiescent (Q-HSCs at 1 day (d) of culture) and spontaneously in vitro activated myofibroblastic HSCs (MF-HSCs at 7 d of culture). **C** Western blot and simple western immunoassays showing *O*-GlcNAcylation, OGT and ACTA2 protein levels in Q-HSCs and MF-HSCs. Ponceau S staining was used as protein loading control. The presented images are representative of 3 biologically independent experiments. MW, molecular weight markers. Log_2_ fold changes (log_2_ FC) between MF-HSCs and Q-HSCs are shown. **D** Immunofluorescence staining of protein *O*-GlcNAcylation with anti-*O*-GlcNAc antibody of Q-HSCs and MF-HSCs (shown images are representative of 4 biologically independent experiments). Scale bars=50 µm. Log_2_ FC between MF-HSCs and Q-HSCs is shown. **E** Immunofluorescence staining of protein *O*-GlcNAcylation in Q-HSCs and MF-HSCs revealed by metabolic labeling with GlcAz for 24 h followed by click chemistry with an alkyne-containing dye (shown images are representative of 5 biologically independent experiments). Scale bars = 50 µm. Log_2_ FC between MF-HSCs and Q-HSCs is shown. **F** Western blot assays showing *O*-GlcNAcylation, OGT and ACTA2 protein levels in LX-2 cells cultured in regular 2D conditions or grown as spheroids in 3D conditions for 72 h. A condition where spheroids were digested and cells put back into regular 2D culture plates (3D→2D) for 72 h is also shown. HSP90 was used as protein loading control. The presented images are representative of 5 biologically independent experiments. MW, molecular weight markers. Log_2_ FC between 3D and 2D cells (left panel), and between 3D→2D and 3D (right panel) are shown on the right. For all panels, the bar graphs show means + standard deviation (SD) together with individual biological replicates. Two-sided one-sample t-test with Benjamini–Hochberg correction for multiple testing was used to determine if the mean log_2_ FC was statistically different from 0.

As a first assessment of the potential connection between protein *O*-GlcNAcylation and myofibroblastic activation, we monitored protein *O*-GlcNAcylation in liver slices from patients with cirrhosis, i.e. the end stage of liver fibrosis (*n* = 10). While, as expected, *O*-GlcNAcylation was detected throughout the entire liver sections, fibrotic bands, where MFs were identified through co-immunostaining for Platelet-derived growth factor receptor beta (PDGFRB also known as PDGFR-β), displayed much more intense signal for protein *O*-GlcNAcylation when compared to hepatocyte regenerative nodule areas (Fig. 1A and Supplementary Fig. 1A). This observation led us to further assess protein *O*-GlcNAcylation levels upon myofibroblastic activation of HSCs, which are the main contributors to liver scarring [30, 31]. With this aim, we used purified primary murine HSCs (pHSCs) obtained from healthy livers, hereafter denoted as “quiescent” HSC (Q-HSC), which undergo spontaneous activation into myofibroblastic HSCs (MF-HSCs) upon seeding onto regular plastic culture plates [32] (Fig. 1B). This model allows to recapitulate the aforementioned concomitant metabolic and transcriptomic reprogramming [33, 34], which drive establishment of canonical myofibroblastic functionalities [35–37]. Using western blot assays, we observed a rise in global protein *O*-GlcNAcylation levels in MF-HSCs compared to Q-HSCs together with a concomitant increase in levels of OGT and the archetypal MF-HSC marker Actin alpha 2, smooth muscle (ACTA2 also known as α-SMA), used as a positive control (Fig. 1B, C). Similar conclusions were drawn when global protein *O*-GlcNAcylation was assessed using IF labeling with the anti-*O*-GlcNAc antibody (Fig. 1B, D) or using labeling of intracellular *O*-GlcNAcylated proteins by adding GlcAz, an azido-modified glucosamine precursor for OGT’s nucleotide sugar donor UDP-GlcAz, to the culture medium for 24 hours followed by click reaction with an alkyne-containing dye (Fig. 1B, E). These experiments revealed that protein *O*-GlcNAcylation was primarily nuclear as indicated through comparison with DAPI staining (Fig. 1D, E). In order to further define a link between *O*-GlcNAcylation levels and HSC activation, we next leveraged the ability to modulate the phenotype of the human MF-HSC cell-line LX-2 by growing cells in 3 dimensions (3D) as non-embedded spheroids, which alleviates environmental stiffness-induced activation [6, 38] (Supplementary Fig. 1B). We found that, along with the MF-HSC marker ACTA2, protein *O*-GlcNAcylation and OGT levels were lower in LX-2 cells grown in 3D when compared to conventional 2D culture conditions (Fig. 1F). All these changes were reversed when spheroids were dissociated and cells seeded back into regular 2D cell culture plates (Fig. 1F and Supplementary Fig. 1B). We also found that treatment with the pro-fibrotic cytokine TGF-β further raises global protein *O*-GlcNAcylation levels in LX-2 cells (Supplementary Fig. 1C).

Together, these results indicate that HSC myofibroblastic activation involves an increase in protein *O*-GlcNAcylation, which led us to consider the role exerted by this modification in MF-HSCs.

### *O-*GlcNAcylation is required for collagen encoding gene expression by MF-HSCs in vitro and in mouse models of liver injury

**Fig. 2:**
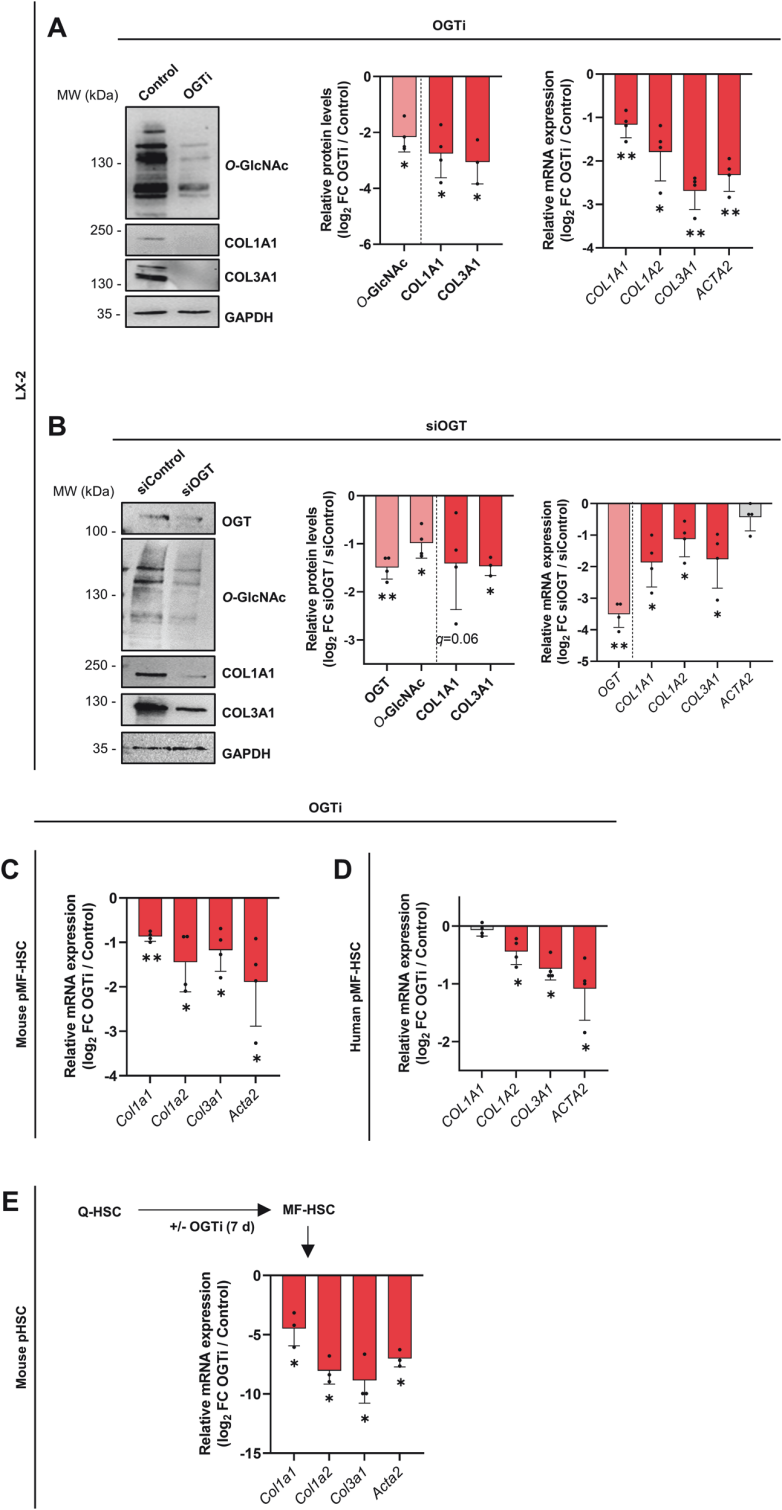
*O-*GlcNAcylation is required for expression of fibrogenic collagen encoding genes in isolated MF-HSCs. **A** LX-2 cells were treated or not with 50 µM of the OGT inhibitor OSMI-1 (referred to as OGTi) for 24 h. Western blot assays (left panel) and their quantifications (middle panel) of *O*-GlcNAcylation, COL1A1 and COL3A1 protein levels are shown. GAPDH was used as protein loading control. The presented images are representative of at least three biologically independent experiments. MW, molecular weight markers. RT-qPCR data showing indicated collagen encoding gene expression are also displayed (right panel, *n* = 4 biologically independent experiments). Log_2_ FC between OGTi and control (non-treated) conditions are shown. **B** LX-2 cells were transfected with 20 nM siControl or siOGT for 72 h. Western blot assays (left panel) and their quantifications (middle panel) of OGT, *O*-GlcNAcylation, COL1A1 and COL3A1 protein levels are shown. GAPDH was used as protein loading control. The presented images are representative of at least three biologically independent experiments. MW, molecular weight markers. RT-qPCR data showing gene expression (right panel, *n* = 4 biologically independent experiments). Log_2_ fold changes (log_2_ FC) between siOGT and siControl conditions are shown. **C** Mouse pMF-HSCs (7 d of culture) were treated or not with 50 µM OGTi for 24 h (*n* = 4 biologically independent experiments). RT-qPCR data show indicated collagen encoding gene expression. Log_2_ FC between OGTi and control (non-treated) conditions are shown. **D** Human pMF-HSCs were treated or not with 50 µM OGTi for 24 h. RT-qPCR data show indicated collagen encoding gene expression. Log_2_ FC between OGTi and control (non treated) conditions are shown. **E** Mouse pQ-HSCs (1 d of culture) were treated or not with 50 µM OGTi for 7 d (*n* = 3 biologically independent experiments). RT-qPCR data show indicated collagen encoding gene expression. Log_2_ FC between OGTi and control (non-treated) conditions are shown. For all panels, the bar graphs show means + SD together with individual biological replicates. Two-sided one-sample t-test with Benjamini–Hochberg correction for multiple testing was used to determine if the mean log_2_ FC was statistically different from 0.

MF-HSCs promote ECM buildup notably through deposition of fibrillary type I and III collagens [39]. Further pointing to a role for protein *O-*GlcNAcylation in MF-HSCs, we first observed in LX-2 cells that expression of collagen encoding genes, including *COL1A1*, *COL1A2* and *COL3A1*, are dependent upon nutrient sources, like glucose and glucosamine, which fuel the HBP and protein *O*-GlcNAcylation pathways (Supplementary Fig. 2A). As a more direct approach, we next treated MF-HSCs with the pharmacological OGT inhibitor OSMI-1 (referred to as OGTi hereafter). As expected, OGTi reduced overall protein *O*-GlcNAcylation in LX-2 cells (Fig. 2A). Treatment with OGTi also significantly decreased expression of *COL1A1*, *COL1A2*, and *COL3A1* (Fig. 2A). The decrease in COL1A1 and COL3A1 was confirmed at the protein level by western blotting (Fig. 2A). These repressive effects of OGTi were dose-dependent (Supplementary Fig. 2B). Maximal collagen encoding gene repression was reached at 50 µM (Supplementary Fig. 2B), a concentration which did not trigger any significant cytotoxicity and was further used throughout this study (Supplementary Fig. 2C, D). The specific role of OGT in controlling collagen encoding gene expression was validated by treating LX-2 cells with ST045849, another OGT inhibitor, which also led to a decrease in their expression (Supplementary Fig. 2E). Moreover, similar results were obtained upon siRNA-mediated genetic knockdown of OGT (siOGT) (Fig. 2B). OGT-dependent collagen encoding gene expression extended to both primary murine and human MF-HSCs (pMF-HSCs), as defined by treating these cells with OGTi (Fig. 2C, D). The effect was even more pronounced when primary mouse Q-HSCs were treated with OGTi throughout the entire in vitro activation period (7 days) (Fig. 2E). A lower ability to reduce 3-(4,5-dimethylthiazol-2-yl)-2,5-diphenyltetrazolium bromide (MTT) pointed to decreased metabolic activity in these treated cells suggesting that they remained in a quiescent-like state (Supplementary Fig. 2F, G).

**Fig. 3:**
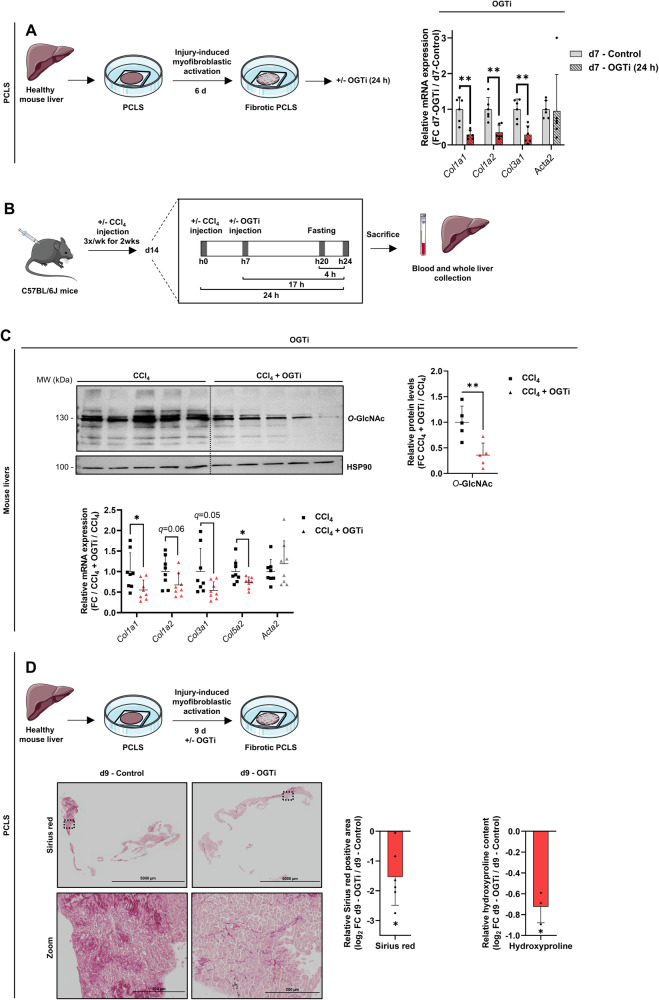
*O-*GlcNAcylation is required for MF-HSC expression of fibrogenic collagen encoding genes in ex vivo and in vivo mouse models of liver injury. **A** Mouse PCLS were cultured for 6 d to induce fibrosis and then subjected or not to OGTi (50 µM OSMI-1) for 24 h as depicted in the schematic. Bar graph shows RT-qPCR data monitoring changes in expression of indicated collagen encoding genes (*n* = 6 PCLS from independent mice). Fold changes (FC) relative to d7 non-treated (d7 – Control; arbitrarily set to 1) are shown. Batch effect was removed by setting the mean of d7 non-treated conditions for each series of experiments, i.e. performed at different times, to 1. One-tailed Mann-Whitney *U* test with Benjamini-Hochberg correction for multiple testing was used. **B** Graphical representation of the experimental set-up used to assess the effect of OGTi on CCl_4_-induced HSC activation in vivo. C57BL/6J mice were injected with olive oil (control mice) or 0.5 mL/kg CCl_4_ three times a wk for 2 wks. 7 h after the last injection, CCl_4_ mice were injected (CCl_4_ + OGTi mice) or not (CCl_4_ mice) with 1 mg OGTi for 17 h. **C** Western blot assays and their quantifications showing *O*-GlcNAcylation levels in livers of CCl_4_ (*n* = 5 mice) and CCl_4_ + OGTi mice (*n* = 5 mice; top). HSP90 was used as protein loading control. Bar graph at the bottom shows RT-qPCR data monitoring changes in collagen encoding gene expression in livers of mice treated with CCl_4_ (*n* = 8 mice) *versus* those treated with CCl_4_ + OGTi (*n* = 8 mice). FC between mice of the CCl_4_ + OGTi and CCl_4_ group (arbitrarily set to 1) are shown. One-tailed Mann-Whitney *U* test with Benjamini-Hochberg correction for multiple testing was used to assess the statistical significance of differences between the CCl_4_ + OGTi and CCl_4_ groups. **D** Mouse PCLS were cultured for 9 d to induce fibrogenic collagen deposition in presence or not of 50 µM OGTi as depicted in the schematic (top panel). Sirius red staining was performed to monitor collagen deposition (shown images are representative of *n* = 6 PCLS from independent mice, bottom left panel). Scale bars = 5000 µm for images showing entire sections and 200 µm for zoom image. Log_2_ FC between OGTi and control conditions are shown for relative Sirius red positive areas (bottom middle panel) and hydroxyproline content (*n* = 3 PCLS from independent mice, bottom right panel). Two-sided one-sample t-test was used to determine if the mean log_2_ FC was statistically different from 0. For all panels, the graphs show means + SD together with individual biological replicates or mice.

To further assess the ability of OGT inhibition to impair primary MF-HSC activities, we next examined the effect of OGT inhibition in mouse precision-cut liver slices (PCLS). Preparation of PCLS involves slicing-induced damage and cell death, which trigger injury-induced HSC activation in the absence of immune cell infiltration [40, 41]. In line, after 7 days of culture, we observed a significant increase in collagen encoding gene expression in PCLS concomitant with a decrease in expression of functional hepatocyte markers (Supplementary Fig. 3A, B). Interestingly, induction of collagen encoding gene expression was significantly reversed when PCLS were further incubated with OGTi for 24 hours (Fig. 3A and Supplementary Fig. 3B). We next extended our analyses to in vivo HSC myofibroblastic activation by monitoring the effect of OGTi treatment in the carbon tetrachloride (CCl_4_) model of liver injury. Indeed, injection of mice with hepatotoxic CCl_4_ is well-documented to trigger a MF-HSC-dependent wound-healing response [28]. Moreover, relevance of the CCl_4_ model to study the transcriptional reprogramming associated with myofibroblastic activation of HSCs was previously established in studies which defined that the transcriptional program underlying fibrogenic HSC activation in this model was remarkably similar to that observed across other disease models including models of metabolic dysfunction-associated steatohepatitis (MASH) [36, 37]. As expected, CCl_4_ administration triggered liver injury as revealed by significantly increased plasma Alanine aminotransferase (ALT) and Aspartate aminotransferase (AST) activities (Supplementary Fig. 3C), decreased gene expression of functional hepatocyte markers (Supplementary Fig. 3D) and elevated expression of MF-HSC markers (Supplementary Fig. 3E). As our in vitro and ex vivo data had shown that acute OGTi treatment was sufficient to trigger a detectable effect on MF-HSCs using collagen encoding gene expression as a readout, CCl_4_ exposed mice were subjected to a single OGTi injection and livers were collected 17 hours later (Fig. 3C). Importantly, this acute exposure to OGTi allowed to avoid any further effect on the expression of functional hepatocyte markers and crucially on *Cytochrome P450 2E1* (*Cyp2e1*), the enzyme responsible for metabolizing CCl_4_ into the active trichloromethyl radical CCl_3_^-^ in hepatocytes [42] (Supplementary Fig. 3C, D). Importantly, and reminiscent of our findings in PCLS, inhibition of protein *O*-GlcNAcylation with OGTi led to reduced expression of the collagen encoding genes *Col1a1*, *Col1a2*, *Col3a1*, and *Col5a2* (Fig. 3C). Considering that collagen encoding gene expression in livers of CCl_4_ treated mice unambiguously primarily originates from MF-HSCs (Supplementary Fig. 3F), these data indicate that the in vivo effect of OGTi is to be attributed to these cells.

Finally, in order to define whether OGTi-mediated inhibition of HSC myofibroblastic activation translated into inhibition of fibrogenic collagen deposition, we exposed PCLS to OGTi during 9 days. At this time, non-treated PCLS show substantial collagen deposition (Supplementary Fig. 3G). OGTi treatment was found to significantly dampen this process, along with the expected decrease in collagen encoding gene expression (Supplementary Fig. 3H, I), as judged using both Sirius red staining and hydroxyproline content measurement (Fig. 3D).

Altogether, these data demonstrate that *O*-GlcNAcylation is required for fibrogenic collagen encoding gene expression by MF-HSCs both in isolated cells and in models of liver injuries.

### *O*-GlcNAcylation exerts a global control over the myofibroblastic transcriptomic program and MF-HSC functionalities

**Fig. 4:**
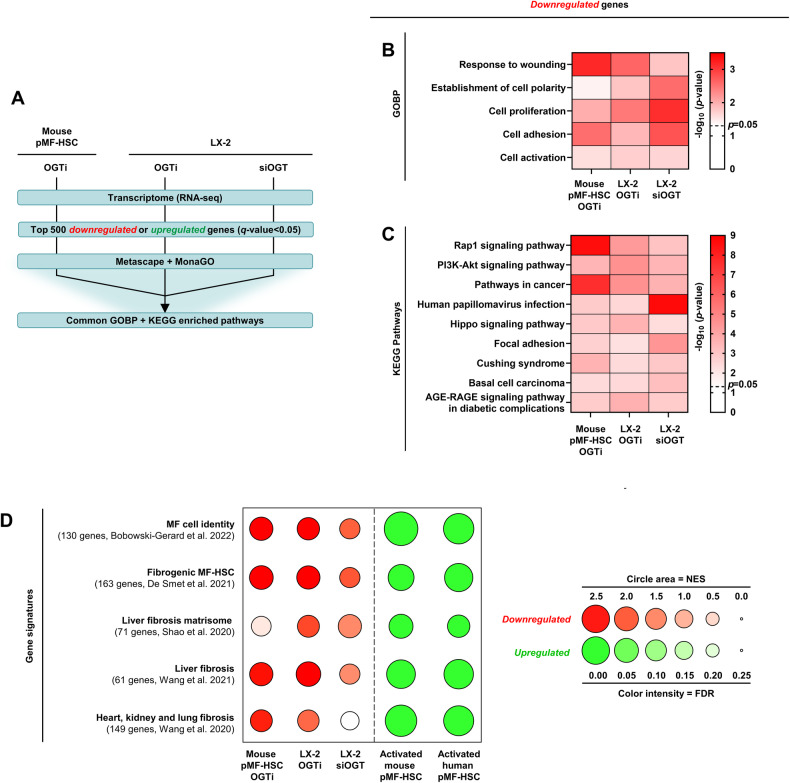
*O*-GlcNAcylation controls the MF transcriptomic program. **A** Outline of the strategy used to define the role of protein *O*-GlcNAcylation in the control of the MF-HSC transcriptome. RNA-seq was performed on mouse pMF-HSCs (7 d of culture; *n* = 4 biologically independent experiments) and LX-2 cells (*n* = 4 biologically independent experiments) treated or not with OGTi (50 µM OSMI-1) for 24 h as well as LX-2 cells transfected or not with 20 nM siOGT for 72 h (*n* = 3 biologically independent experiments). For each one of the three models and spontaneous in vitro activation of murine [49] and human [50] pHSCs, pathway enrichment analyses (**B**, **C**) and GSEA (**D**) and were performed. **B**, **C** GO Biological Process (GOBP; **B**) and KEGG Pathway (**C**) enrichment analyses were performed using the top 500 downregulated genes upon treatment with OGTi or siOGT (see “Materials and methods” section) using Metascape. Enriched GOBP terms were clustered according to the Resnik similarity using MonaGO. Heatmaps show pathways commonly enriched in RNA-seq data from all three models. **D** Dot plot depicting the results of GSEA performed using MF cell identity [6], fibrogenic MF-HSCs [45], liver fibrosis matrisome [46], liver fibrosis [47] and heart, kidney and lung fibrosis [48] gene sets. Circle areas are proportional to the normalized enrichment score (NES), while color intensity indicates the false discovery rate (FDR). Red color indicates a negative NES (gene signature biased toward downregulated genes upon treatment with OGTi or siOGT), green color indicates a positive NES (gene signature biased toward upregulated genes upon in vitro activation of pHSCs) and white color indicates a lack of significance.

To more comprehensively define the impact of *O*-GlcNAcylation inhibition in MF-HSCs, we conducted transcriptomic analyses in mouse pMF-HSCs and LX-2 cells treated with OGTi or siOGT (Fig. 4A). Enrichment analyses for gene ontology terms related to biological pathways (GOBP) and KEGG pathways were performed using the 500 most significantly down- or upregulated genes in all 3 models (Fig. 4A and Supplementary Tables 1-2). Upregulated genes only showed conserved enrichment for genes that may relate to growth arrest (Supplementary Fig. 4A) and did not reveal induction of TRs previously described to deactivate MFs such as *Peroxisome proliferator-activated receptor gamma* (*PPARG*) [14, 43]. However, downregulated genes were characterized by conserved enrichment for pathways/terms related to intrinsic and canonical MF-HSC functions including response to wounding, establishment of cell polarity, cell proliferation, adhesion, and activation (Fig. 4B). In line, pathways controlling MF-HSC fibrogenic activities, such as the Hippo/YAP1 signaling pathway, were also retrieved in those analyses (Fig. 4C) [44]. Furthermore, gene set enrichment analysis (GSEA) indicated that genes characterizing the pan-myofibroblast identity, which we previously defined in Bobowski-Gerard et al. [6], were significantly biased towards downregulation upon OGT inhibition in all 3 experimental models (Fig. 4D, Supplementary Fig. 4B, and Supplementary Table 3). Consistent with *O*-GlcNAcylation inhibition broadly interfering with the myofibroblastic transcriptional program, GSEA also revealed that fibrotic gene signatures from the liver [45–47] or other organs (heart, kidney, and lung) [48] were equally enriched among downregulated genes (Fig. 4D, Supplementary Fig. 4B, and Supplementary Table 3). On the contrary, and as expected, these gene sets were enriched among upregulated genes during activation of murine [49] and human [50] pHSC (Fig. 4D and Supplementary 4B). In line, MF obtained from primary human cardiac fibroblasts and the lung LL29 MF cell line were also sensitive to OGTi treatment as defined through reduced expression of collagen-encoding genes (Supplementary Fig. 4C, D).

**Fig. 5:**
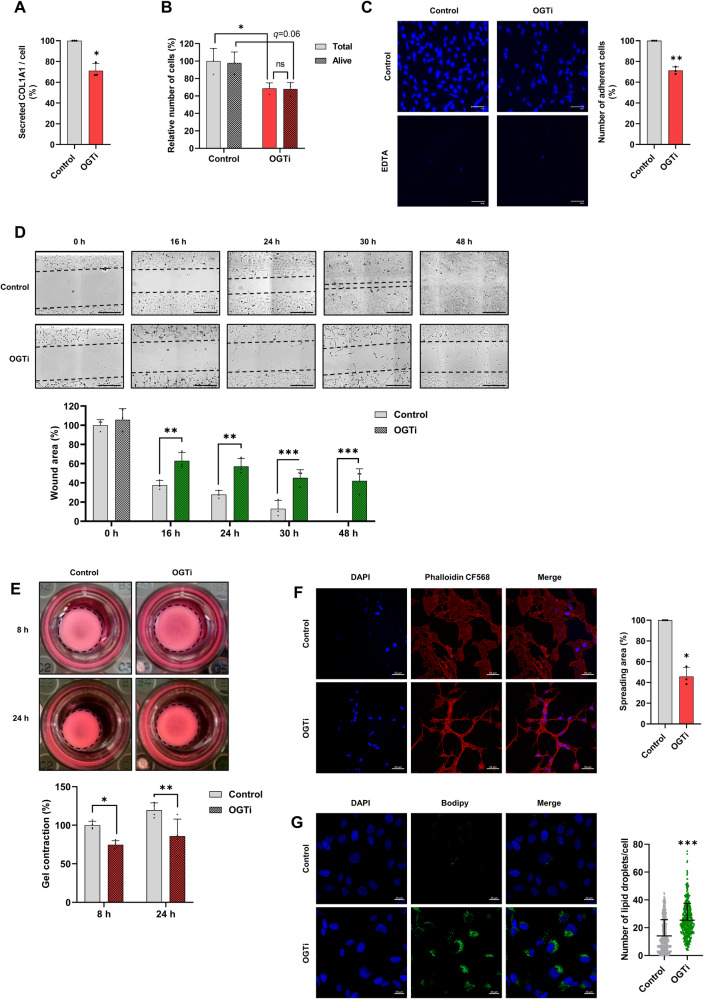
*O*-GlcNAcylation controls myofibroblastic activities of MF-HSCs. **A** LX-2 cells were treated or not with OGTi (50 µM OSMI-1) for 24 h. Secreted COL1A1 was measured in the supernatant by ELISA and is reported relative to the number of cells evaluated by TC20 Automated Cell Counter (*n* = 3 biologically independent experiments). Secreted COL1A1 per cell is shown relative to that in the control (non-treated) condition arbitrarily set to 100. Two-sided one-sample t-test was used to determine if the mean log_2_ fold changes (log_2_ FC) between OGTi and control conditions were statistically different from 0. **B** LX-2 cells were treated as in **A** and number of total and alive (trypan blue-negative) cells were counted (*n* = 3 biologically independent experiments). Number of cells is shown relative to that in the control (total non-treated) condition arbitrarily set to 100. Two-way ANOVA with Sidak multiple comparison post-hoc test was used. ns, not significant. **C** LX-2 cells were treated as in **A** and Hoescht 33258 staining of adherent cells was performed to assess binding to collagen-coated matrix (shown images are representative of 3 biologically independent experiments). EDTA treatment was used a control of signal specificity. Scale bars = 50 µm. Number of adherent cells is shown relative to that in the control (non-treated) condition arbitrarily set to 100. Two-sided one-sample t-test was used to determine if the mean log_2_ FC between OGTi and control conditions was statistically different from 0. **D** LX-2 cells were grown to confluence, treated or not with 50 µM OGTi upon insert removal (shown images are representative of three biologically independent experiments) and wound area was measured 0, 16, 24, 30, and 48 h later. Scale bars = 300 µm. Wound area width is shown relative to that at of the control (non-treated) condition at 0 h arbitrarily set to 100. Two-way ANOVA with Sidak multiple comparison post-hoc test was used to assess the statistical significance of differences between the OGTi and control conditions. **E**. LX-2 cells were grown into a collagen matrix gel and treated or not with 50 µM OGTi for 16 h. Collagen gel contraction was defined by measuring the gel diameter (see “Materials and methods” section) 8 h and 24 h after release from the well (shown images are representative of 4 biologically independent experiments). Gel contraction is shown relative to that in the control (non-treated at 8 h) condition arbitrarily set to 100. Two-way ANOVA with Sidak multiple comparison post-hoc test was used to assess the statistical significance of differences between the OGTi and control conditions. **F** LX-2 cells were treated as in **A** and fluorescence staining of F-actin stress fibers was performed with CF® 568-conjugated phalloidin (shown images are representative of three biologically independent experiments). Scale bars=50 µm. Spreading area is shown relative to that in the control condition arbitrarily set to 100. Two-sided one-sample t-test was used to determine if the mean log_2_ FC between OGTi and control conditions were statistically different from 0. **G** LX-2 cells were treated as in **A** and fluorescence staining of lipid droplets was performed with BODIPY (shown images are representative of 3 biologically independent experiments). Scale bars = 20 µm. Number of lipid droplets per cell is shown (*n* = 150 cells per condition were counted in each replicate, i.e., *n* = 450 cells per condition were counted in total). Unpaired non-parametric Mann–Whitney test was used to assess if the difference between OGTi- and non-treated cells was statistically significant. For all panels, the graphs show means + SD together with individual biological replicates or cells.

Since the transcriptomic data pointed to alterations in several of the archetypal myofibroblastic functionalities, we subjected OGTi-treated LX-2 cells to a series of cellular functional assays. Our results revealed that OGTi treatment significantly reduced COL1A1 secretion (Fig. 5A), triggered a decrease in total cell number without any effect on cell viability pointing to reduced cell proliferation (Fig. 5B), impaired adhesion to collagen matrix (Fig. 5C), in vitro wound-healing capacity (Fig. 5D) and collagen gel contraction (Fig. 5E). This was linked to an altered organization and spreading of F-actin stress fibers (Fig. 5F) as well as increased lipid droplets content, a feature of quiescent HSCs [51] (Fig. 5G). We confirmed that ST045849 produced similar effects (Supplementary Fig. 5).

Altogether, there results demonstrated that *O*-GlcNAcylation controls the myofibroblastic transcriptomic program and associated functionalities in MF-HSCs.

### *O*-GlcNAcylation controls the MF-HSC chromatin regulatory landscape by promoting activities of a BNC2/TEAD4/YAP1 transcriptional regulatory complex

**Fig. 6:**
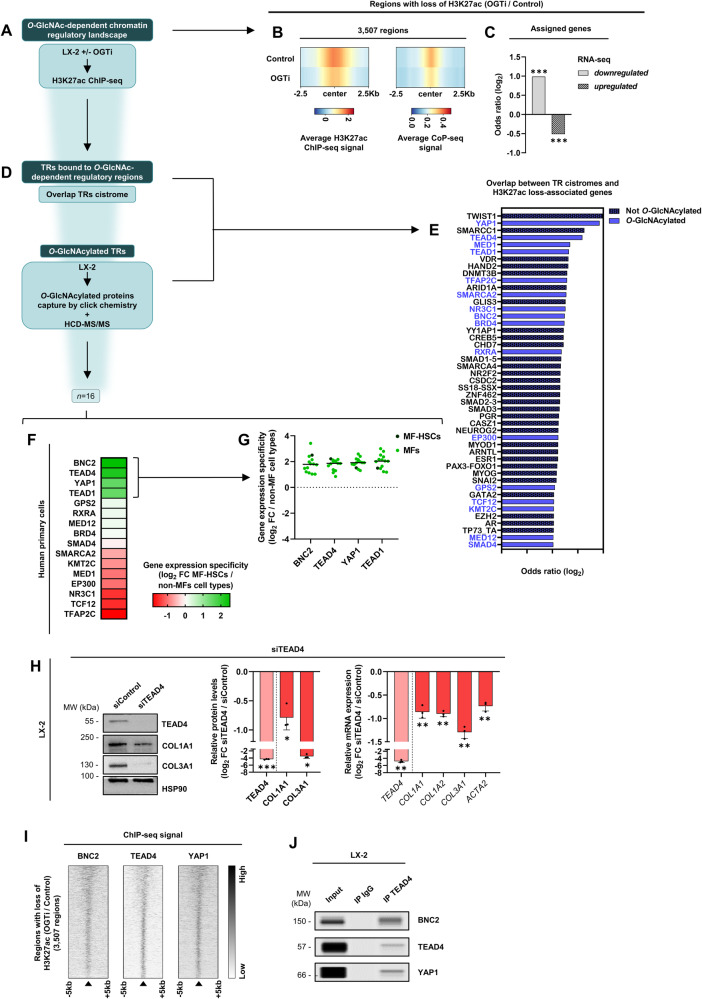
Multi-omic analyses reveal a role for *O*-GlcNAcylation in controlling the myofibroblastic transcriptional regulatory landscape. **A** Identification of transcriptional regulatory regions decommissioned upon treatment with OGT inhibitor OSMI-1 (referred to as OGTi) in LX-2 cells using mining of H3K27ac ChIP-seq data. **B** Characterization of *O*-GlcNAcylation-dependent transcriptional regulatory regions. Average H3K27ac ChIP-seq (*n* = 4 biologically independent experiments) and CoP-seq signals from LX-2 cells treated or not with 50 µM OGTi for 24 h. Heatmaps show the signals in ±2.5 kb windows around the center of regions identified as displaying a significant loss of H3K27ac in OGTi-treated cells (*n* = 3,507 regions). **C** Association between transcriptional regulatory regions losing H3K27ac (from **B**) and deregulated genes (RNA-seq from Fig. 4) in OGTi-treated LX-2 cells displayed as log_2_ Odds ratio. See “Materials and methods” section for the procedure used to assign H3K27ac regions to genes. Two-sided Fisher’s exact test was used to assess the statistical significance of the biased association with down- or upregulated genes. **D** Identification of TRs bound to *O*-GlcNAc-dependent regulatory regions (i.e. comparison of a database of TR cistromes with regions showing significant loss of H3K27ac in OGTi-treated cells) was performed along with identification of *O*-GlcNAcylated TRs in LX-2 cells using a click chemistry-based approach coupled to mass spectrometry as described in the “Materials and methods” section (*n* = 3 biologically independent experiments). **E**. TRs whose cistrome significantly overlaps with regions losing H3K27ac upon OGTi treatment of LX-2 cells were retrieved as detailed in the “Materials and methods” section and are reported here. Those identified in the LX-2 *O*-GlcNAcome were further highlighted in blue. **F** The 16 *O*-GlcNAcylated TRs (from **E**) were ranked according to their specificity of expression in MF-HSCs defined as log_2_ fold changes (log_2_ FC) in MF-HSCs compared to average expression in 112 other human non-MFs primary cell types. **G**. Specific expression of BNC2, TEAD4, YAP1 and TEAD1 in MFs is shown using log_2_ FC in MF-HSCs or MFs (*n* = 13) compared to average expression in 112 other human non-MFs primary cell types. **H** LX-2 cells were transfected with 20 nM siTEAD4 or siControl and cells were harvested 72 h later. Western blot assays (left panel) and TEAD4, COL1A1 and COL3A1 protein levels quantifications (middle panel) are shown. HSP90 was used as protein loading control. The presented images are representative of 3 biologically independent experiments. MW, molecular weight markers. The right graph shows RT-qPCR data (*n* = 3 biologically independent experiments). Log_2_ FC between siTEAD4 and siControl conditions are shown. The bar graph shows means + SD together with individual biological replicates. Two-sided one-sample t-test with Benjamini-Hochberg correction for multiple testing was used to determine if the mean log_2_ fold changes (log_2_ FC) were statistically different from 0. **I**. Heatmaps show the average BNC2, TEAD4 (LX-2 cells) as well as YAP1 (IMR90 cells) ChIP-seq signals in ±5 kb windows around the center of regions identified as displaying a significant loss of H3K27ac in OGTi-treated cells (*n* = 3507 regions from **B**). **J** Nuclear extracts from LX-2 cells were subjected to immunoprecipitation with an antibody against TEAD4 (ab58310, Abcam). Input and immunoprecipitated materials were analyzed by simple western immunoassay using antibodies directed against BNC2, TEAD4, and YAP1. The presented data are representative of two biologically independent experiments.

In order to define mechanisms underlying the *O*-GlcNAcylation-mediated control of the MF-HSC transcriptomic program, we first assessed the effect of OGTi treatment on activity of transcriptional regulatory regions in LX-2 cells (Fig. 6A). With this aim, we monitored how OGTi modulates the levels of the activation mark histone H3 lysine 27 acetylation (H3K27ac) using chromatin immunoprecipitation (ChIP)-seq. We found that OGTi treatment resulted in loss of H3K27ac signal at 3 507 genomic regions, which showed concomitant loss in chromatin accessibility as determined using the column purified chromatin (CoP)-seq method, which enriches for genomic regions with accessible DNA through its binding to silica-gel membranes [52] (Fig. 6B and Supplementary Fig. 6A). As expected, genes associated with these inactive regulatory regions were enriched for downregulated genes in the transcriptomic data of OGTi-treated LX-2 cells (Fig. 6C and Supplementary Fig. 6B) and for pathways related to archetypal MF-HSC functions (Supplementary Fig. 6C and Supplementary Table 4). Comparing these OGTi-inactivated regulatory regions with human TR binding sites defined using ChIP-seq (*n* = 8112 curated cistromes essentially obtained from the ReMap2022 database [53]) identified 47 TRs with significant overlap (Fig. 6D, E and Table 5). Interestingly, 16 of these TRs were also recovered when *O*-GlcNAcylated TRs were identified from nuclear extracts of LX-2 cells using a click chemistry-based capture of *O*-GlcNAcylated proteins coupled with mass spectrometry (Fig. 6D, E and Supplementary Table 6). Among those, BNC2, TEAD1/4, and YAP1 stood out as being characterized by privileged expression in MF-HSCs (Fig. 6F) and more broadly in MFs (*n* = 13, Fig. 6G) when compared to the average expression in 112 other primary human non-MFs cell types. The YAP1 cofactor is required for myofibroblastic activation [16, 38, 54]. We recently defined that BNC2, a poorly studied TF, was involved in this process [6], which had also been posited to rely on TEAD TFs [16, 55]. In line with a role for TEAD TFs in MF-HSCs [14, 15], the TEAD recognition motif was found to be enriched in regions with OGTi-induced loss of H3K27ac in LX-2 cells (Supplementary Fig. 6D) and silencing of TEAD4 was sufficient to significantly reduced expression of fibrogenic genes both at the mRNA and protein levels (Fig. 6H). Moreover, we performed TEAD4 ChIP-seq in LX-2 cells (Supplementary Fig. 6E) and found that TEAD4 binding characterizes regions with OGTi-induced loss of H3K27ac together with BNC2 [6] and YAP1 [56] (Fig. 6I). Interestingly, co-immunoprecipitation assays performed using nuclear extracts of LX-2 cells showed that these TRs physically interact (Fig. 6J and Supplementary Fig. 6F) further indicating that they cooperate in MF-HSCs.

**Fig. 7:**
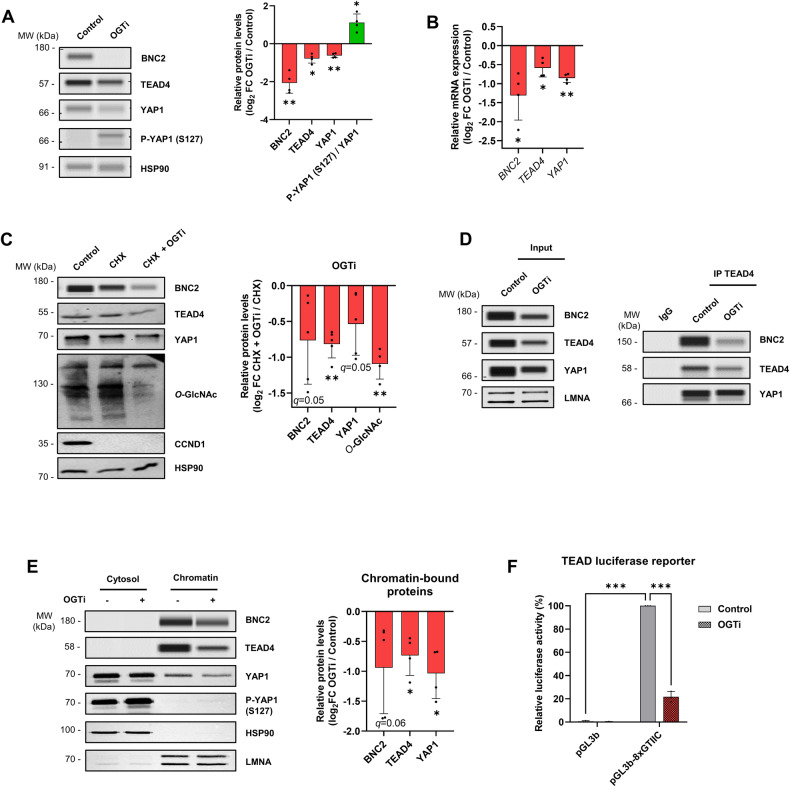
*O*-GlcNAcylation controls the myofibroblastic BNC2/TEAD4/YAP1 transcriptional regulatory complex. **A** LX-2 cells were treated or not with 50 µM of the OGT inhibitor OSMI-1 (referred to as OGTi) for 24 h. Western blot assays and their quantifications of BNC2, TEAD4, YAP1, and phospho-YAP1 (S127) protein levels are shown. HSP90 was used as protein loading control. The presented images are representative of 4 biologically independent experiments. MW, molecular weight markers. Log_2_ fold changes (log_2_ FC) between OGTi treatment and control conditions (right panel) are shown. Two-sided one-sample t-test with Benjamini–Hochberg correction for multiple testing was used to determine if the mean log_2_ FC between OGTi and control conditions was statistically different from 0. **B** LX-2 cells were treated as in **A**. RT-qPCR was used to monitor gene expression (*n* = 4 biologically independent experiments). Log_2_ FC between OGTi treatment and control conditions are shown. Two-sided one-sample t-test with Benjamini–Hochberg correction for multiple testing was used to determine if the mean log_2_ FC between OGTi and control conditions was statistically different from 0. **C**. LX-2 cells were pre-treated with 20 µg/mL cycloheximide (CHX) for 1 h and then treated with CHX combined or not with OGTi (50 µM OSMI-1) for an additional 24 h. Western blot and simple western immunoassays for BNC2, TEAD4, YAP1, CCND1, and protein *O*-GlcNAcylation levels together with quantifications of BNC2, TEAD4, YAP1, and *O*-GlcNAcylation levels are shown. HSP90 was used as protein loading control. The presented images are representative of at least 4 biologically independent experiments. MW, molecular weight markers. Log_2_ FC between CHX + OGTi and CHX conditions are shown. Two-sided one-sample t-test with Benjamini-Hochberg correction for multiple testing was used to determine if the mean log_2_ FC between CHX + OGTi and CHX conditions was statistically different from 0. **D** LX-2 cells were treated as in **A**. Nuclear extracts were subjected to immunoprecipitation with an antibody against TEAD4 (ab58310, Abcam). Input and immunoprecipitated materials were analyzed by western blot and simple western immunoassay using antibodies directed against BNC2, TEAD4, or YAP1. LMNA was used as protein loading control. The presented data are representative of two biologically independent experiments. **E** LX-2 cells were treated as in **A** and sub-cellular fractionation was performed to obtain cytosolic and chromatin fractions. Western blot and simple western immunoassays of BNC2, TEAD4, YAP1, and P-YAP1 (S127) levels and quantifications of chromatin-bound BNC2, TEAD4 and YAP1 are shown. HSP90 and LMNA were used as protein loading controls. The presented images are representative of at least 4 biologically independent experiments. MW molecular weight markers. Two-sided one-sample t-test with Benjamini–Hochberg correction for multiple testing was used to determine if the mean log_2_ FC between OGTi and control conditions was statistically different from 0. **F** LX-2 cells were transfected with a control luciferase reporter vector (pGL3-basic (pGL3b) promoter) or a TEAD-responsive luciferase reporter vector (pGL3b-8xGTIIC-luciferase plasmid) for 68 h and then treated or not with OGTi (50 µM OSMI-1) for an additional 24 h (*n* = 3 biologically independent experiments). Luciferase activities relative to that obtained with the pGL3b-8xGTIIC untreated (control) condition arbitrarily set to 100 are shown. Two-way ANOVA with Sidak multiple comparison post-hoc test was used. For all panels, the bar graphs show means + SD together with individual biological replicates.

*O*-GlcNAcylation of BNC2, TEAD4, and YAP1 was further verified using click chemistry-based capture of *O*-GlcNAcylated proteins followed by western blotting (Supplementary Fig. 7). To define how *O*-GlcNAcylation functionally controls the MF-HSC transcriptome through these transcriptional regulators, we first assessed whether this PTM modulates their protein levels. We observed that OGTi treatment of LX-2 cells triggers a decrease in BNC2, TEAD4, and YAP1 proteins (Fig. 7A). Regarding YAP1, reminiscent of previous findings in cancer cells [57, 58], this was in addition linked to an increase in phosphorylation of YAP1 at Ser^127^ (Fig. 7A), which induces its cytoplasmic sequestration and degradation. Since OGTi also triggered a decrease in *BNC2*, *TEAD4* and *YAP1* gene expression (Fig. 7B), we next directly assessed a role for *O*-GlcNAcylation on protein stability by applying OGTi to LX-2 cells pre-treated with the translation inhibitor cycloheximide (CHX) to prevent de novo protein synthesis. Western blotting for protein *O*-GlcNAcylation and Cyclin D1 (CCND1; a protein with short half-life) was used to control the effects of the OGTi and CHX treatments, respectively (Fig. 7C). These experiments indicated that the decrease in BNC2, TEAD4, and YAP1 levels involves OGTi-mediated reduced protein stability (Fig. 7C). As a consequence, OGTi treatment diminished the formation of the nuclear complex between TEAD4, BNC2, and YAP1 (Fig. 7D) and decreased their chromatin binding (Fig. 7E). In line, activity of TEAD-dependent transcriptional response elements in LX-2 cells was significantly dampened by OGTi (Fig. 7F). Since OGTi treatment did not alter TEAD1 protein levels or chromatin binding (Supplementary Fig. 8A, B), the observed impaired TEAD activity was more likely to be attributed to TEAD4.

**Fig. 8:**
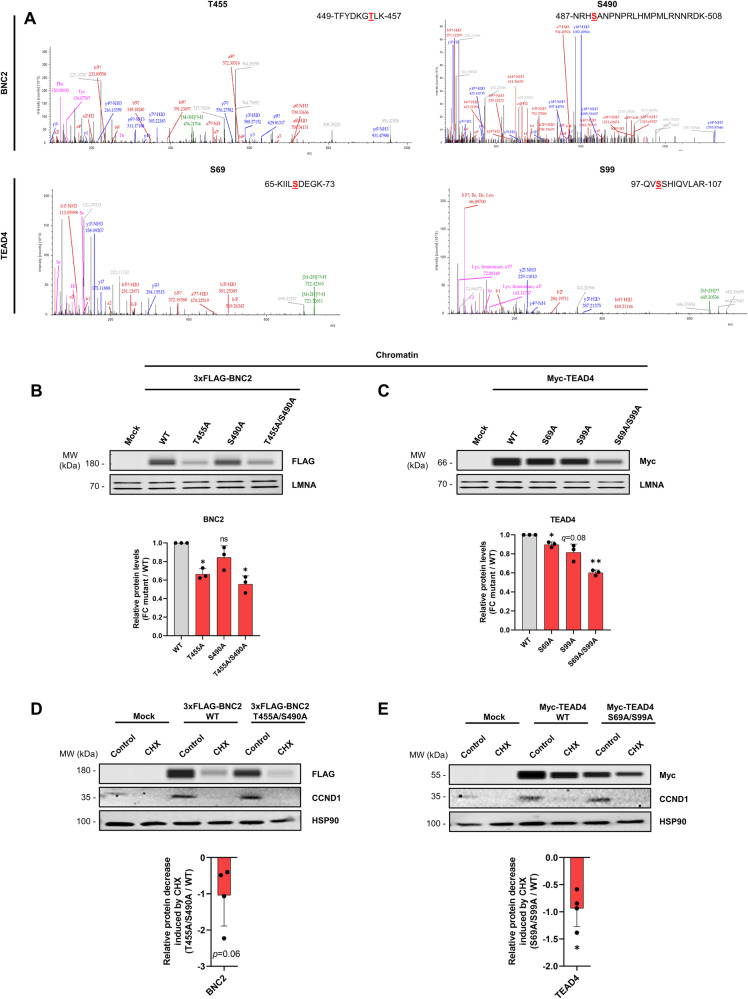
*O*-GlcNAcylation of BNC2 and TEAD4 regulates their stability and chromatin binding. **A** LX-2 cells were treated with 2 µM Thiamet-G for 24 h before BNC2 and TEAD4 immunoprecipitations followed by HCD mass spectrometry. HCD-MS/MS spectra of peptides covering the T455 and S490 *O*-GlcNAcylated sites in BNC2 and the S69 and S99 *O*-GlcNAcylated sites in TEAD4 are shown. The identified *O*-GlcNAcylated amino-acid residues are indicated in red in the peptide sequence. **B**, **C** LX-2 cells were transfected for 24 h with a control plasmid (mock), or plasmids encoding wild-type (WT) or mutated versions of 3x-FLAG-BNC2 (**B**) and Myc-TEAD4 (**C**). Sub-cellular fractionation was performed to obtain cytosolic and chromatin fractions used for western blot or simple western immunoassays using anti-FLAG (**B**) or anti-Myc (**C**) antibodies. The presented images are representative of three biologically independent experiments. Bar graphs show relative levels of chromatin-bound 3xFLAG-BNC2 (**B**) and Myc-TEAD4 (**C**). LMNA was used as protein loading control. Fold changes (FC) relative to WT (arbitrarily set to 1) are shown. Two-sided one-sample t-test with Benjamini-Hochberg correction for multiple testing was used to determine if the mean log_2_ FC between individual mutants and control was statistically different from 0. MW, molecular weight markers. **D**, **E** LX-2 cells were transfected as in **B**, **C** and cells were treated or not with 50 µg/mL cycloheximide (CHX) for an additional 24 h. Extracts were analyzed by western blot or simple western immunoassays using anti-FLAG (**D**), anti-Myc (**E**) or anti-CCND1 antibodies. The presented images are representative of 4 biologically independent experiments. Bar graphs show quantification of 3xFLAG-BNC2 and Myc-TEAD4 levels. HSP90 was used as protein loading control. Bar graphs show relative protein decrease induced by CHX which were calculated by subtracting the log_2_ FC CHX / control of double mutants by that of WT proteins. Unpaired t-test was used to assess the statistical significance of differences in log_2_ FC CHX / control between double mutants and WT proteins. MW, molecular weight markers. For all panels, the bar graphs show means + SD together with individual biological replicates.

To assess a direct role for *O*-GlcNAcylation in the control of BNC2 and TEAD4, we searched for *O*-GlcNAcylated sites on these proteins, which were immunoprecipitated from LX-2 cells treated for 24 h with 2 µM Thiamet-G, an OGA inhibitor to stabilize *O*-GlcNAcylation, and analyzed using HCD-MS/MS. This revealed that BNC2 was *O*-GlcNAcylated at Thr^455^ and Ser^490^ and TEAD4 at Ser^69^ and Ser^99^ (Fig. 8A and Supplementary Fig. 9). We next generated a set of mutants substituting the *O*-GlcNAcylated amino-acid residues by alanines in plasmids allowing for expression of FLAG-tagged BNC2 or Myc-tagged TEAD4. When expressed in LX-2 cells, BNC2 and TEAD4 mutants, especially proteins carrying a double mutation of the 2 *O*-GlcNAcylated amino-acid residues, exhibited reduced levels of chromatin-bound proteins when compared to the wild-type form (Fig. 8B, C). In line, we found that the double mutants BNC2 T455A/S490A and TEAD4 S69 A/S99A were less stable than their respective wild-type proteins as revealed by a greater decrease in protein levels following cell treatment with CHX (Fig. 8D, E). Altogether, these data confirmed that *O*-GlcNAcylation directly controls BNC2 and TEAD4 by promoting their stability and chromatin binding.

**Fig. 9:**
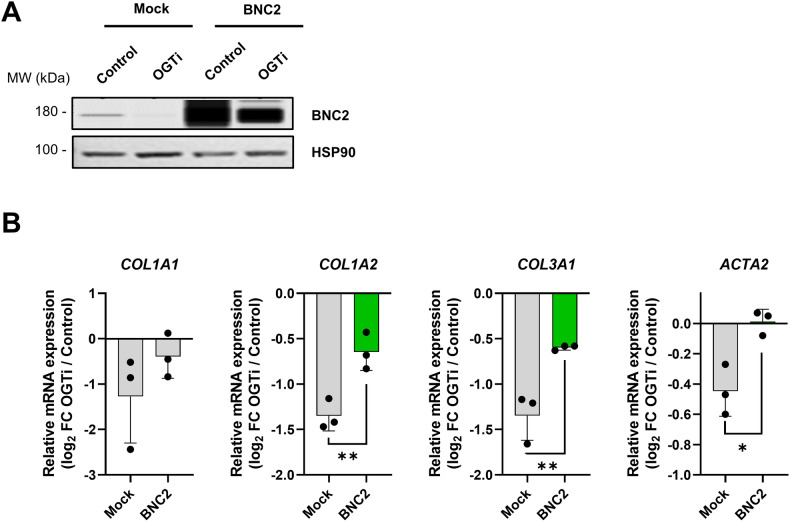
Forced expression of BNC2 dampens OGTi-mediated decrease in collagen-encoding gene expression in MF-HSCs. **A** LX-2 cells were transfected with a control plasmid (mock) or 3x-FLAG-BNC2 encoding plasmid for 24 h. Then, cells were treated or not with 50 µM OSMI-1 (referred to as OGTi) for an additional 24 h and used to prepare extracts analyzed using western blot and simple western immunoassays. BNC2 levels were assessed and HSP90 was used as a protein loading control. The presented images are representative of 3 biologically independent experiments. MW, molecular weight markers. **B** LX-2 cells were transfected as in **A** and used for RT-qPCR analyses of indicated gene expression (*n* = 3 biologically independent experiments). The bar graphs show means + SD together with individual biological replicates. Log_2_ FC between OGTi and control conditions are shown. Unpaired t-test was used to assess the statistical significance between 3x-FLAG-BNC2 expressing cells and the control (mock) condition.

We finally assessed whether forced expression of *O*-GlcNAcylation-dependent TFs could alleviate the effects of OGTi treatment on collagen-encoding gene expression in LX-2 cells. BNC2 overexpression was dampened by OGTi treatment but levels remained higher when compared to endogenous BNC2 (Fig. 9A). In these conditions, the inhibitory effect of OGTi on collagen-encoding gene expression was significantly reduced (Fig. 9B). Overexpression of TEAD4 did not have such an impact (Supplementary Fig. 10), which does not rule out a specific contribution for TEAD4 in mediating *O*-GlcNAcylation-dependent gene regulation of other fibrogenic target genes. Alternatively, this may also be explained by *O*-GlcNAcylation regulating additional properties of this TF besides its stability impeding TEAD4 overexpression from fully restoring its activity in OGTi-treated cells.

Altogether, our data reveal an essential role for *O*-GlcNAcylation in promoting establishment of the MF-HSC transcriptome through direct control of key MF-HSC transcriptional regulators.

## Discussion

**Fig. 10:**
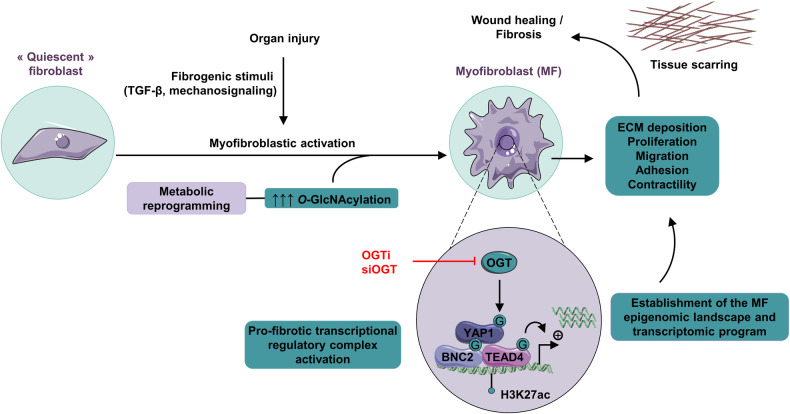
Summary of the key findings of this study. Schematic depicting how *O*-GlcNAcylation connects metabolic and transcriptomic reprogramming driving myofibroblastic activation during organ repair or fibrosis by targeting key MF TRs such as BNC2, TEAD4, and YAP1. Notably, *O*-GlcNAcylation plays a critical role in stabilizing the BNC2, TEAD4, and YAP1 proteins, enabling the assembly of pro-fibrotic transcriptional regulatory complexes required for establishment of the active epigenome and transcriptome of MFs.

Our study establishes that OGT-mediated protein *O*-GlcNAcylation functionally connects the metabolic and transcriptomic reprogramming events which initiate and perpetuate myofibroblastic activation (Fig. 10). Our comprehensive assessment of the levels and role of OGT-mediated *O*-GlcNAcylation in MF-HSCs through a compendium of approaches including multi-omics analyses and usage of a large array of biological models and samples unambiguously demonstrates its requirement for myofibroblastic activation (Fig. 10). Indeed, we demonstrate that protein *O*-GlcNAcylation is required for pan-MF archetypal activities such as collagen secretion, cell proliferation, adhesion, contraction and wound healing capacity (Fig. 10). Our results, together with previous studies showing that *O*-GlcNAcylation is also instrumental for immune cell activation [59, 60] and carcinogenesis [61], suggest that increased *O*-GlcNAcylation is a hallmark of pathophysiological cell (re)activation.

Through a combination of transcriptomic, epigenomic and proteomic-based approaches, we identified a critical role for protein *O*-GlcNAcylation in controlling the myofibroblastic regulatory chromatin landscape and gene expression program by licensing the activity of key MF TRs. Recent characterization of cellular *O*-GlcNAcome has established that *O*-GlcNAcylated proteins are enriched in the nucleus, which we observed as well in primary mouse MF-HSCs (Fig. 1D, E), and was linked to TRs being main targets of this PTM [62]. Altogether with our study, this points to control of TRs as a main mechanism underlying *O*-GlcNAcylation-mediated biological effects. More specifically, leveraging the first analysis of the MF *O*-GlcNAcome, we uncovered that *O-*GlcNAcylation of the key MF TFs BNC2 and TEAD4, in addition to that of their cofactor YAP1, was instrumental. Indeed, *O*-GlcNAcylation is required to stabilize the BNC2, TEAD4, and YAP1 proteins in order to promote formation of a transcriptional regulatory complex required for establishment of the active epigenome and transcriptome of MFs. In addition to mediating mechano-signaling through the Hippo/YAP1 pathway, BNC2 [6] and TEADs [63] also act downstream of additional myofibroblastic canonical activation pathways such as TGF-β signaling, which similarly requires protein *O*-GlcNAcylation (Supplementary Fig. 11). Our data do not rule out a contribution of the many other *O*-GlcNAcylated proteins in MFs or a complementary control at the chromatin level through histone modifications. However, considering that BNC2 [6], TEAD4 (this study) and YAP1 [38, 64] are individually critical for myofibroblastic activation, our study has uncovered an important mechanism of action of *O*-GlcNAcylation, which is therefore revealed here as a central licenser of transcriptional regulatory signaling driving MF identity (Fig. 10).

Our findings further support the potential interest of targeting cell-intrinsic metabolism to intervene with MF activities in organ fibrosis [65–67]. OGT inhibition using OSMI-1, previously shown to trigger beneficial effects in several preclinical mouse models of diseases such as inflammatory arthritis [68–70], is reported here to impede fibrogenic MF activation in murine models of liver injury. We could observe OSMI-1 mediated inhibition of MF-HSCs despite the reported role of OGT in protecting hepatocytes from loss of identity and necroptosis [71, 72]. However, considering *O*-GlcNAcylation inhibition as a viable clinical anti-fibrotic strategy would require further development of tools for targeted drug delivery to MFs [73] to avoid any potential detrimental effects in other cell types. In this context, approaches that allow to erase *O*-GlcNAcylation on specific proteins [74] could also be leveraged to specifically target MFs by modulating *O*-GlcNAcylation of the pro-fibrotic TRs BNC2, TEAD4, and/or YAP1. Interestingly, strategies based on nanobody-fused split *O*-GlcNAcase (OGA) to enhance OGA recruitment to the desired target protein have already yielded successful results in living cells [75].

In conclusion, our study defines that *O*-GlcNAcylation, through the control of key MF TRs, is required to drive the epigenomic and transcriptional reprogramming involved in myofibroblastic activation. *O*-GlcNAcylation inhibition therefore appears to be a potential promising therapeutic approach for the treatment of fibrosis.

## Materials and methods

### Resources and reagents

Details about resources, reagents, and tools used in this study are provided in Supplementary Table 7.

### Animal studies

Mice were housed in standard cages in a temperature-controlled room (22-24°C) with 12 hours (h) light-dark cycles and provided with water and standard diet ad libitum. Mice were allowed to acclimate for at least 1 week (wk) prior to any experiment. To chemically induce liver fibrosis, wild-type (WT) C57BL/6 J mice (males, 15 wks old, Charles River Laboratories, Wilmington, MA, USA) were intraperitoneally injected with 0.5 mL/kg CCl_4_ (Sigma-Aldrich, St. Louis, MO, USA) diluted in olive oil three times a wk for 2 wks. 7 h after the last injection, mice were intraperitoneally injected with 1 mg OSMI-1 (referred to as OGTi, Sigma-Aldrich) solubilized in 4.5% DMSO and 5% Tween-80 for overnight. Mice were fasted 4 h before livers and blood (sampled from the retro orbital sinus) were collected. To ensure that there were no excessive side effects of the drug, body weight was measured every other day (d) throughout the course of experiments.

Plasma Alanine aminotransferase (ALT) and Aspartate aminotransferase (AST) activities were measured on a Konelab 20 (Thermo Scientific^TM^, Waltham, MA, USA) with reagents for ALT/GPT and AST/GOT (IFCC) (Thermo Scientific^TM^).

### Human samples

Liver samples with alcohol-related cirrhosis were obtained from 10 patients enrolled in the TargetOH cohort (“Comparison of Inflammatory Profiles and Regenerative Potential in Alcoholic Liver Disease”; ClinGov NCT03773887; and DC-2008-642) [76–78]. These patients had decompensated alcoholic-related cirrhosis and underwent liver transplantation at Huriez Hospital’s Liver Unit (Lille, France). All liver samples were promptly fixed for histology. The study received authorization from the Lille ethical committee (Lille University Hospital), and informed consent was obtained from all subjects.

Cardiac tissues were collected from 4 patients ( > 18 years old) enrolled in the POMI-AF cohort (“Post-Operative Myocardial Incident & Atrial Fibrillation”; NCT03376165). These patients were referred to the Lille University Hospital (Lille, France) for coronary artery bypass surgery between April and May 2022. Biopsy of the right atrium was taken during the procedure and preserved in cold PBS (Phosphate-Buffered Saline). The study received authorization from the institutional ethics committee (Comité de Protection des Personnes Ile de France V) and all patients provided their medical history and gave informed consent.

### Primary cell isolation

Primary mouse HSCs were isolated from WT C57BL/6 J mice (male, 15–18 wks old, Charles River Laboratories) according to the protocol described in Bobowski-Gerard et al. [6]. Briefly, livers were in situ perfused with 14 mg protease XIV (Sigma-Aldrich) and 3.7 U collagenase D (Roche, Bâles, Swiss) and subsequently in vitro digested with 0.5 mg/mL protease XIV, 0.088 U/mL collagenase D and 0.02 mg/mL DNase I (Roche). HSCs were separated from other hepatic cell populations with a Nycodenz^®^ (Serumwerk Bernburg, Bernburg, Germany) density gradient and sorted on a FACS Aria II SORP (BD Biosciences, Franklin Lakes, NJ, USA) based on retinol autofluorescence by excitation with a 355 nm laser and detection with a 450/50 nm band-pass filter. The extremely high purity of HSCs obtained using this procedure has already been verified [6].

WT C57BL/6 J mice (male, 6 wks old, Charles River Laboratories) intraperitoneally injected with 0.5 mL/kg for 24 h were used to sort multiple mouse liver cell types following the procedure described in Zummo et al. [79].

Primary human cardiac MFs were isolated according to the protocol described in Nagaraju et al. [80]. Briefly, human atrium tissue was finely-minced using scissors followed by washing in Tyrode’s solution (137 mmol/L NaCl, 5.4 mmol/L KCl, 0.5 mmol/L MgCl_2_, 1.8 mmol/L CaCl_2_, 11.8 mmolL/L Na-HEPES, 10 mmol/L glucose), digestion in Ca^2+^-free Tyrode’s solution (137 mmol/L NaCl, 5.4 mmol/L KCl, 0.5 mmol/L MgCl_2_, 11.8 mmolL/L Na-HEPES, 10 mmol/L glucose) with 0.35 mg/mL protease XIV and 0.32 U/mL collagenase A (Roche) for 20 min and in Ca^2+^-free Tyrode’s solution with 0.21 U/mL collagenase A for 30 min. The digested tissue containing cardiac MFs and myocytes was mechanically dissociated using 19 G and 21 G needles and filtered using polyamide meshes (200 µm mesh size) to remove cell debris. After centrifugation at 160xg for 10 min at room temperature (RT), cell were resuspended and plated onto 24-well plates using at least 5×10^5^ cells/well. After 4 days of culture, unadhered myocytes were removed by a washing step allowing MFs purification.

### Cell culture and treatments

Murine primary HSCs and human HSC cell-line LX-2 (Merck, Darmstadt, Germany) were cultured in Dulbecco’s Modified Eagle Medium (DMEM, Gibco^TM^, Waltham, MA, USA) supplemented with 5% fetal bovine serum (FBS, Dutscher, Bernolsheim, France). Human primary HSCs (Samsara Sciences, Innoprot, and Huriez Hospital’s Liver Unit) (Supplementary Table 7) were grown in DMEM supplemented with 15% FBS and human primary cardiac MFs in DMEM supplemented with 10% FBS, 1 g/L glucose (Sigma-Aldrich), 1 mM sodium pyruvate (Gibco^TM^) and 25 mM HEPES (Gibco^TM^). Human LL29 lung MF cell line (Sigma-Aldrich) was grown into Nutrient Mixture F-12 Ham (Sigma-Aldrich) supplemented with 15% FBS and 2.68 g/L sodium bicarbonate. Spheroids were obtained by growing LX-2 cells (4 × 10^3^ cells) in U-bottom cell repellent 96-well plates (Greiner, Kremsmünster, Austria) for 72 h. Then, non-embedded spheroids (6.4 × 10^4^ cells) were harvested or transferred into classical polystyrene 12-well plate for an additional 72 h. All cells were cultivated in presence of 100 U/mL penicillin-streptomycin (Gibco^TM^) (and 40 µg/mL gentamicin (Gibco^TM^) for murine primary HSCs) at 37°C in a humidified atmosphere with 5% CO_2_.

Cells were treated with 50 µM OSMI-1 (OGTi, Sigma-Aldrich) or ST045849 (Tocris, Bristol, UK) added to the growth media for 24 h, unless specified in figure legends. To monitor the effect of OGTi on cell viability, cytotoxicity was assessed via the release of Lactate Dehydrogenase (LDH) using Cytotox 96^®^ Non-Radioactive Cytotoxicity Assay kit (Promega, Madison, WI, USA) and the trypan blue (Eurobio Scientific, Les Ulis, France) exclusion assays.

For TGF-β treatment, LX-2 cells were serum-starved for 24 h using DMEM (Gibco^TM^) supplemented with 0.5% FBS, 0.2% Bovine Serum Albumin (BSA, Sigma-Aldrich), 5.5 mM glucose (Gibco^TM^) and 1 mM sodium pyruvate, and treated with 1 ng/mL recombinant human TGF-β 1 protein (R&D Systems, Minneapolis, MN, USA) for 24 h in the starvation medium. For nutrient stimulation, LX-2 cells were grown in glucose-free DMEM (Gibco^TM^) supplemented with 5% FBS, 1 mM glucose and 1 mM sodium pyruvate for 8 h and in glucose-free DMEM supplemented with 0.5% FBS, 0.2% BSA and 1 mM sodium pyruvate for additional 24 h. Then, cells were incubated with or without 5 mM glucosamine (Sigma-Aldrich) or 11 mM glucose for 24 h in the starvation medium. For cycloheximide (CHX, Acros Organics, Geel, Belgium) experiments, LX-2 cells were pre-treated with 20 µg/mL CHX for 1 h in growth medium and then, 50 µM OGTi was directly added to the medium for additional 24 h.

According to the manufacturer’s instructions, LX-2 cells were transfected with siRNA (used at 20 nM) using JetPrime^®^ (Polyplus-transfection, Illkirch, France) for 72 h prior to harvest. Control and target siRNAs directed against *OGT* and *TEAD4* were purchased from Ambion (Huntingdon, UK) and Horizon Discovery (Waterbeach, UK), respectively. Transfection of pGL3-basic (Promega), 8xGTIIC-luciferase (Addgene, Watertown, MA, USA) and pRL-CMV (Promega) plasmids in LX-2 cells was performed using FuGENE^®^ HD Transfection Reagent (Promega). 72 h after transfection, promoter activity was determined for firefly and *Renilla* luciferase reporter activities with the Dual-Glo^®^ Luciferase Assay System (Promega). Transfection of pCMV-HA (Clontech; a kind gift from Satrajit Sinha, SUNY at Buffalo, Buffalo, NY, USA), 3xFLAG-BNC2 (E-Zyvec, Loos, France), pcDNA3 (Invitrogen^TM^, Waltham, MA, USA), Myc-TEAD4 (VectorBuilder, Neu-Isenburg, Germany) plasmids in LX-2 cells was performed using JetPEI^®^ (Polyplus-transfection, Illkirch, France) for 24 or 48 h according to the manufacturer’s instructions. Directed mutagenesis of 3xFLAG-BNC2 and Myc-TEAD4 was performed by VectorBuilder and validated through targeted sequencing.

Reagents used for cell culture treatments and plasmids used in these experiments are listed in Supplementary Table 7.

### Precision-cut liver slice studies

For acute OGTi treatment, livers of WT C57BL/6 J mice (3 females and 3 males, 17 wks old, Charles River Laboratories) were sliced in ice-cold Hanks’ Balanced Salt Solution (HBSS, Gibco^TM^) and cut with a vibratome tissue slicer (Campden Instruments, London, UK) at 200 µm thickness (80 Hz, 0.33 mm/s, 4°C). Slices were incubated on 30 mm Millicell cell culture insert (Millipore, Burlington, MA, USA) with glucose-free DMEM supplemented with 10% FCS, 25 mM glucose, 10 mM HEPES, 1% GlutaMAX^TM^ (Gibco^TM^), 4 mM sodium bicarbonate and 100 U/mL penicillin-streptomycin at 36°C in a humidified atmosphere without CO_2_. Fibrotic PCLS at 6 d of culture were treated with 50 µM OSMI-1 added to the growth media for 24 h. For prolonged OSMI-1 treatment, livers of *Per2*::luc C57BL/6 J mice (4 males, 18-22 wks old, IMSR_JAX:006852 [81, 82], The Jackson Laboratory re-derivated into SOPF C57BL/6 J mice at Charles River Laboratories) were used. This allowed to continuously monitor the viability of PCLS (maintained at 36 °C without CO_2_ into a Kronos-Dio (ATTO, Tokyo, Japan) luminometer system) by monitoring ex vivo luciferase activity using the Kronos software (v2.30.243) as previously described [83]. Treatment consisted in a 9 d-exposure to 50 µM OSMI-1 in the growth media, which was renewed every 3 days.

### RNA extraction and RT-qPCR

The anterior right lobe of mouse livers was systematically used for RNA extraction. Liver tissues were homogenized in RA1 buffer (Macherey-Nagel, Hoerdt, France) or Extract-All (Trizol) (Eurobio Scientific) and PCLS in LB1 buffer (Macherey-Nagel) with bulk 1.4 mm ceramic beads (FisherBrand^TM^, Waltham, MA, USA) using Minilys (Bertin Technologies, Montigny-le-Bretonneux, France). Total RNA was extracted using Nucleospin^®^ RNA kit (Macherey-Nagel) for cell-lines and liver tissues, and Nucleospin^®^ RNA Plus XS kit (Macherey-Nagel) for primary cells and PCLS. Depending on the RNA amount, RNA was reverse-transcribed using High-Capacity cDNA Reverse Transcription Kit (Applied Biosystems^TM^, Villebon-Sur-Yvette, France) or LunaScript^®^ RT SuperMix (New England Biolabs, Ipswich, MA, USA). RT-qPCR was performed using the Takyon^TM^ Low ROX SYBR 2X MasterMix blue dTTP (Eurogentec, Seraing, Belgium) on a QuantStudio 3 (Applied Biosystems^TM^). The specificity of amplification was checked by recording the dissociation curves, and the efficiency was verified to be above 90% for each primer pair. mRNA levels were normalized to the expression of housekeeping genes and the fold induction was calculated using the cycle threshold (ΔΔCT) method. The sequences of primers used are listed in Supplementary Table 7.

### Protein extraction

The posterior right lobe of mouse livers was systematically used for protein extraction. Liver tissues were homogenized in Penny’s extraction buffer (100 mM Tris-HCl, pH 7.4, 100 mM NaCl, 2 mM EDTA, 0.10% Triton X-100) supplemented with an enzymatic activity inhibitor cocktail (50 mM sodium fluoride, 1 mM sodium orthovanadate, 1X cOmplete^TM^ EDTA-free protease inhibitor cocktail (Roche)) with bulk 1.4 mm ceramic beads using Minilys. Total cell extracts from LX-2 and murine primary HSCs were obtained by washing cells with PBS and scraping in high-salt lysis buffer (25 mM Tris-HCl, pH 7.5, 500 mM NaCl, 2 mM EDTA, 0.5% NP-40) supplemented with the enzymatic activity inhibitor cocktail. Tissues and cell lysates were then sonicated for 5 minutes (min) (1 cycle of 30 seconds (sec) ON/30 sec OFF) using Bioruptor (Diagenode, Seraing, Belgium) and insoluble material was removed by centrifugation at 16,000xg for 5 min at 4°C. Proteins from the chromatin fractions were prepared following the protocol described in Dubois et al. [84]. The cytosolic fraction from the same samples was preserved and subjected to immunoblotting analysis as well. Protein concentration was determined using Pierce^TM^ BCA Protein Assay Kit (Thermo Scientific^TM^).

### Protein immunoblotting

For western blotting assays, 20-30 µg of proteins were separated by 10% SDS-PAGE and immunodetected using the primary antibodies listed in Supplementary Table 7. Detection was achieved using peroxidase-conjugated secondary antibodies (Sigma-Aldrich). Images were acquired using the iBright^TM^ CL1500 Imaging System (Invitrogen) and quantification was performed using ImageJ (v1.42q, http://rsb.info.nih.gov/ij) by generating lane profile plots, drawing lines to enclose bands of interest and measuring peak areas.

For simple western immunoassays, protein extracts (0.4 µg/mL) were run on a Wes system (ProteinSimple) according to the manufacturer’s instructions. Separation was performed using the 12–230 kDa capillary cartridges. The chemiluminescence-based electrophoretograms were auto-generated, and digitally rendered bands were produced from the chemiluminescent peaks using the Compass software (ProteinSimple, Santa Clara, CA, USA). Quantifications were obtained using the area under the peak of the protein of interest.

All uncropped original images derived from western blotting or simple western immunoassays are shown in Supplementary Fig. 12.

### Co-immunoprecipitation assays

To prepare nuclear extracts, cells were washed with ice-cold PBS and lysed using hypotonic lysis buffer (20 mM Tris-HCl pH 8.0, 10 mM NaCl, 3 mM MgCl_2_, 0.2% NP40) for 5 min at 4 °C. After centrifugation (600xg for 5 min at 4°C), the pellet was resuspended in high-salt lysis buffer (25 mM Tris-HCl, pH 8.0, 500 mM NaCl, 2 mM EDTA, 0.5% NP-40) and incubated for 30 min at 4 °C. Subsequently, the lysate underwent 10 min of sonication using Bioruptor (1 cycle of 30 sec ON/30 sec OFF), and insoluble material was removed by centrifugation at 13,000×*g* for 10 min at 4 °C. One mg of nuclear extract was incubated with 5 µg of anti-TEAD4 (ab58310 from Abcam, Cambridge, UK), anti-YAP1 (D8H1X, Cell Signaling, Danvers, MA, USA; NB110-58358, Novus Biologicals, Centennial, CO, USA) or a control IgG (sc-2025 or sc-2027, Santa Cruz, Dallas, TX, USA) antibody overnight at 4 °C in a dilution buffer at a 1:2 volume ratio (25 mM Tris-HCl pH 8.0, 1 mM EDTA, 1.5 mM MgCl_2_). Next, 10 µl of a 1:1 mixture of Dynabeads^TM^ protein A and protein G magnetic beads (Invitrogen^TM^) blocked overnight in 5 mg/mL BSA in PBS were added and incubated 4 h at 4°C under agitation. Beads were washed 4 times with washing buffer (25 mM Tris-HCl pH 8.0, 150 mM NaCl, 1 mM EDTA, 0.2% NP-40). Proteins were eluted using 6x Laemmli buffer (175 mM Tris-HCl pH 6.8, 15% glycerol, 5% SDS, 300 mM DTT, and 0.01% Bromophenol Blue). All buffers were supplemented with an enzymatic activity inhibitor cocktail (50 mM sodium fluoride, 1 mM sodium orthovanadate and 1X cOmplete^TM^ EDTA-free protease inhibitor cocktail). Input proteins and immunoprecipitated materials were analyzed by western blot and simple western immunoassays.

### Protein and lipid droplet (immuno)staining

For *O*-GlcNAc staining in murine primary HSCs, cells were seeded in µ-Slide 8 well chambered coverslip (Ibidi, Fitchburg, Wisconsin, USA) and fixed 1 d (Q-HSC) or 7 d (MF-HSC) later with 4% paraformaldehyde (PFA) for 15 min. Samples were then washed with 0.05% Tween-20 in PBS (PBS-T), permeabilized with 0.25% Triton X-100 in PBS for 10 min, incubated with blocking buffer (5% BSA in PBS-T) for 15 min at RT and incubated overnight with anti-*O*-GlcNAc primary antibody (Abcam). Samples were finally incubated with Alexa Fluor^®^ 568-conjugated anti-mouse secondary antibody (Invitrogen^TM^) for 1 h at RT. Quantifications involved manually outlining each cell to define a region of interest (ROI), which was next used to define the integrated density (IntDen), i.e. the product of *O*-GlcNAc signal intensity and the area of the signal, using ImageJ software. The average signal per cell was defined using five randomly selected fields per biological replicate. Stress fibers in LX-2 cells were stained with CF^®^ 568-conjugated phalloidin (Biotium, Fremont, WCA, USA) for 20 min at RT. Quantification of cell spreading involved manually outlining each cell to define a ROI, which was then used to measure the signal area using ImageJ software. Quantification of the spreading area per cell was performed and averaged across four randomly selected fields per biological replicate. For lipid droplet staining, LX-2 cells were fixed in 2% PFA and 0.2% glutaraldehyde for 10 min, washed with PBS and incubated with BODIPY 493/503 (Invitrogen^TM^) at 1 µg/µL for 20 min at RT. Cells were imaged using a spinning disk confocal microscope (Zeiss, Baden-Württemberg, Germany) after nuclei counterstaining with Hoechst 33258 (Invitrogen^TM^). Images were processed with ZEN software (Zeiss). Number of lipid droplets was defined using the spot detector plugin in Icy software (v2.2.1.0, https://icy.bioimageanalysis.org) [85].

For PDGFRB and *O*-GlcNAc staining of human liver slices, samples were fixed with 4% PFA and embedded in paraffin. Four µm-thick sections underwent an antigen retrieval step with SignalStain® EDTA Unmasking Solution (Cell Signaling) for 21 min at 110°C into TintoRetriever Pressure Cooker (Bio SB, Santa Barbara, CA, USA). After each step, sections were washed in 0.05% Tween-20 in Tris-Buffered Saline (TBS) (TBS-T). They were sequentially incubated with 0.1% Triton X-100 in TBS for 10 min at 4°C, Bloxall® Endogenous Blocking Solution (Vector Laboratories, Newark, CA, USA) for 10 min at RT, 7% goat serum in TBS-T for 15 min at RT and then primary PDGFRB antibody (Cell Signaling) in TBS-T overnight at 4°C. Subsequently, sections were incubated for 30 min at RT with ImmPRESS® HRP Polymer Anti-Rabbit IgG Reagent (Vector Laboratories), followed by a TBS wash and a 10 min RT incubation with Alexa Fluor^TM^ 594 Tyramide SuperBoost^TM^ Reagent (Invitrogen^TM^) according to the manufacturer’s instructions. Following PDGFRB labeling, all preceding steps were repeated, this time using a primary anti-*O*-GlcNAc antibody (Cell Signaling) and Alexa Fluor^TM^ 488 Tyramide SuperBoost^TM^ Reagent. Stained slides were imaged using an Axioscan Z1 slide scanner (Zeiss) after nuclei counterstaining with DAPI (Thermo Scientific^TM^). Image processing was performed using ZEN software (Zeiss).

### Sirius red staining

For Sirius red staining of PCLS, samples were fixed with 4% PFA, embedded HistoGel^TM^ (Epredia^TM^, Portsmouth, NH, USA) and then in paraffin. Paraffin-embedded samples were cut at a thickness of 5 μm using a Leica RM2255 Fully Automated Rotary Microtome (Leica, Wetzlar, Germany) and transferred onto Superfrost^TM^ slides (Epredia^TM^). The staining procedure employed an Autostainer XL (Leica) with the following steps: xylene (2 min), xylene (2 min), 100% ethanol (2 min), tap water (2 min), 0.1% Direct Red 80 (Sigma-Aldrich) in a saturated picric aqueous solution (60 min), tap water (2 ×2 min), 0.0025% HCl in 70% ethanol (6 sec), tap water (2 min) and 100% ethanol (1 min). The entire stained slices were scanned using the Axioscan Z1 slide scanner and subsequent image processing was carried out using the ZEN software. Quantification was performed on ten randomly selected fields, with the exclusion of areas containing vessels.

### *O*-GlcNAc metabolic labeling

*O*-GlcNAc metabolic labeling was performed in quiescent (Q-HSC; 1 d of culture) and activated (MF-HSC; 7 d of culture) murine primary HSCs using the *O*-GlcNAc Modified Glycoprotein Assay Kit (Abcam). The kit uses an azido-modified glucosamine precursor (GlcAz) that is added into DMEM growth medium for 24 h, fed into cells, metabolized in the HBP pathway and incorporated into intracellular targeted proteins. After click reaction with 10 µM sulfo-Cy5 alkyne dye (BroadPharm^®^, San Diego, CA, USA) and nuclei counterstaining with Hoechst 33258, images were acquired using a spinning disk confocal microscope and processed with ZEN software. Average signal per cell performed as in “Protein and lipid droplet (immuno)staining” section was defined using ImageJ software using five randomly selected fields per biological replicate.

### HSC metabolic activity and proliferation

Metabolic activity was assessed via the reduction of 3-[4,5-dimethylthiazole-2-yl]−2,5-diphenyltetrazolium bromide (MTT) by mitochondrial dehydrogenases using the TACS MTT Cell Proliferation Assay (R&D Systems) used according to the manufacturer’s instructions. Proliferation and viability were evaluated by TC20 Automated Cell Counter (Bio-Rad, Hercules, CA, USA) using trypan blue (Eurobio Scientific) exclusion assays.

### Collagen secretion assays

After 24 h of culture with or without OSMI-1 or ST045849, the medium of LX-2 cells was collected and used with a Human Pro-Collagen I alpha 1 ELISA kit (Abcam) according to the manufacturer’s instructions.

### Cell adhesion assays

After pre-treatment or not with OGT inhibitors (OSMI-1 or ST045849) for 24 h, LX-2 cells were trypsinized and seeded (2×10^4^ cells) on collagen I (Sigma-Aldrich)-coated 24-well plate previously blocked with 1% BSA in PBS solution for 1 h. Cells were treated or not with OSMI-1 or ST045849 and/or 5 mM EDTA for 1 h and non-adherent cells were removed using 2 washes with PBS. Cells were fixed with 4% PFA for 15 min, washed with PBS and stained with Hoechst 33258. Cells were imaged using FLoid^TM^ Cell Imaging Station (Invitrogen^TM^). For quantification, the dye bound to DNA was eluted as described [86] using 2% SDS in PBS (pH 7.0) for 15 min. The elution solution was transferred to a white 96-well plate and the signal reflecting adherent cell number was measured using a plate reader (laser excitation at 355 nm and emission at 460 nm).

### Wound-healing assays

LX-2 cells were seeded (3.5×10^4^ cells) into culture-insert 2 wells (Ibidi) according to the manufacturer’s protocol. After 24 h, insert was removed and cells were treated or not with OSMI-1 for 0-48 h. Cells were imaged using a phase contrast microscope (Leica). Wound area was determined by measuring the distance between fronts of cell migration.

### Collagen matrix contraction assays

To prepare 1 mL of collagen gel solution, 772 µL collagen I at 3 mg/mL (Sigma-Aldrich) was mixed with 200 µL 5X DMEM medium (Sigma-Aldrich) supplemented with 5% FBS, 4.5 g/L D-glucose, 3.7 g/L sodium bicarbonate, 2 mM L-glutamine (Gibco^TM^), 100 U/mL penicillin-streptomycin and 28 µL 0.5 M NaOH. Then, LX-2 cell suspension (5×10^6^ cells) was mixed with the collagen gel solution at a ratio of 1:4 and transferred into a 24-well plate. After polymerization of the gel for 1 h at RT, a complete DMEM medium was added together with OSMI-1 or vehicle for 16 h. The collagen gel was then dislodged from the edge of the dish using a sterile filter tip and contractility measurements was conducted 8 or 24 additional h later by measuring the decrement in surface area of the gel. The diameter of the gel and the well within the top-view image were determined by edge detection and the surface area of the gel was determined in relation to the fixed diameter of the well (15.6 mm).

### Hydroxyproline measurement

PCLS (a pool of 3 PCLS per mouse liver) were weighed before hydrolysis with 150 µL of 6 M HCl at 95°C for 20 hours. The hydrolyzed samples were assessed for hydroxyproline content using the QuickZyme Sensitive Tissue Collagen Assay (QuickZyme Biosciences, Leiden, The Netherlands) according to the manufacturer’s protocol. Hydroxyproline content (µg) was normalized based on the weight of the slices.

### Chromatin immunoprecipitation-seq

ChIP was performed as described previously [6] on chromatin samples from LX-2 cells treated or not with 50 µM OSMI-1 for 24 h (3 or 4 independent experiments). For cross-linking, 15×10^6^ LX-2 cells were fixed for 10 min at RT with 1% formaldehyde (FA) (Thermo Scientific^TM^) in PBS followed by 5 min incubation with 125 mM glycine (Invitrogen^TM^). After two washes with ice-cold PBS, cells were scraped in PBS and incubated for 10 min in 0.25% Triton X-100, 10 mM EDTA, 10 mM HEPES and 0.5 mM EGTA buffer followed by 10 min in 0.2 M NaCl, 1 mM EDTA, 10 mM HEPES, 0.5 mM EGTA buffer. Then, nuclei were resuspended in lysis buffer (50 mM Tris-HCL pH 8.0, 10 mM EDTA, 1% SDS) and sonicated for 30 min (5 cycles of 30 sec ON/30 sec OFF) using Bioruptor. All buffers were supplemented with an enzymatic activity inhibitor cocktail (50 mM sodium fluoride, 1 mM sodium orthovanadate, 1X cOmplete^TM^ EDTA-free protease inhibitor cocktail). 30 µg of chromatin were taken prior to immunoprecipitation and referred to as the input, and 30 µg (for H3K27ac) or 200 µg (for TEAD4) of chromatin were diluted ten-fold in dilution buffer (20 mM Tris-HCl pH 8.0, 150 mM NaCl, 1% Triton X-100, 2 mM EDTA) and incubated overnight at 4°C with 2 µg of anti-H3K27ac (Active Motif 39685, Carlsbad, CA, USA) or 3 µg of anti-TEAD4 (Abcam ab58310) antibody. The next day, 40 µL of a 1:1 mixture of protein A and protein G Sepharose beads (GE Healthcare, Chicago, Illinois, USA) were added and samples were incubated for 4 h at 4°C under agitation with 70 µg/mL yeast tRNA (Sigma-Aldrich). Beads were washed 3 times with RIPA buffer (50 mM HEPES, 1 mM EDTA, 0.7% sodium deoxycholate, 1% NP-40, 500 mM LiCl) containing 10 µg/mL yeast tRNA and once with TE buffer (10 mM Tris-HCl pH 8.0, 1 mM EDTA). For reverse cross-linking, DNA from ChIP and input samples was eluted in RCL buffer (1% SDS, 100 mM NaHCO_3_) supplemented with 200 µg/mL Proteinase K Solution (Thermo Scientific^TM^) and incubated overnight at 65°C. DNA purification was performed using MinElute PCR purification kit (Qiagen, Hilden, Germany). ChIP and input samples were subjected to high-throughput sequencing and analyzed as described hereafter.

### Column Purified chromatin-seq

CoP-seq procedure was adapted from Zhang et al. [52] and performed on chromatin samples from LX-2 cells treated or not with 50 µM OSMI-1 for 24 h. In brief, 15×10^6^ LX-2 cells were cross-linked with 2% FA and lysed and sonicated as described for ChIP protocol. Thirty µg of chromatin were taken prior to DNA purification and referred to as the input. Soluble CoP chromatin was purified using the MinElute PCR purification kit. For reverse cross-linking, DNA from CoP and input samples was eluted in TE buffer supplemented with 1.25 mg/mL RNase A (Qiagen) for 30 min at 37°C followed by incubation with 1.25 mg/mL proteinase K for 30 min at 55°C, then overnight at 65°C. Finally, DNA was purified using MinElute PCR purification kit, and CoP and input samples were subjected to high-throughput sequencing and analyzed as described hereafter.

### ChIP-seq and CoP-seq data analysis

After initial quality controls of fastq files, raw data were mapped to hg38 using Chromap (v0.1.3-R256) [87] and the following parameters: error threshold: 8; min-num-seeds: 2; max-seed-frequency: 500-1000; max-num-best-mappings: 1; max-insert-size: 1000; MAPQ-threshold: 10; min-read-length: 30; bc-error-threshold: 1; bc-probability-threshold: 0.90.

Bam files for H3K27ac ChIP-seq data (n = 4 biological replicates) were next used for peak calling in each replicate using model-based analysis of ChIP-seq version 2 (MACS2 v2.1.1.20160309) [88] in a local instance of Galaxy [89]. Input DNA was used as control, duplicated reads were removed together with those mapping to ENCODE Blacklisted regions (v2) [90]. Parameters used were: Band width for picking regions to compute fragment size: 300; Set lower mfold bound: 5; Set upper mfold bound: 50; Peak detection based on: *q-*value; Minimum false discovery rate (FDR *q*-value) cutoff for peak detection: 0.05; Build Model: nomodel; Set extension size: 200; Set shift size: 0. Bam files and bed files issued from the peak calling analyses of the 8 datasets were used for data normalization and identification of regions with differential H3K27ac enrichment between OSMI-1-treated and control cells using MAnorm2 [91]. Profile bins was performed using the following parameters: typical-bin-size: 2000; shiftsize: 100; min-peak-gap: 500. Fitting a smooth mean-variance curve was performed with replicates from OSMI-1 treated cells and parameters subsequently applied to replicates from control cells (parameters were method: “parametric”; occupy.only: TRUE; max.iter=1000; range.residual: c(1e-04,15)). Finally, MAnorm2 was used to test for differential H3K27ac signals between OSMI-1-treated and control cells in peaks (the adjusted *p*-value was set at 0.05). To get signal tracks of normalized H3K27ac ChIP-seq data, normalization coefficients defined by MAnorm2 were retrieved and applied to original Bam files. Average normalized signal tracks for H3K27ac ChIP-seq in OSMI-1-treated and control cells using multiBigwigSummary of the deepTools (v2.0) [92].

Bam files for TEAD4 ChIP-seq data (*n* = 3 biological replicates) were analyzed using the Irreproducible Discovery Rate (IDR), which allows to identify TF binding peaks that are reproducible and rank-concordant across replicates [93] This was performed using the IDR pipeline from ENCODE (v2.2.1) installed from https://github.com/ENCODE-DCC/chip-seq-pipeline2/tree/master [94]. IDR identified that only 2 replicates were concordant enough (replicates 1 and 3), which were therefore used to re-run IDR and identify TEAD4 binding peaks and obtain a TEAD4 ChIP-seq signal track.

CoP-seq raw data and mapping to hg38 were performed similarly to the ChIP-seq data. Signal tracks were obtained using the bamCoverage function of the deepTools with the following parameters: -bs 25 --normalizeUsing CPM -e 200.

Heatmaps of average ChIP-seq or CoP-seq signals were generated using the deepTools using the compute matrix and heatmap functions (parameters were set to “reference-point”, “center of regions”, “2500” for distance upstream and downstream).

In order to identify TRs whose binding significantly overlap with regions enriched for H3K27ac in OSMI-1-treated cells (versus all regions with H3K27ac signal), we used Locus Overlap Analysis (LOLA v1.4.0) [95]. ChIP-seq datasets used in these analyses were retrieved from ReMap2022 [53] enriched with our recently defined BNC2 cistrome in LX-2 cells (n = 8,112 cistromes). The most significantly overlapping cistromes were recovered using the following filters: *p*ValueLog > 20, oddsRatio>2 and support>100.

### Transcription factor recognition motif enrichment analyses

JASPAR motifs enrichment was defined using TFmotifview (default parameters including “Generate random matched control regions”) [96]. De novo motif enrichment analyses were performed using RSAT [97]. Sequences were retrieved from bed file using the “Retrieve sequences from genomic coordinates” function and motif enrichments defined by the “RSAT peak-motifs” tool used default parameters including comparison with Hocomoco human TF recognition database. Analyses of motifs enriched in regions loosing H3K27ac were performed using all other regions with gain or unchanged H3K27ac as control.

### Gene assignation to H3K27ac enriched regions

H3K27ac enriched regions mapping within 2.5 kilobases (kb) of an annotated gene transcriptional start site (TSS) from the Gencode v34 database were assigned to these genes. Additionally, H3K27ac enriched regions were also assigned genes based on predicted promoter interactions with distal regulatory regions within the entire FOCS datasets (http://acgt.cs.tau.ac.il/focs/download.html) [98].

### Identification and characterization of *O*-GlcNAcylated proteins

LX-2 cells were treated for 24 h with 2 µM Thiamet-G (Focus Biomolecules, Plymouth Meeting, PA, USA), an OGA inhibitor, to preserve *O*-GlcNAcylated sites (3 independent experiments). Nuclear protein extracts were obtained by washing cells with PBS and scraping them in hypotonic lysis buffer (20 mM Tris-HCl pH 7.5, 10 mM NaCl, 3 mM MgCl2, 0.2% NP-40). After centrifugation (16,000xg for 10 min), cell pellets were further incubated with high-salt lysis buffer (25 mM Tris-HCl, pH 7.5, 500 mM NaCl, 1 mM EDTA, 0.5% NP-40). Cell lysates were then sonicated for 5 min (1 cycle of 30 sec ON/30 sec OFF) using Bioruptor, centrifugated at 16,000xg for 10 min and supernatants collected as the nuclear extracts. All buffers were supplemented with an enzymatic activity inhibitor cocktail (50 mM sodium fluoride, 1 mM sodium orthovanadate, 1X cOmplete^TM^ EDTA-free protease inhibitor cocktail, 2 µM Thiamet-G). Labeling of *O*-GlcNAcylated proteins by GalNAz and enrichment on alkyne agarose resin were performed on nuclear-enriched protein extracts (1.25 mg for each sample) using the Click-iT™ *O*-GlcNAc Enzymatic Labelling System (Invitrogen^TM^) and the Click-iT™ Protein Enrichment Kit (Invitrogen^TM^) according to the manufacturer’s instructions. To remove N-glycans and improve specificity of the *O*-GlcNAc enrichment, 7500 U/mg PNGase F (New England Biolabs) were added in the Click-iT™ enzymatic labelling reaction. A negative control sample obtained using proteins incubated with Click-iT™ enzymatic labelling reaction without UDP-GalNAz substrate donor was prepared in parallel. The digestion buffer used to digest the resin-bound *O*-GlcNAcylated proteins was composed of 1 µg Trypsin/Lys-C mix (Promega), 100 mM triethylammonium bicarbonate buffer (TEAB, pH 8.2) (Sigma-Aldrich) and 10% acetonitrile (ACN). After overnight incubation at 37°C and dry evaporation, peptides were resuspended in 10 µL of 0.1% formic acid (FA) before Liquid Chromatography coupled to tandem Mass Spectrometry (LC-MS/MS) analysis.

LC-MS/MS was performed on an Orbitrap Q Exactive Plus Mass Spectrometer (Thermo Scientific^TM^) hyphenated to a U3000 RSLC Microfluidic HPLC System (Thermo Scientific^TM^). 1 μL of the peptide mixture was injected with a solution A (5% v/v ACN and 0.1% FA) for 3 min at a flow rate of 5 μL/min on an Dionex^TM^ Nano-Trap^TM^ pre-column (30 μm i.d. × 100 mm, Thermo Scientific^TM^). The peptides were next separated on a Acclaim^TM^ PepMap^TM^ 100 C18 reversed phase column (2 μm, 75 µm i.d. × 500 mm, Thermo Scientific^TM^), using a linear gradient (5-40%) of solution B (75% ACN and 0.1% FA) using a flow-rate of 250 nL/min in 140 min followed by 100% solution B for 5 min. The column was regenerated by washing it for 5 min with solution B and then re-equilibrated with solution A during 10 min. The column and the pre-column were placed in an oven at a temperature of 45°C. The total duration of the analysis was 140 min. The LC runs were acquired in positive ion mode. MS scans for DDA were acquired from *m/z* 350 to 1500 in the Orbitrap mass analyzer with a 70,000 resolution with maximum injection time of 90 ms and AGC target of 1e^6^. MS/MS scans were sequentially acquired in the high-energy collision dissociation cell for the 10 most-intense ions detected in the full MS survey scan. For MS/MS the resolution was set to 17,500 with maximum injection time of 150 ms and AGC target of 5e^5^ and the normalized collision energy was set to 30 eV. Dynamic exclusion was set at 60 s and ions with 1 and more than 8 charges were excluded.

Analysis of raw data from LC-MS/MS was performed using Proteome Discoverer version 2.2 (Thermo Scientific^TM^). SEQUEST search engine was used for database searching against *Homo sapiens* from UniProtKB reviewed Swiss-Prot (20,404 sequences, 2022). The MS error was set to 10 ppm and the MSMS mass tolerance was set to 0.01 Da. Enzyme specificity was selected to trypsin. Trypsin was selected with specific cleavage site (K, R), together with variable modifications acetylation (+42.011 Da, K), oxidation (+15.995 Da, M, P), deamidation (+0.984 Da, N, Q), *O*-GlcNAc (+203.079 Da, S, T), and with the fixed modification carbamidomethyl cysteine (+57.021 Da). The minimal peptide length was set to six amino-acids and the maximum number of missed cleavages to fives. The FDR threshold was set to 0.05 using Percolator node.

Only proteins detected in at least 2 out of 3 biological replicates, but not in the negative control sample nor in the Contaminant Repository for Affinity Purification (human CRAPome2.0) database [99], were considered. Human transcription factors and cofactors were identified using the AnimalTFDB 3.0 database [100] (http://bioinfo.life.hust.edu.cn/AnimalTFDB).

To complement these analyses, *O*-GlcNAcylation of proteins of specific interest (including. BNC2, TEAD4, and YAP1) was assessed. LX-2 cells were treated with 2 µM Thiamet-G for 24 h. The labeling of *O*-GlcNAcylated proteins by GalNAz was performed on nuclear-enriched protein extracts (200 µg) using the Click-iT™ *O*-GlcNAc Enzymatic Labelling System and the Click-iT™ Biotin Protein Analysis Detection Kit (Invitrogen^TM^) according to the manufacturer’s instructions. As a negative control sample, proteins were simultaneously subjected to the Click-iT™ enzymatic labelling reaction without the UDP-GalNAz substrate donor. Labeled proteins were then incubated with 60 µL of Dynabeads^TM^ M-280 Streptavidin magnetic beads for 1.5 h at 4°C in 1% Triton X-100 in PBS buffer under agitation. Beads were washed 3 times within 1% Triton X-100 in PBS buffer and enriched proteins were eluted using 6x Laemmli buffer. Both input proteins and enriched materials were analyzed by western blot and simple western immunoassays (Supplementary Fig. 7).

*O*-GlcNAcylated TRs in LX-2 MF-HSCs identified in our study are provided in Supplementary Table 6.

Finally, for the identification of *O*-GlcNAcylated amino-acid residues, LX-2 cells were treated for 24 h with 2 µM Thiamet-G and nuclear protein extracts were obtained as for co-immunoprecipitation assays. Immunoprecipitation was performed using 5 µg of antibody directed against BNC2 (55220-1-AP from Proteintech) or TEAD4 (ab58310 from Abcam) and 1 mg of nuclear extract which were incubated at 4°C overnight under agitation in dilution buffer (25 mM Tris-HCl pH 8.0, 1 mM EDTA, 1.5 mM MgCl_2_). Then, 50 µL of a 1:1 mixture of Dynabeads^TM^ protein A and protein G magnetic beads blocked overnight in 5 mg/mL BSA in PBS was added and incubated 4 h at 4 °C under agitation. Beads were washed 4 times with washing buffer (25 mM Tris-HCl pH 8.0, 150 mM NaCl, 1 mM EDTA, 0.2% NP-40). Peptides were digested within digestion buffer as described above and then resuspended in 10 µL of 0.1% FA before undergoing LC-MS/MS analysis.

### Transcriptomic analyses

Transcriptomes were determined using RNA-seq performed on RNA samples from primary murine HSCs treated or not with 50 µM OSMI-1 for 24 h (4 independent experiments) and LX-2 cells treated or not with 50 µM OSMI-1 for 24 h or transfected with 20 nM siOGT or siControl for 72 h (4 and 3 independent experiments, respectively). Purified RNA integrity and quantity were evaluated using Agilent 2100 Bioanalyzer (Agilent Technologies, Santa Clara, CA, USA) before being used for high-throughput sequencing. Paired-end sequencing was performed on RNA-seq libraries obtained after oligo dT based enrichment. Data were analyzed using FastQC (http://www.bioinformatics.babraham.ac.uk/projects/fastqc) and reads were trimmed when necessary. Mapping of reads to the human or murine genome (hg38 or mm39, respectively) was achieved using Bowtie2 (v2.3.5.1) [101] using the GENCODE annotation (GRCh38.84 or GRCm39, respectively) and with the following settings: -q --phred64 --sensitive --dpad 0 --gbar 99999999 --mp 1,1 --np 1 --score-min L,0,-0.1 -p 16 -k 200. Read counting was then performed using the RNA-Seq by Expectation Maximization (RSEM, v1.3.1) [102] with the following settings: -p 8 --forward-prob 0 --paired-end. Normalization and differential gene expression analyses were performed using DESeq2 (v1.36.0) [103]. Genes with low expression (total read counts below 10) were removed and, treatment and control samples from the same replicate were paired for differential expression analysis. The significant differentially expressed genes (DEGs) were defined using a threshold of *q*-value ≤ 0.05.

Affymetrix raw data from [50] were processed as described in [6] using the GIANT v0.0.2 tools suite [104]. Normalized expression data, obtained using the apt-probeset-summarize tool of Affymetrix Power Tools (https://www.affymetrix.com/support/developer/powertools/changelog/index.html) using “gc correction+scale intensity+rma” as parameters, were used in gene set enrichment analyses.

### Pathway and gene-set enrichment analyses

Enrichment analyses for Gene Ontology Biological Processes (GOBPs), Gene Ontology Molecular Functions (GOMFs) and KEGG pathways were performed using Metascape (http://metascape.org) [105]. These analyses were performed on the top 500 significant (*q*-value < 0.05) up-regulated or down-regulated genes classified based on log_2_ fold-changes (log_2_ FC) issued from the RNA-seq analyses. Alternatively, genes assigned to regions with differential H3K27ac ChIP-seq signal were also used. GOBPs were further clustered using MonaGO (https://monago.erc.monash.edu/) [106] based on the average Resnik similarity between terms using 1.5 as cutoff [107].

Pre-ranked Gene-Set Enrichment Analyses (GSEA) were performed using the GSEA software (v3.0) [108] with the following settings: 1000 permutations, “weighted” as enrichment statistic and log_2_ FC as metric for ranking genes. The following gene sets were used : 61 liver fibrosis-associated genes of human MASH patient livers and in vitro and in vivo mouse models for liver fibrosis [47]; 163 genes defining a fibrogenic MF-HSC program of in vivo mouse models for liver fibrosis and reversal [45] and 71 liver fibrosis matrisome genes coding ECM proteins of human fibrotic livers (http://matrisomedb.pepchem.org/ [46]) (Supplementary Table 3). For the murine HSCs data, human ortholog genes were first retrieved using gProfiler (https://biit.cs.ut.ee/gprofiler/orth) [109]. In addition to enrichment plots, the normalized enrichment score (NES) and the FDR were also retrieved and displayed in the figures.

### HSC gene expression specificity indexes

Normalized relative log expression (RLE) data (CAGE-seq) from 126 human differentiated primary cells in basal condition were downloaded from the FANTOM5 website (https://fantom.gsc.riken.jp/5/sstar) [110] and used to define HSC gene expression specificity indexes. Specificity indexes correspond to log_2_ FC of average gene expression between MF-HSCs and other cell types.

### Statistical analyses

Statistical analyses were performed using the GraphPad Prism software (v9.4.1, GraphPad). Specific tests and corrections for multiple testing that were used are indicated in the figure legends. Statistical significance was displayed as follow *q < 0.05, **q < 0.01 and ***q < 0.001. Moreover, green and red colors were used to indicate a significant increase or decrease, respectively, while gray color was used to indicate no statistically significant changes.

### Supplementary information

Supplementary Figure Legends

Supplementary Figures

Uncropped images

Supplementary Table 1

Supplementary Table 2

Supplementary Table 3

Supplementary Table 4

Supplementary Table 5

Supplementary Table 6

Supplementary Table 7

## Data Availability

All RNA-seq and ChIP-seq data generated in this study have been deposited into the Gene Expression Omnibus (GEO) database under accession number GSE243107. The mass spectrometry proteomics data have been deposited to the ProteomeXchange Consortium via the PRIDE partner repository with the dataset identifier PXD045860 for the characterization of the LX-2 *O*-GlcNAcome and the identification of BNC2 and TEAD4 *O*-GlcNAcylated amino-acid residues.
