# Supplementary material for: O-GlcNAcylation controls pro-fibrotic transcriptional regulatory signaling in myofibroblasts
Source: Cell Death Dis. 2024 Jun 3;15(6):391. doi: 10.1038/s41419-024-06773-9 (PMC11148087; doi:10.1038/s41419-024-06773-9)
Supplement: Supplementary file 1 — Supplementary Figure Legends [file 41419_2024_6773_MOESM1_ESM.docx]

**Supplementary Figure 1. Additional characterization of myofibroblastic HSC activation models – related to Fig. 1**

**A.** Images obtained as described in **Fig. 1A** using livers slices from additional human donors (each line shows data for an individual donor). White squares indicate regions for which a zoom image is provided on the right. Scale bars=5,000 µm for images showing entire sections and 500 µm for zoom images.

**B.** RT-qPCR data showing *ANKRD1* expression in LX-2 cells grown as described in **Fig. 1F** (*n=*4 biologically independent experiments). Log_2_ fold changes (log_2_ FC) between 3D and 2D cells (left panel), and between 3D🡪2D and 3D (right panel) are shown.

**C.** LX‑2 cells were serum starved for 24 h and treated or not with 1 ng/mL TGF-β for 24 h. Western blot assays and their quantifications of *O*‑GlcNAcylation, OGT and ACTA2 protein levels are shown. GAPDH was used as protein loading control. The presented images are representative of at least 3 biologically independent experiments. MW, molecular weight markers. Log_2_ FC between TGF-β and control conditions are shown.

For **all panels**, the bar graphs show means + *SD* together with individual biological replicates. Two-sided one-sample t-test with Benjamini‑Hochberg correction for multiple testing was used to determine if the mean log_2_ FC was statistically different from 0.

**Supplementary Figure 2. Additional data supporting a specific effect of OGT inhibition on isolated MF-HSCs – related to Fig. 2**

**A.** LX‑2 cells were starved in glucose (Glc)-deprived medium for 24 h and then exposed to medium with or without 5 mM glucosamine (GlcNH_2_) or 11 mM Glc for 24 h. Western blot assays (left panel) and their quantifications (middle panel) of *O*‑GlcNAcylation and COL1A1 protein levels are shown. GAPDH was used as protein loading control. The presented images are representative of 4 biologically independent experiments. MW, molecular weight markers. All samples were loaded onto the same western blot with intermediate non-relevant conditions being cropped out. RT-qPCR data showing gene expression levels (right panel, *n=*4 biologically independent experiments). Log_2_ fold changes (log_2_ FC) between GlcNH_2_ or Glc and control (starved) conditions are shown.

**B.** LX-2 cells were treated or not with 5, 25, 50 or 100 µM of the OGT inhibitor OSMI-1 (referred to as OGTi) for 24 h. Western blot assays (left panel) and their quantifications (middle panel) of *O*‑GlcNAcylation and COL1A1 protein levels are shown. GAPDH was used as protein loading control. The presented images are representative of 4 biologically independent experiments. MW, molecular weight markers. RT‑qPCR data showing gene expression (right panel, *n=*4 biologically independent experiments). Log_2_ FC between OGTi and control (non‑treated) conditions are shown. ns, not significant.

**C.** LX-2 cells were treated as in **panel B** and cell viability was measured with the Cytotox 96® Non-Radioactive Cytotoxicity Assay kit (*n=*3 biologically independent experiments).

**D.** LX-2 cells were treated as in **panel B** and number of dead (trypan blue-positive) cells was counted (*n=*3 biologically independent experiments).

**E.** LX-2 cells were treated or not with 50 µM ST045849 for 24 h**.** Western blot assays (left panel) and their quantifications (middle panel) of *O*-GlcNAcylation and COL1A1 protein levels are shown. GAPDH was used as protein loading control. The presented images are representative of 4 biologically independent experiments. MW, molecular weight markers. RT‑qPCR data showing gene expression (right panel, *n=*4 biologically independent experiments). Log_2_ FC between ST045849 and control (non-treated) conditions are shown.

**F-G.** Mouse pQ-HSCs (1 d of culture) or pMF-HSCs (7 d of culture) were respectively treated or not with 50 µM OGTi for 7 d or 24 h (*n=*3 biologically independent experiments). Cellular metabolic activity of pMF-HSC not treated or treated with OGTi for 24 h or 7 d was measured with the TACS MTT Cell Proliferation Assay kit (**panel F**) and cell viability was measured with the Cytotox 96® Non‑Radioactive Cytotoxicity Assay kit (**panel G**). The effect of OGTi on cellular metabolic activity rather than on cell viability suggests quiescent, slow-growing cells with low metabolic rates.

For **all panels**, the bar graphs show means + *SD* together with individual biological replicates. Two-sided one-sample t-test with Benjamini‑Hochberg correction for multiple testing was used to determine if the mean log_2_ FC was statistically different from 0 in **panels A and E**, and one‑way ANOVA with Dunnett multiple comparison post-hoc test was used in **panels B, C, D, F and G**.

**Supplementary Figure 3. Expression of functional hepatocyte and MF-HSC marker genes in mouse models of liver injury – related to Fig. 3**

**A-B.** Mouse PCLS were cultured for 6 d to induce fibrosis and then subjected or not to OGTi (50 µM OSMI-1) for 24 h**.**

**A.** RT-qPCR data showing collagen encoding gene expression changes between mouse PCLS at d0 and at d7 non‑treated for the last 24 h (d7 - Control) (*n=*6 PCLS from independent mice). Fold changes (FC) relative to d0 (arbitrarily set to 1) are shown. Batch effect was removed by setting the mean of d0 conditions for each series of experiments, *i.e.* performed at different times, to 1. One‑tailed Mann-Whitney *U* test with Benjamini‑Hochberg correction for multiple testing was used.

**B.** RT-qPCR data showing functional hepatocyte markers (*Cyp2e1* and *Albumin* (*Alb*)) gene expression changes between mouse PCLS at d0, at d7 non‑treated (d7 - Control) or treated with 50 µM OGTi (OSMI-1) for the last 24 h (d7 - OGTi) (*n=*6 PCLS from independent mice). FC relative to d7 - Control (arbitrarily set to 1) are shown. Batch effect was removed by setting the mean of d7 - Control conditions for each series of experiments, *i.e.* performed at different times, to 1. One‑way ANOVA with Tukey multiple comparison post-hoc test was used.

**C.** Plasma ALT and AST activity levels in control (*n=*6 mice), CCl_4_ (*n=*8 mice) and CCl_4_ + OGTi mice (*n=*8 mice). One-way ANOVA with Tukey multiple comparison post‑hoc test was used to assess the statistical significance of differences between the CCl_4_ + OGTi, CCl_4_ and control group. ns, not significant.

**D.** RT‑qPCR data showing *Cyp2e1* (left graph) and *Alb* (right graph) expression in livers of control (*n=*6 mice), CCl_4_ (*n=*8 mice) and CCl_4_ + OGTi (*n=*8 mice) treated mice. FC relative to control mice (arbitrarily set to 1) are shown. One-way ANOVA with Tukey multiple comparison post‑hoc test was used. ns, not significant.

**E.** RT‑qPCR data showing indicated gene expression changes in livers of control (*n=*6 mice) and CCl_4_-treated mice (*n=*8 mice). FC between mice of the CCl_4_ and control (arbitrarily set to 1) group are shown. One‑tailed Mann-Whitney *U* test with Benjamini‑Hochberg correction for multiple testing was used.

**F.** Normalized collagen encoding gene expression data issued from RNA-seq data obtained from purified HSCs, hepatocytes (HCs), cholangiocytes (CHs), Kupffer cells (KCs) and liver sinusoidal endothelial cells (LSECs) from livers of mice treated with 0.5 ml/kg CCl_4_ for 24 h (*n=*3) (manuscript in preparation). One-way ANOVA with Dunnett multiple comparison post‑hoc test was used.

**G.** Sirius red staining was performed to visualize the deposition of collagen in non-treated PCLS at d0 and at d9 (d9 - Control) (shown images are representative of *n=*5 PCLS from independent mice). Scale bars=2,000 µm for images showing entire sections and 200 µm for zoom image. Log_2_ FC of Sirius red positive areas between PCLS at d9 and d0 are shown. Two‑sided one-sample t-test was used to determine if the mean log_2_ FC was statistically different from 0.

**H-I.** RT-qPCR data showing collagen (panel **H**) and functional hepatocyte markers (panel **I**) gene expression changes between mouse PCLS at d0, d9 non-treated (d9 - Control) or PCLS treated with 50 µM OGTi throughout 9 d (d9 - OGTi) (*n=*4 PCLS from independent mice). FC relative to d0 (arbitrarily set to 1 for panel **H**) or d9 - Control (arbitrarily set to 1 for panel **I**) are shown. Batch effect was removed by setting the mean of d0 (panel **H**) or d9 - Control (panel **I**) conditions for each series of experiments, *i.e.* performed at different times, to 1. One‑way ANOVA with Tukey multiple comparison post-hoc test was used.

For **all panels**, the graphs show means + *SD* together with individual biological replicates or mice.

**Supplementary Figure 4. Additional characterization of the effects of OGTi and siOGT on the MF transcriptomic program – related to Fig. 4**

**A.** Common GOBP and KEGG Pathways obtained when mining the top 500 upregulated genes upon treatment with OGTi (OSMI-1) or siOGT (see **Materials and Methods**) using Metascape and MonaGO.

**B.** Detailed enrichment plots from GSEA corresponding to data displayed in **Fig. 4D**. NES and FDR are respectively the normalized enrichment score and the false discovery rate provided by GSEA.

**C.** Human primary cardiac myofibroblasts (7 d of culture) were treated or not with 50 µM OGTi for 24 h (*n=*4 from independent donors). RT‑qPCR data show indicated collagen encoding gene expression. Log_2_ FC between OGTi and control (non-treated) conditions are shown.

**D.** LL29 cells were treated or not with 50 µM OGTi for 24 h. Western blot assays (left panel) and their quantifications (middle panel) for total protein *O*‑GlcNAcylation and COL1A1 levels are shown. GAPDH was used as protein loading control. The presented images are representative of 3 biologically independent experiments. MW, molecular weight markers. RT‑qPCR data showing indicated collagen encoding gene expression are also displayed (right panel, *n=*4 biologically independent experiments). Log_2_ FC between OGTi and control (non‑treated) conditions are shown.

**Supplementary Figure 5. OGT inhibition by ST045849 also inhibits myofibroblastic activities of MF-HSCs – related to Fig. 5**

**A.** LX-2 cells were treated or not with 50 µM ST0454849 for 24 h and secreted COL1A1 was measured in the supernatant by ELISA (*n=*4 biologically independent experiments). Secreted COL1A1 is shown relative to that in the control (non-treated) condition arbitrarily set to 100. Two-sided one-sample t‑test was used to determine if the mean log_2_ fold changes (log_2_ FC) between ST045849 and control conditions were statistically different from 0.

**B.** LX-2 cells were treated as in **panel A** and Hoechst 33258 staining of adherent cells was performed to assess binding to collagen-coated matrix (shown images are representative of 3 biologically independent experiments). EDTA treatment was used a control of signal specificity. Scale bars=50 µm. Number of adherent cells is shown relative to that in the control (non-treated) condition arbitrarily set to 100. Two-sided one-sample t-test was used to determine if the mean log_2_ FC between ST045849 and control conditions was statistically different from 0.

For **all panels**, the bar graphs show means + *SD* together with individual biological replicates.

**Supplementary Figure 6. Additional characterization of *O*-GlcNAcylation-dependent transcriptional regulatory regions in MF-HSCs – related to Fig. 6**

**A.** Average H3K27ac ChIP-seq (*n=*4 biologically independent experiments) and CoP-seq signals from LX-2 cells treated or not with OGTi (50 µM OSMI‑1) for 24 h. Heatmaps show the signals in ± 2.5 kb windows around the center of regions identified as displaying a significant gain (*n*=3,623) or no change (*n*=37,338) in H3K27ac in OGTi-treated cells.

**B.** Enrichment plot from GSEA performed using genes associated with H3K27ac loss as the gene set and the RNA-seq data of LX-2 cells treated with OGTi. NES and FDR are respectively the normalized enrichment score and the false discovery rate provided by GSEA.

**C.** Genes assigned to regions losing H3K27ac (from **Fig. 6C**) were mined for enriched pathways (GO Biological Process (GOBP) and KEGG Pathways) previously highlighted in the transcriptomic analysis (**Fig. 4C-D**) using Metascape. Enriched GOBP terms were clustered according to the Resnik similarity score using MonaGO. White color indicates a lack of significance.

**D.** The top 5 *de novo* motifs (left) and the top 5 TF DNA recognition motifs from the JASPAR CORE 2020 database (right) enriched in regions losing H3K27ac identified respectively by RSAT ^99^ and TFmotifView ^98^. NR stands for nuclear receptor.

**E.** The TEAD4 cistrome in LX-2 cells (21,113 binding sites) was intersected with H3K27ac regions with gain, loss or unchanged signal upon OGTi treatment previously identified. Percentage of each set of H3K27ac regions bound by TEAD4 is shown. Statistical significance was assessed using Fisher’s exact test (only comparison between H3K27ac gained and lost regions is shown to avoid biased with large differences in sample sizes when using unchanged H3K27ac regions).

**F.** Nuclear extracts from LX-2 cells were subjected to immunoprecipitation with antibodies against YAP1 (D8H1X, Cell Signaling; NB110-58358, Novus Biologicals). Input and immunoprecipitated materials were analyzed by simple western immunoassay using antibodies directed against BNC2, TEAD4 and YAP1.

**Supplementary Figure 7. *O*-GlcNAcylation targets key MF TRs**

The identification of *O*-GlcNAcylation on BNC2, TEAD4 and YAP1 was defined as described in the **Materials and Methods** section using nuclear protein fractions from LX-2 cells. Input and enriched materials were analyzed by western blot and simple western immunoassay using streptavidin‑HRP and antibody directed against BNC2, TEAD4 and YAP1.

**Supplementary Figure 8. *O*-GlcNAcylation does not regulate TEAD1 protein levels and chromatin binding**

**A.** Simple western immunoassays (left panel) and their quantifications (right panel) of TEAD1 in LX-2 cells treated as described in **Fig. 7A** (*n=*4 biologically independent experiments). HSP90 was used as protein loading control. MW, molecular weight markers.

**B.** Western blot assays and simple western immunoassays (left panel), and their quantifications (right panel) of chromatin-bound TEAD1 in LX-2 cells treated as described in **Fig. 7E** (*n=*4 biologically independent experiments). HSP90 and LMNA were used as protein loading controls. The presented images are representative of at least 4 biologically independent experiments. MW, molecular weight markers.

For **all panels**, the bar graphs show means + *SD* together with individual biological replicates.

Two-sided one-sample t-test was used to determine if the mean log_2_ fold changes (log_2_ FC) between OGTi and control conditions was statistically different from 0. ns, not significant.

**Supplementary Figure 9. HCD/MS/MS spectra of peptides from BNC2 or TEAD4 containing *O*-GlcNAcylated sites – related to Fig. 8**

**A.** HCD-MS/MS spectra depict containing *O*-GlcNAcylated sites at T455 on the peptide sequence TFYDK(acetylation)GT(HexNac)LK(acetylation) at *m/z* 453.8936 with *z*=+3 and S490 on the peptide sequence N(deamidation)RHS(HexNac)ANPNP(oxidation)RLHMP(oxidation)MLRNNRDK at *m/z* 968.8107 with *z*=+3 on BNC2 and, the site S69 on the peptide sequence K(acetylation)IILS(HexNac)DEGK(acetylation) at *m/z* 645.3474 with *z*=+2 and position S99 on the peptide sequence Q(deamidation)VS(HexNac)SHIQ(deamidation)VLAR at *m/z* 721.8746 with *z*=+2 on TEAD4, as described in **Fig. 8A**. The modified sites are highlighted in red within the peptide sequence.

**Supplementary Figure 10. Effect of TEAD4 overexpression on OGTi-mediated decrease in collagen‑encoding gene expression in MF-HSCs**

**A.** LX-2 cells were transfected with a control plasmid (mock) or Myc-TEAD4 plasmids for 24 h. Then, cells were treated or not with 50 µM OSMI-1 (referred to as OGTi) for an additional 24 h and used to prepare extracts analyzed using western blot and simple western immunoassays. TEAD4 levels were assessed and HSP90 was used as a protein loading control. The presented images are representative of 3 biologically independent experiments. MW, molecular weight markers. For TEAD4, the same blot is revealed following short and long exposure, as specified.

**B.** LX-2 cells were transfected as in **panel A** and used for RT-qPCR analyses of indicated gene expression (*n=*3 biologically independent experiments). The bar graphs show means + *SD* together with individual biological replicates. Log_2_ FC between OGTi and control conditions are shown. Unpaired t-test was used to assess the statistical significance between Myc-TEAD4 expressing cells and the control (mock) condition. ns, not significant.

**Supplementary Figure 11. *O*-GlcNAcylation is required for TGF-β pro-fibrogenic stimulation of MF-HSCs**

**A.** LX-2 cells were treated or not with 1 ng/mL TGF-β and/or 50 µM OSMI-1 (referred to as OGTi) for 24 h. Western blot assays and their quantifications of *O*‑GlcNAcylation and COL1A1 protein levels. GAPDH was used as protein loading control. The presented images are representative of 3 biologically independent experiments. MW, molecular weight markers. Fold changes (FC) between TGF-β, OGTi or TGF-β + OGTi and the control condition are shown. ns, not significant.

**B.** LX-2 cells were treated as in **panel A**. RT-qPCR data showing gene expression are displayed (*n=*3 biologically independent experiments). FC between TGF-β, OGTi or TGF-β + OGTi and the control condition are shown.

For **all panels**, the bar graphs show means + *SD* together with individual biological replicates.

Two-way ANOVA with Sidak multiple comparison post‑hoc test was used to assess the statistical significance of differences between the TGF-β and the control condition, or between the TGF-β + OGTi and OGTi conditions. ns, not significant.
