# Supplementary material for: O-GlcNAcylation controls pro-fibrotic transcriptional regulatory signaling in myofibroblasts
Source: Cell Death Dis. 2024 Jun 3;15(6):391. doi: 10.1038/s41419-024-06773-9 (PMC11148087; doi:10.1038/s41419-024-06773-9)

Supp Figure 1

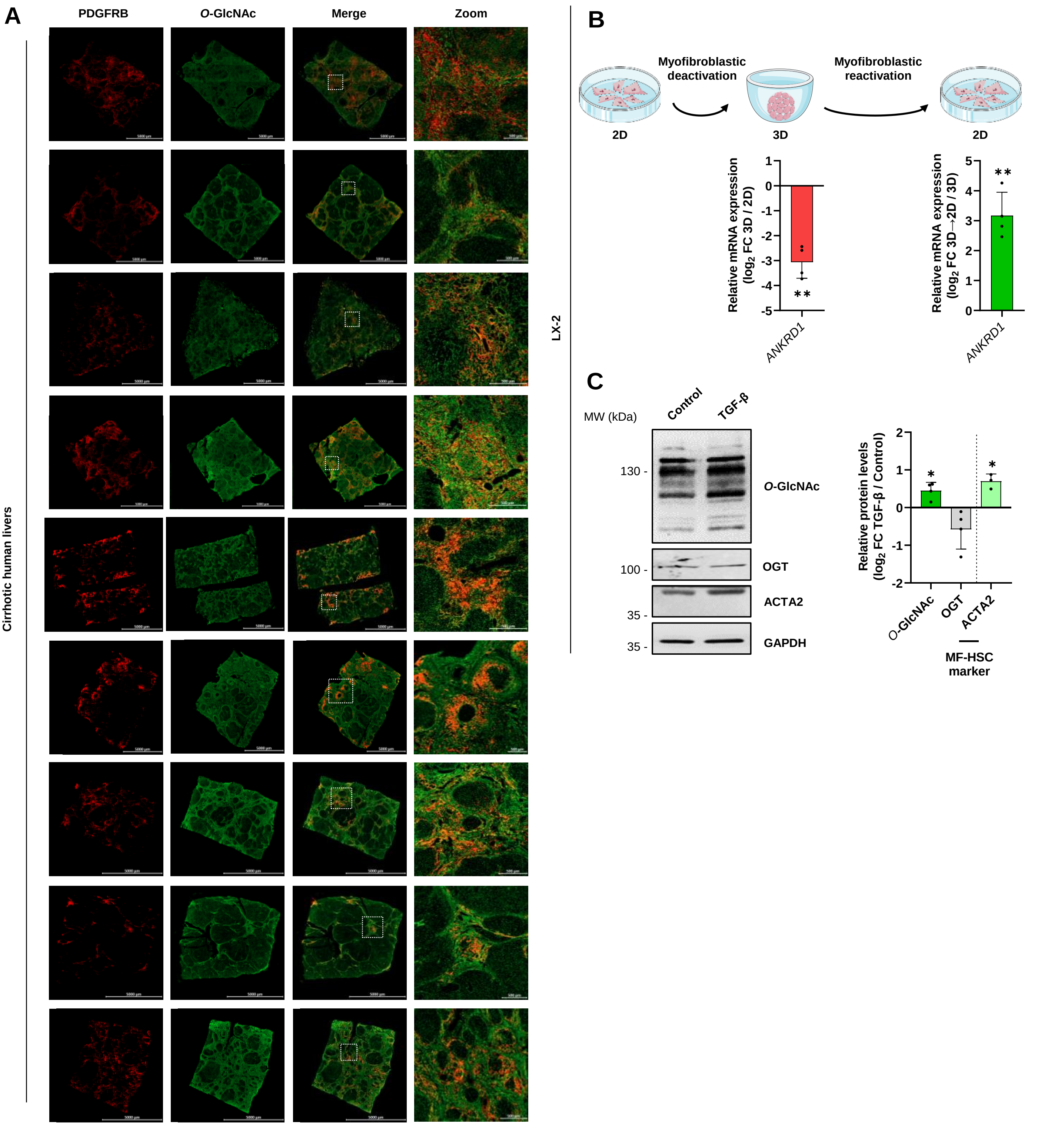

Supp Figure 2

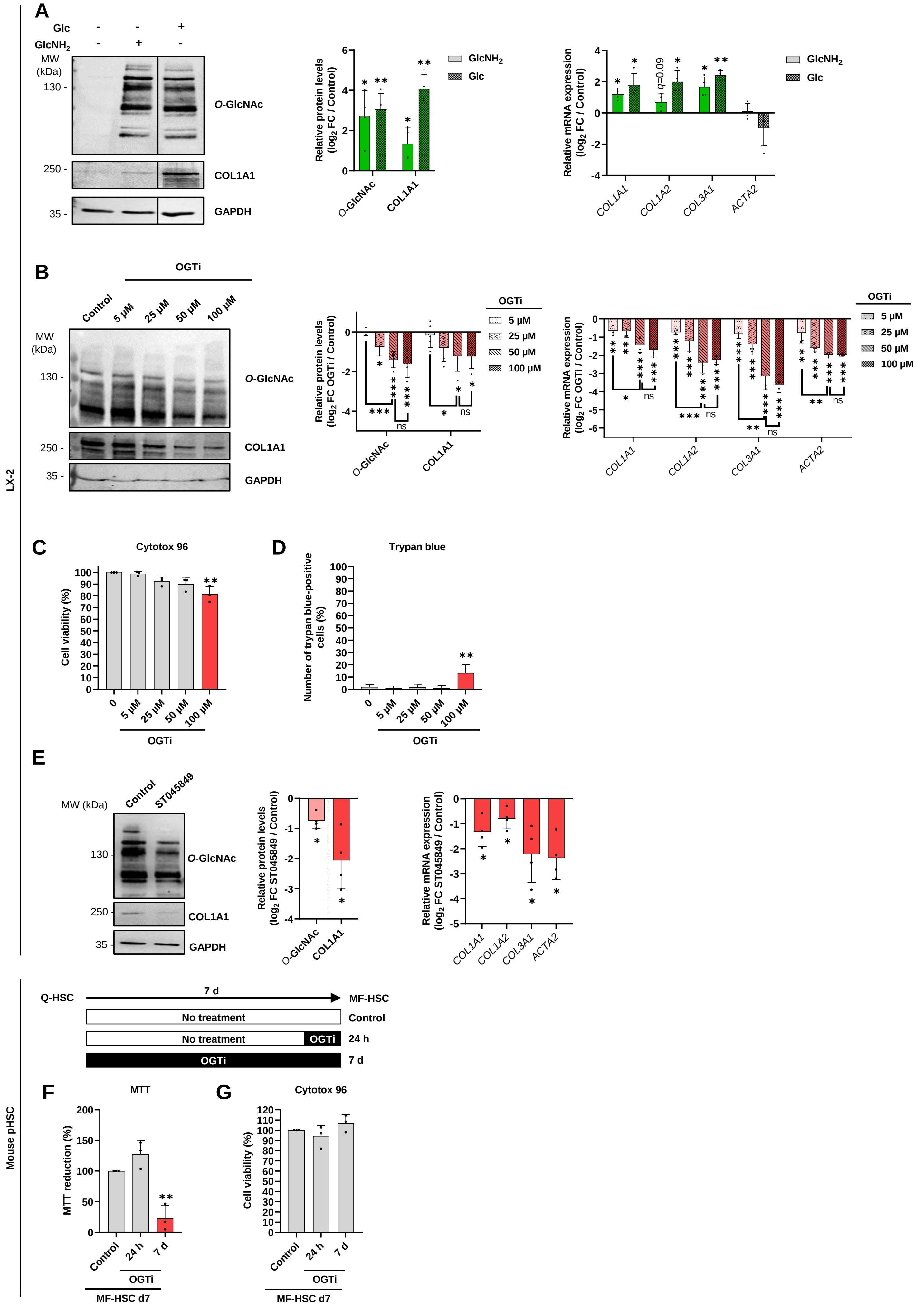

Supp Figure 3

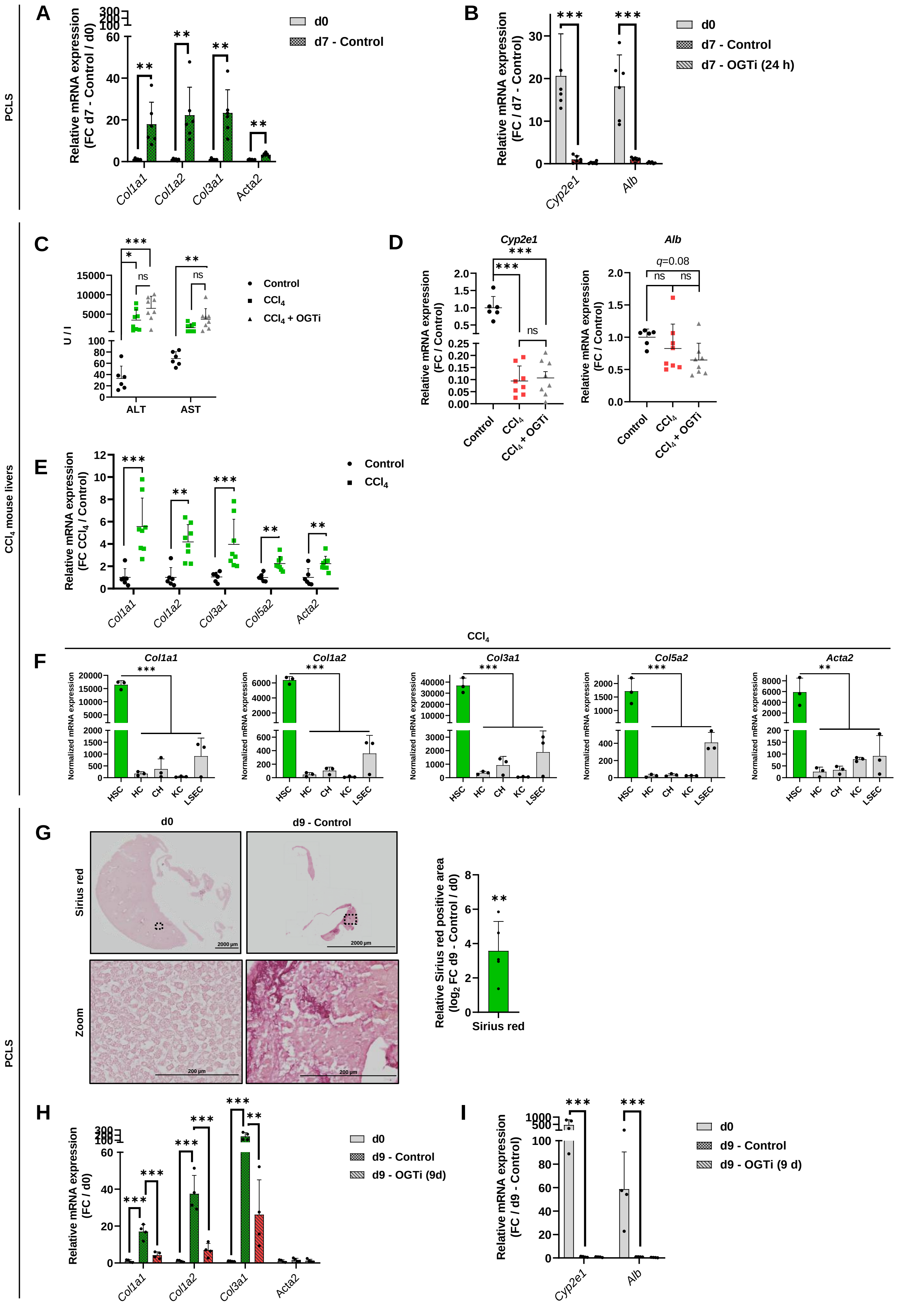

Supp Figure 4

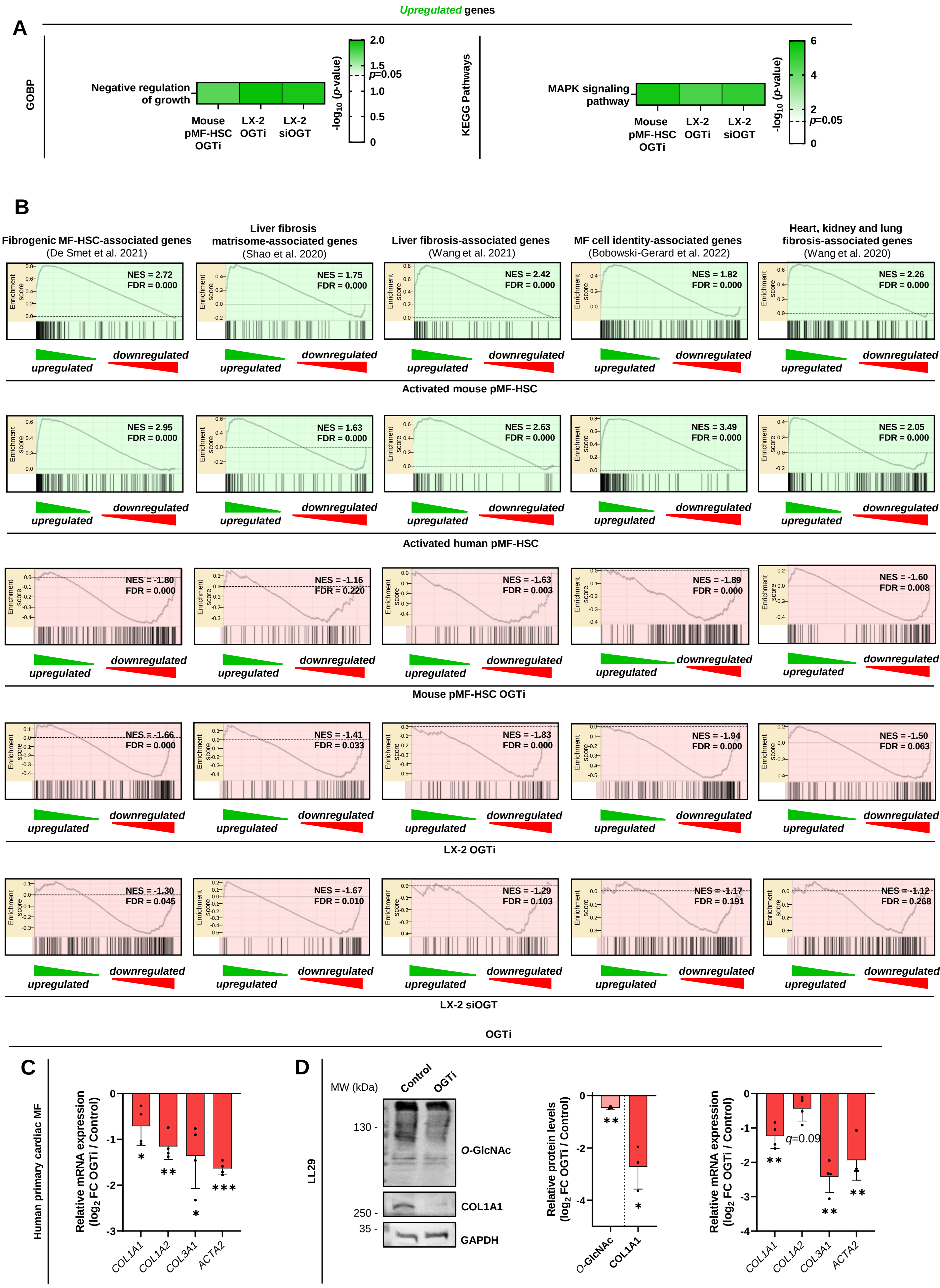

Supp Figure 5

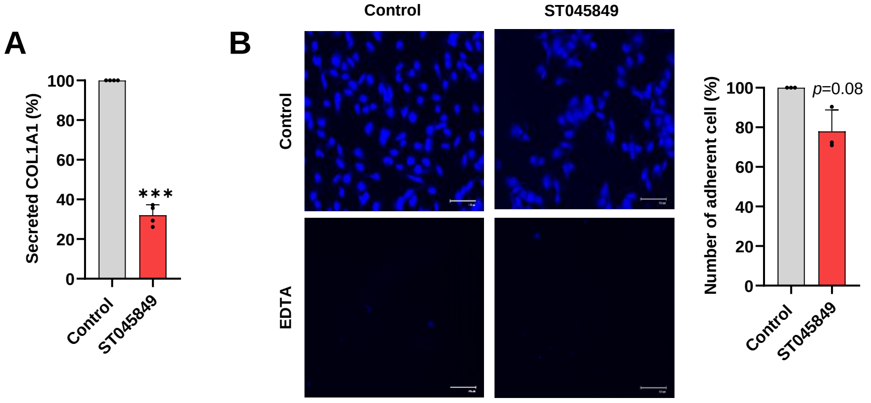

Supp Figure 6

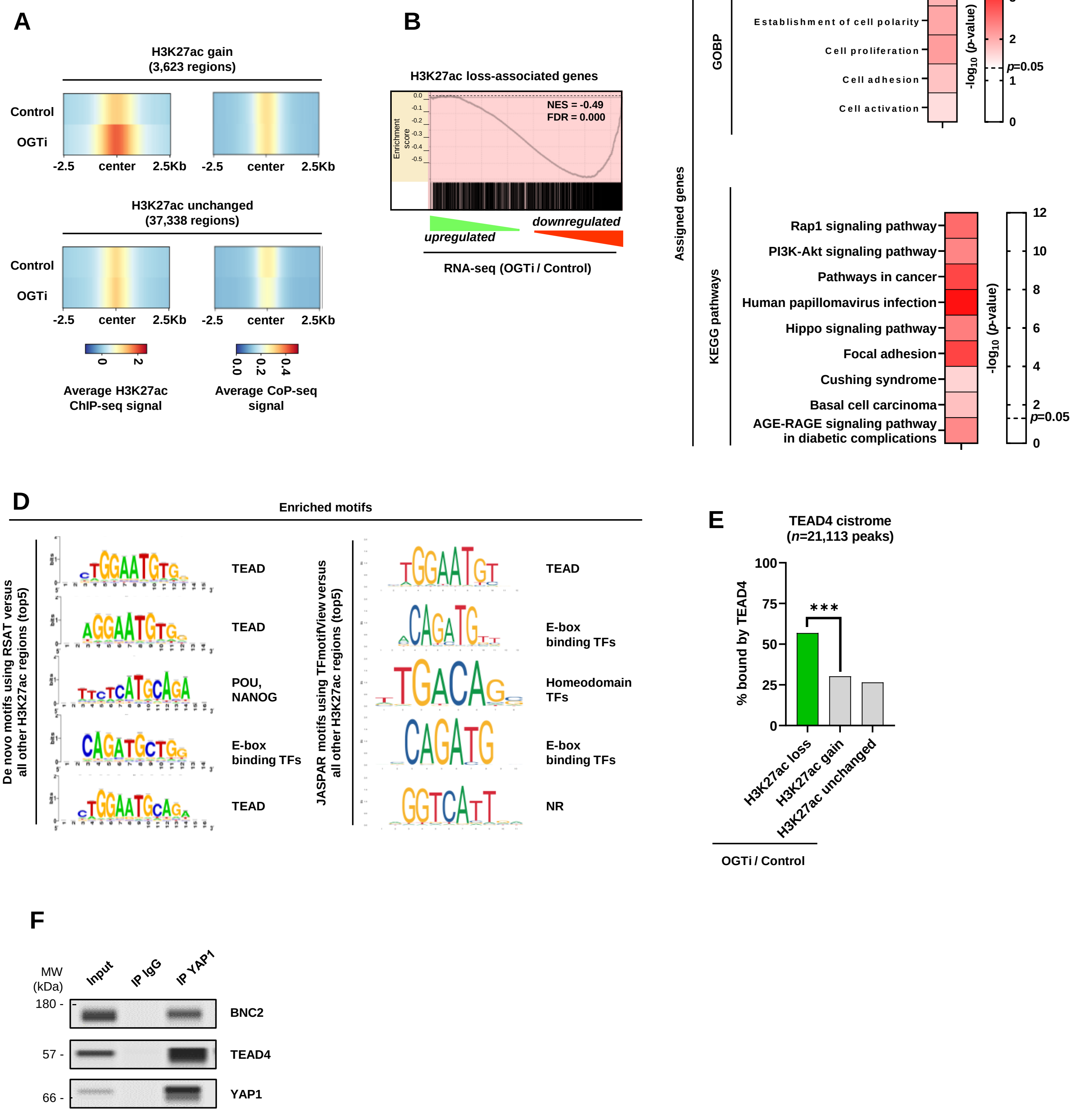

Supp Figure 7

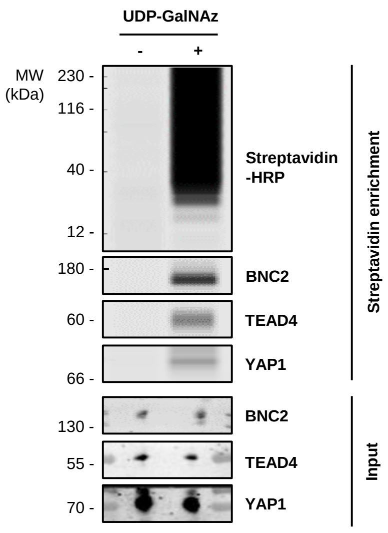

Supp Figure 8

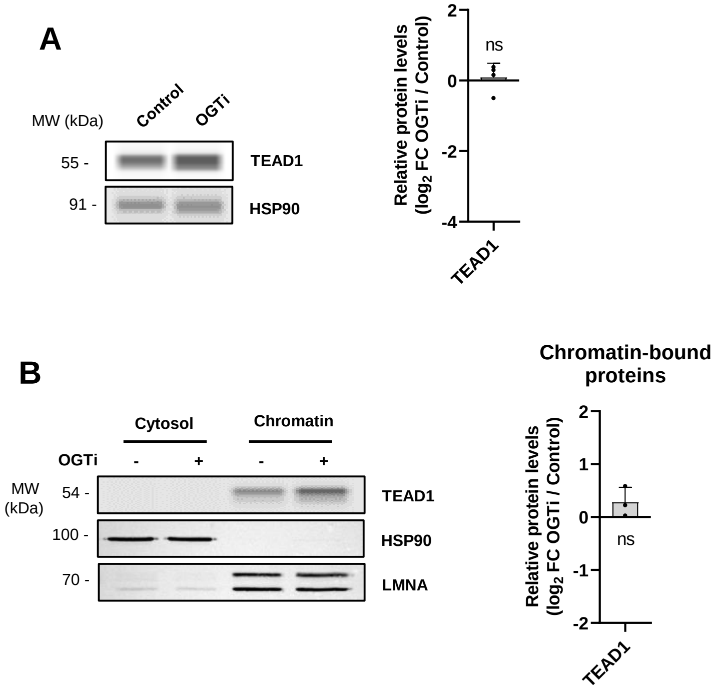

Supp Figure 9

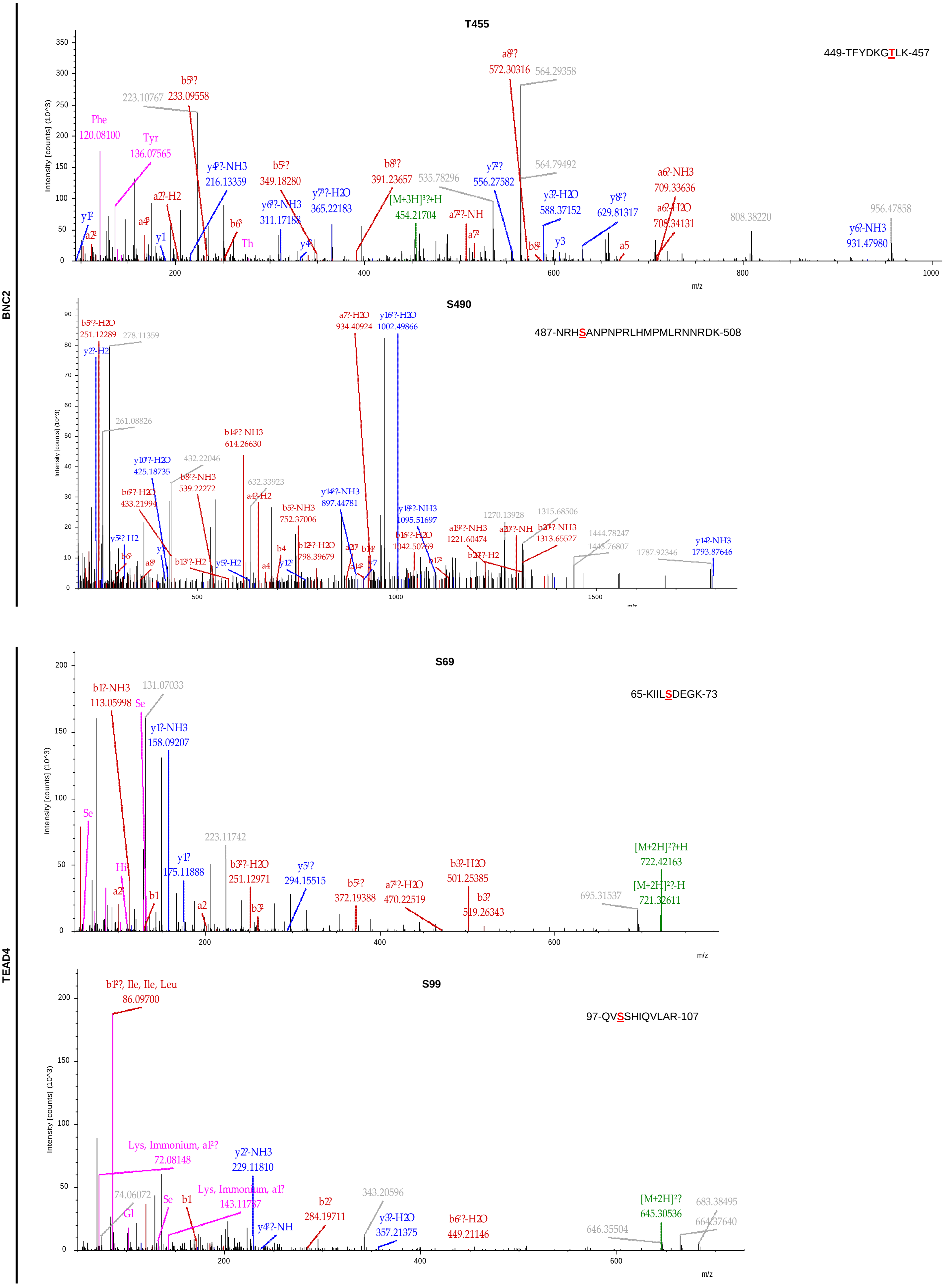

Supp Figure 10

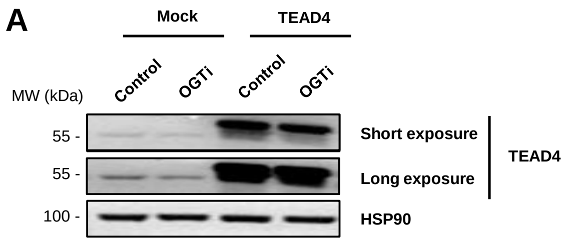

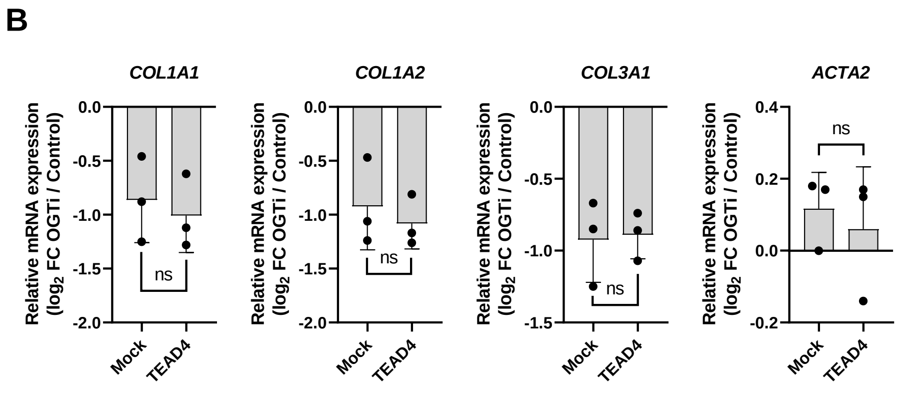

Supp Figure 11

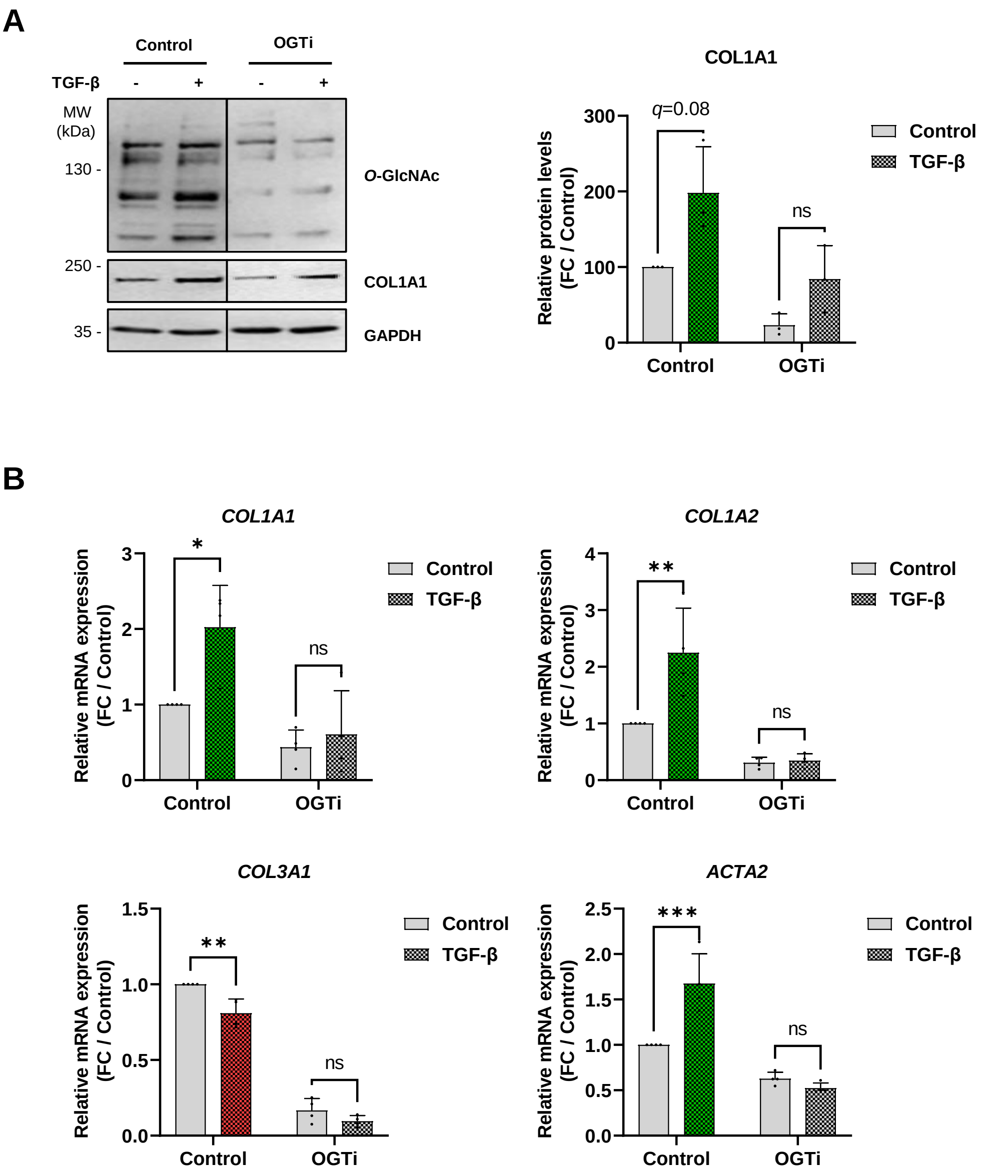

Supplement: Supplementary file 2 — Supplementary Figures [file 41419_2024_6773_MOESM2_ESM.pdf]
