# Supplementary material for: O-GlcNAcylation controls pro-fibrotic transcriptional regulatory signaling in myofibroblasts
Source: Cell Death Dis. 2024 Jun 3;15(6):391. doi: 10.1038/s41419-024-06773-9 (PMC11148087; doi:10.1038/s41419-024-06773-9)

Figure 1C

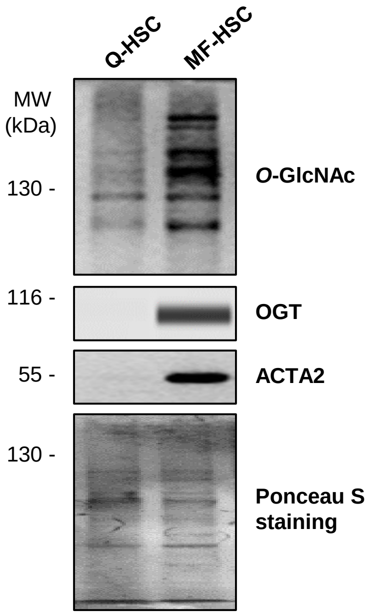

Q-HSC
MF-HSC
MW (kDa)
130 -
O-GlcNAc
116 -
OGT
55 -
ACTA2
130 -
Ponceau S staining

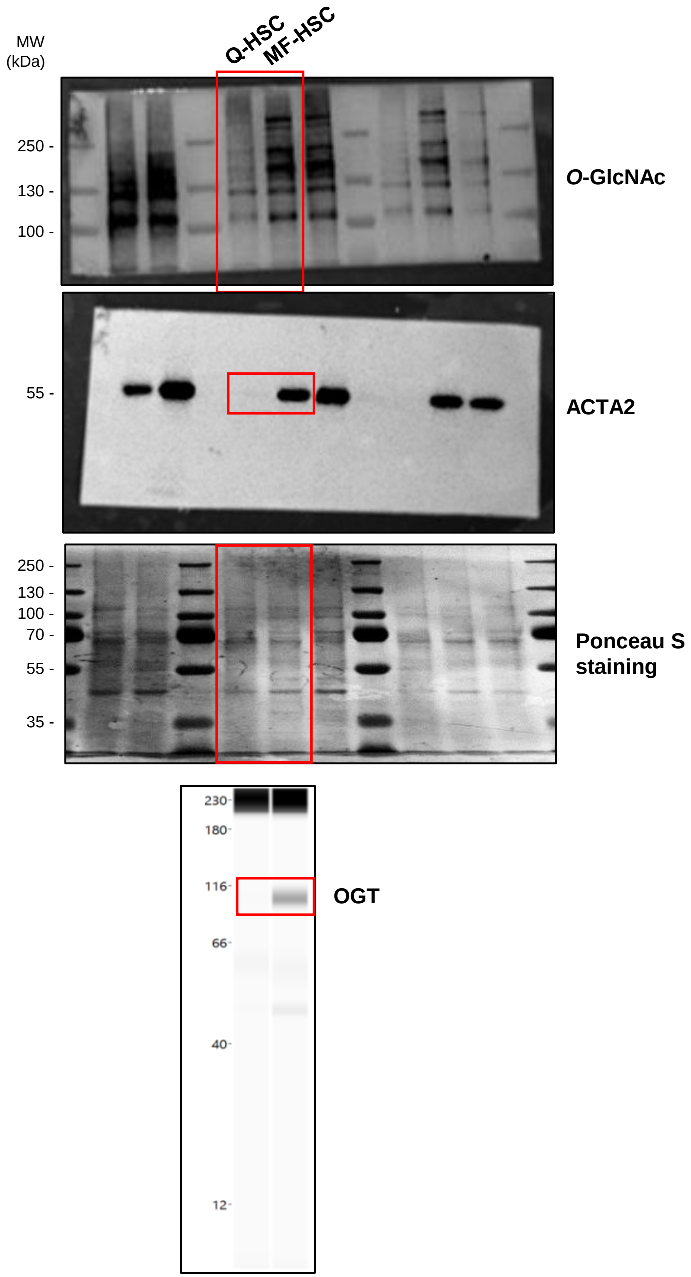

MW (kDa)
Q-HSC
MF-HSC
250 -
130 -
100 -
O-GlcNAc
55 -
ACTA2
250 -
130 -
100 -
70 -
55 -
35 -
Ponceau S staining
230
180
116
66
40
12
OGT

Figure 1F

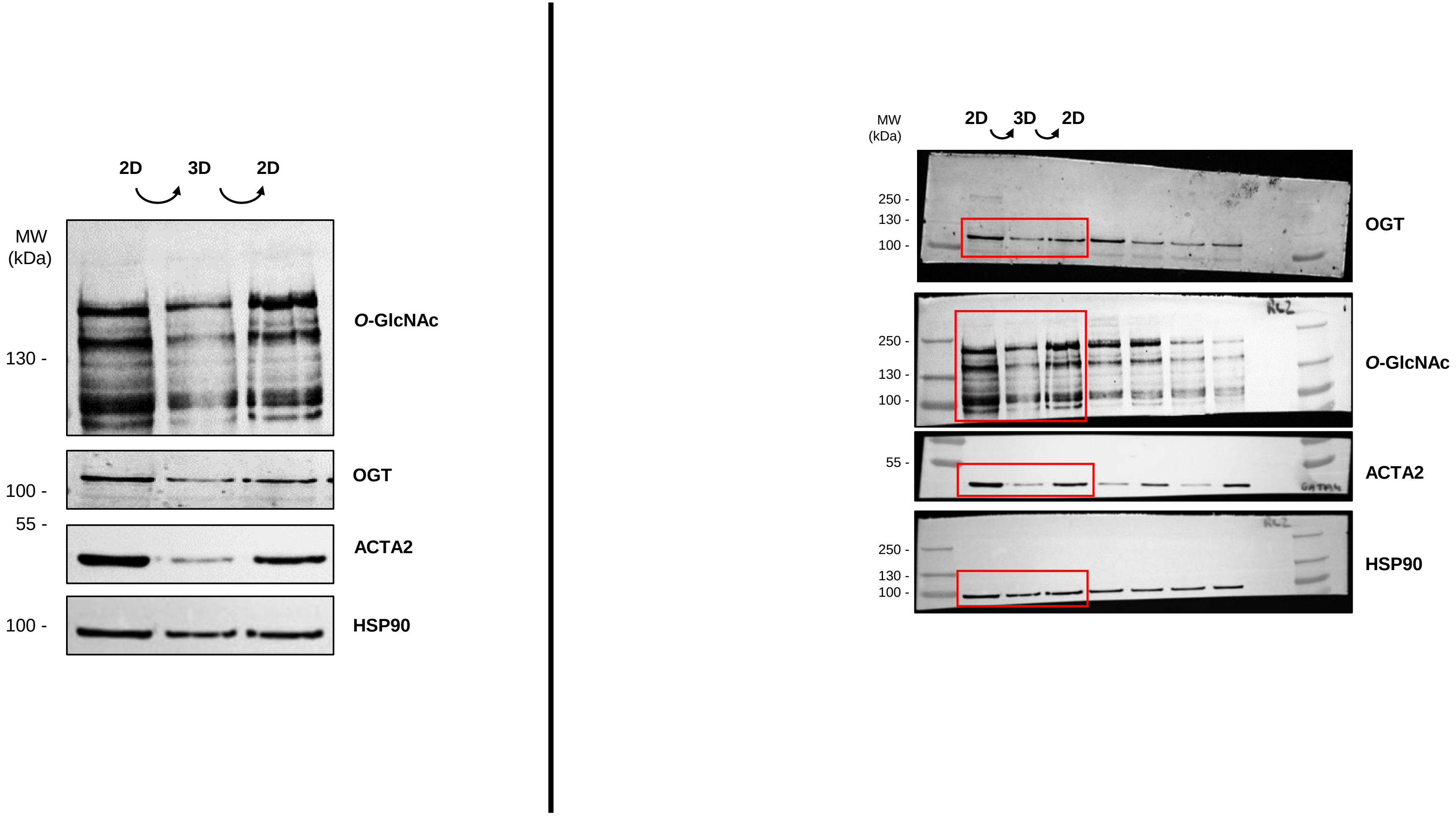
2D
3D
2D
MW
(kDa)
130 -
O-GlcNAc
100 -
OGT
55 -
ACTA2
100 -
HSP90
MW
(kDa)
2D
3D
2D
250 -
130 -
100 -
OGT
250 -
130 -
100 -
O-GlcNAc
55 -
ACTA2
250 -
130 -
100 -
HSP90

Supplementary Figure 1C

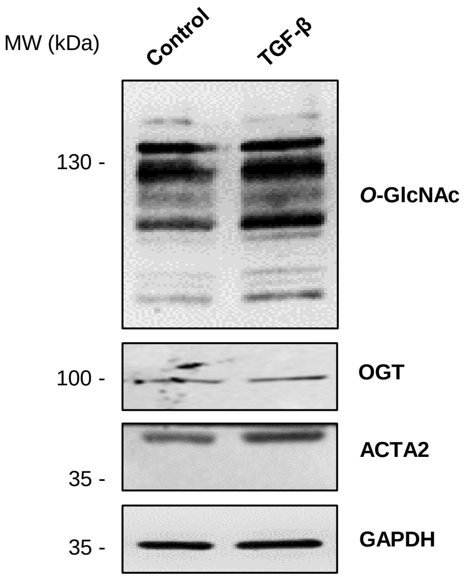

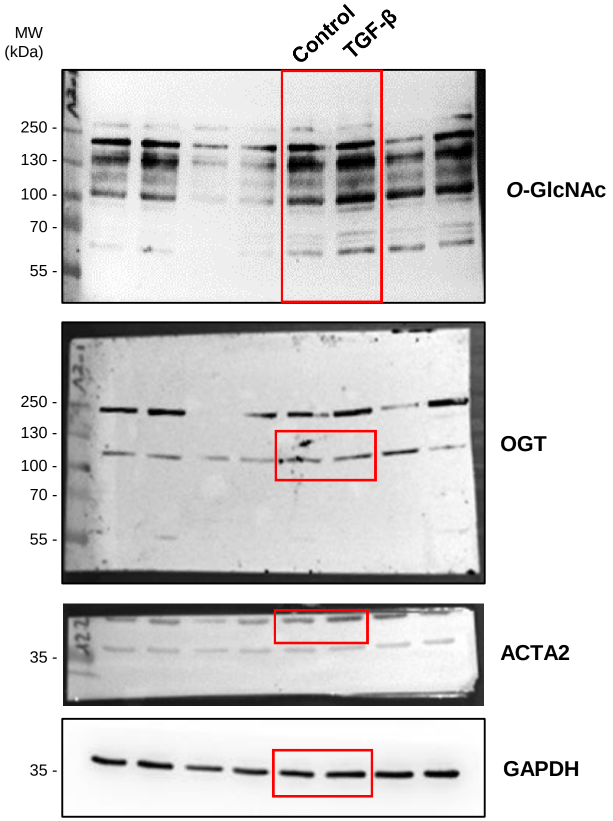

Figure 2A

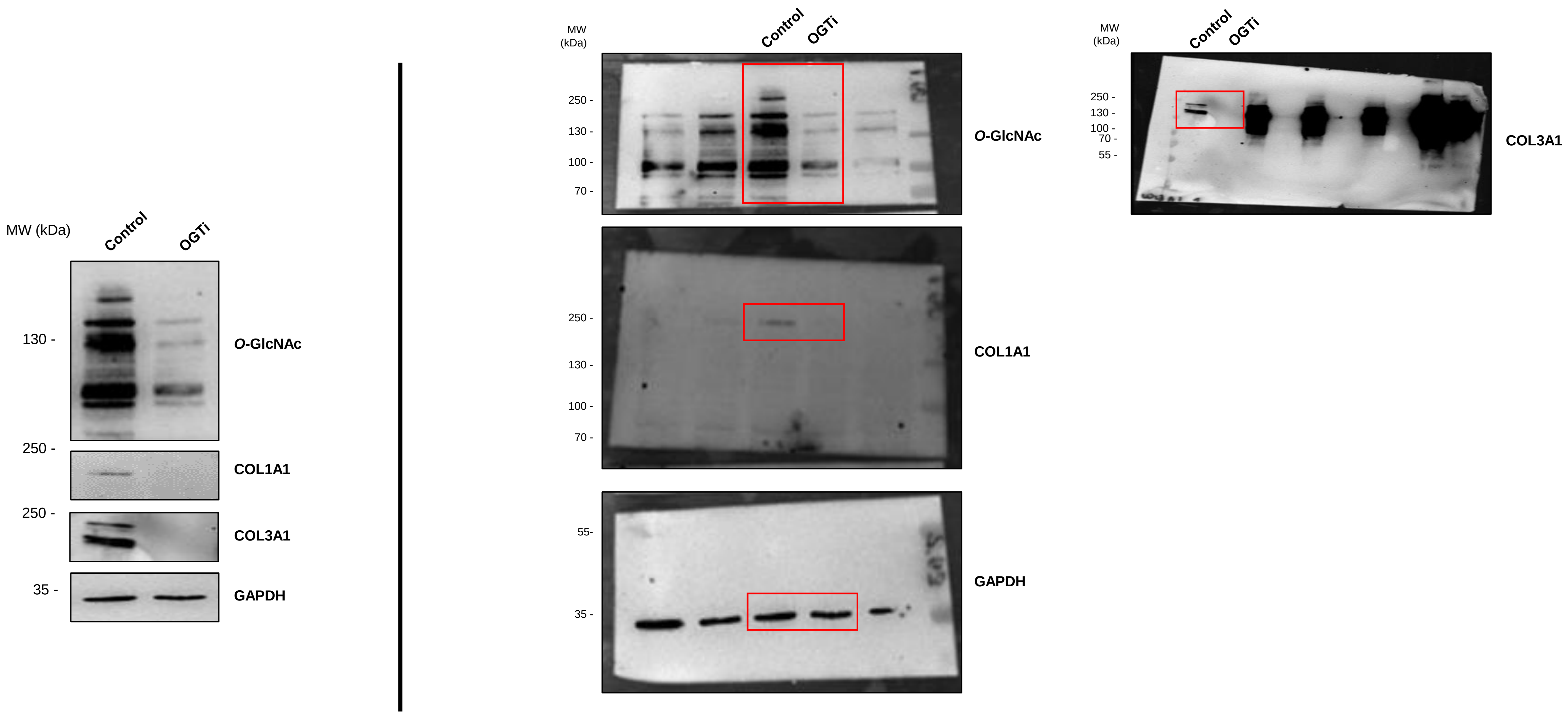
MW (kDa)
Control
OGTi
130 -
O-GlcNAc
250 -
COL1A1
250 -
COL3A1
35 -
GAPDH
MW (kDa)
Control
OGTi
250 -
130 -
100 -
70 -
O-GlcNAc
250 -
130 -
100 -
70 -
COL1A1
55-
35 -
GAPDH
MW (kDa)
Control
OGTi
250 -
130 -
100 -
70 -
55 -
COL3A1

Figure 2B

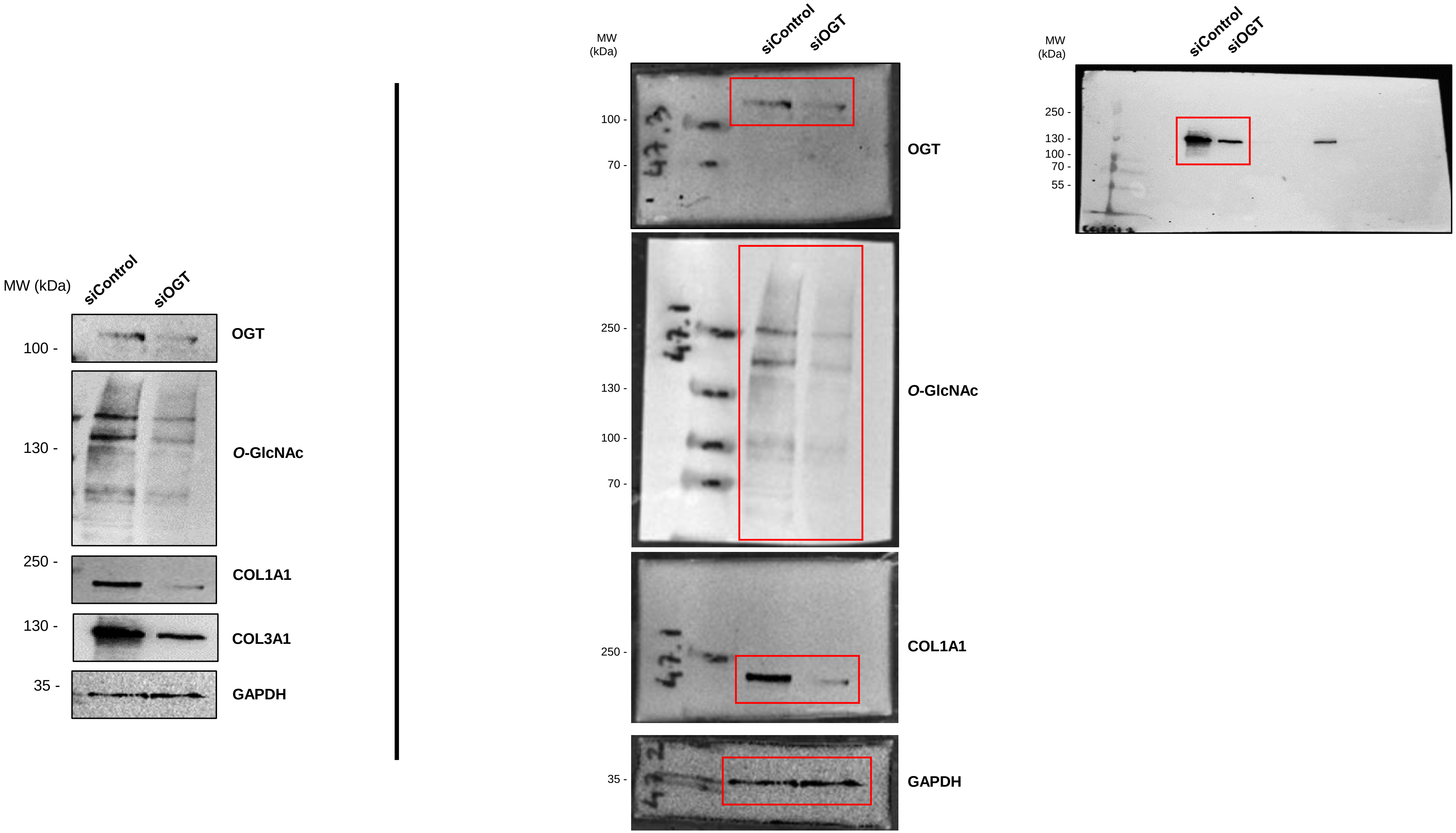
MW (kDa)
siControl
siOGT
100 -
130 -
250 -
130 -
35 -
OGT
O-GlcNAc
COL1A1
COL3A1
GAPDH
MW (kDa)
siControl
siOGT
100 -
70 -
OGT
250 -
130 -
100 -
70 -
O-GlcNAc
250 -
COL1A1
35 -
GAPDH
MW (kDa)
siControl
siOGT
250 -
130 -
100 -
70 -
55 -

Supplementary Figure 2A

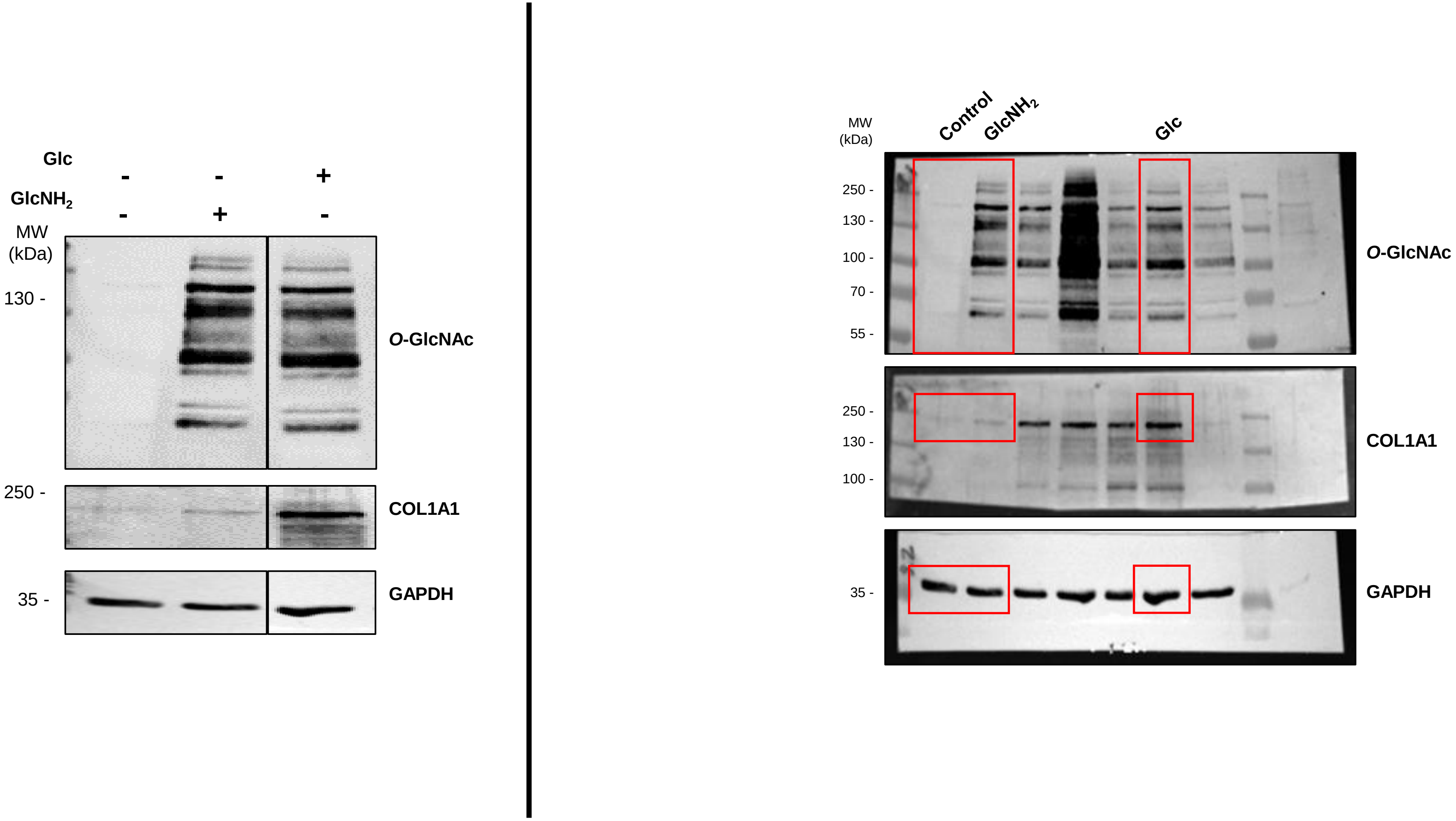

Supplementary Figure 2B

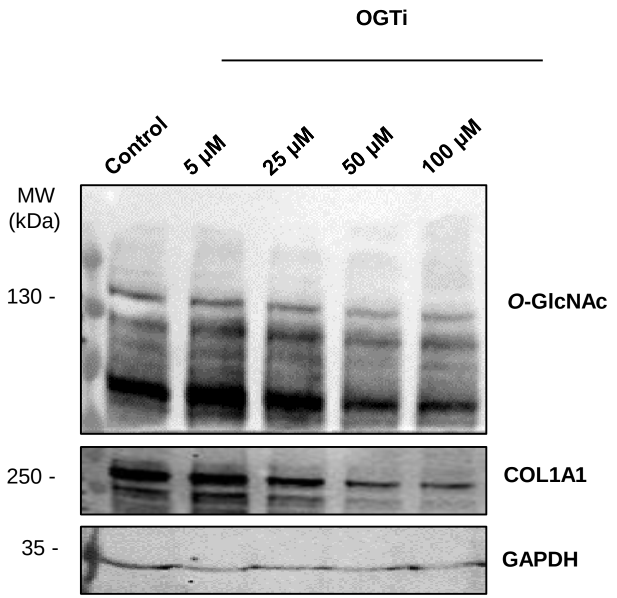

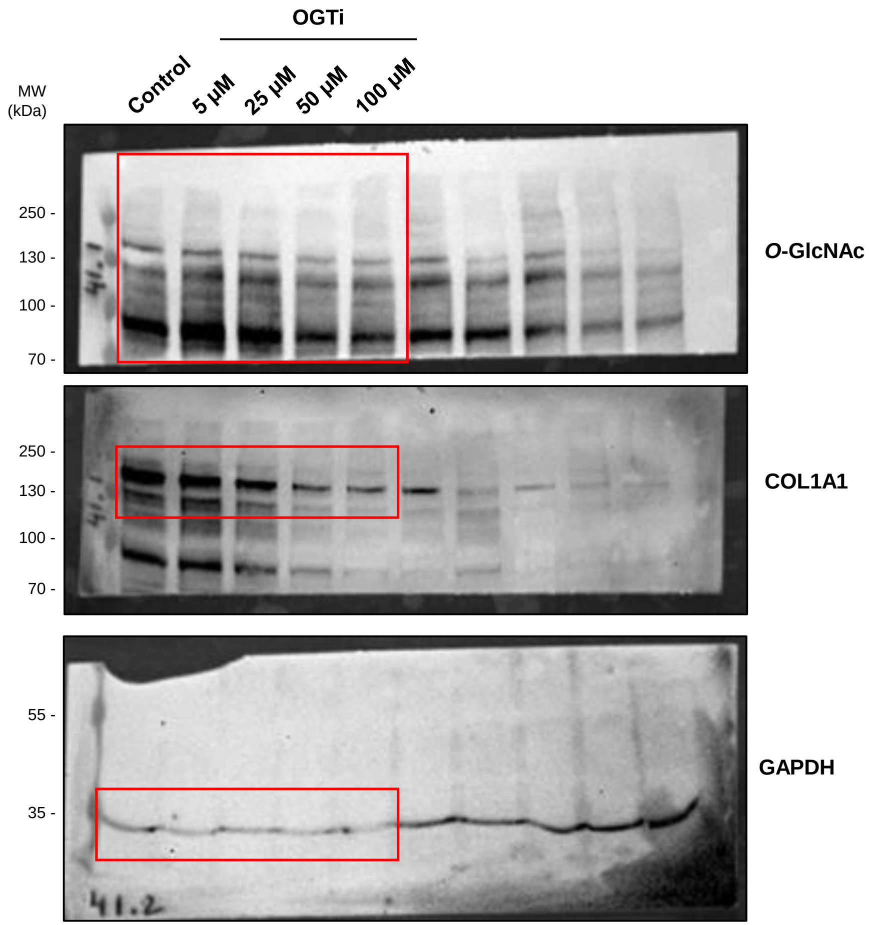

Supplementary Figure 2E

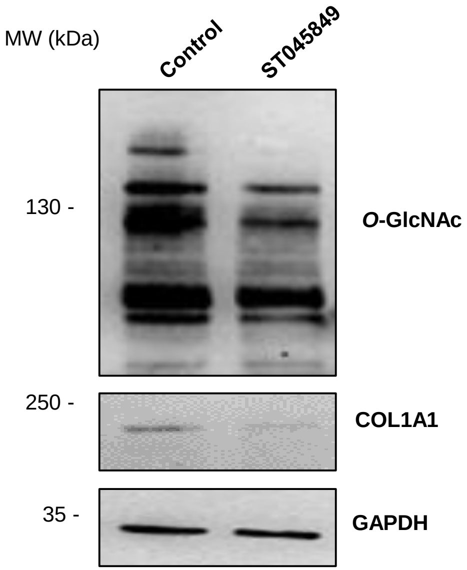

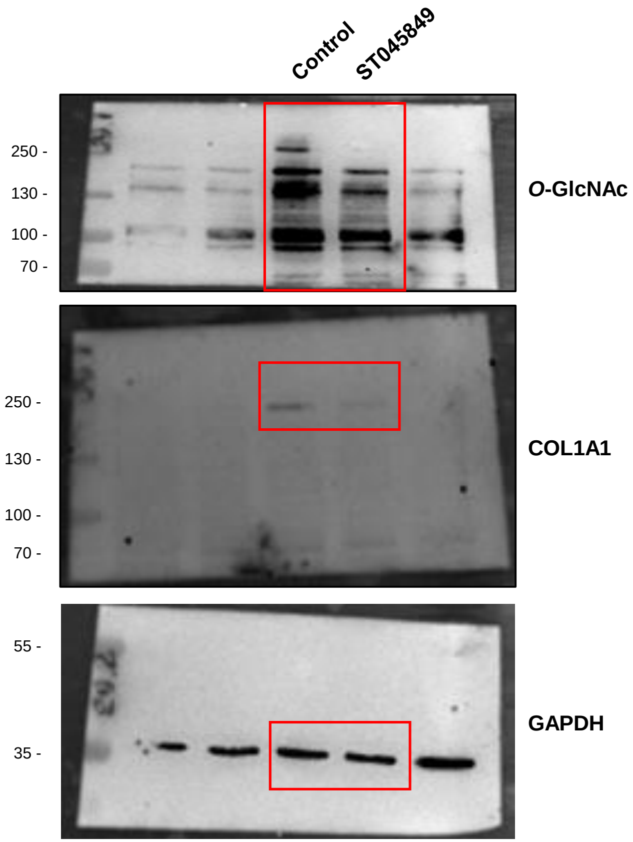

Figure 3C

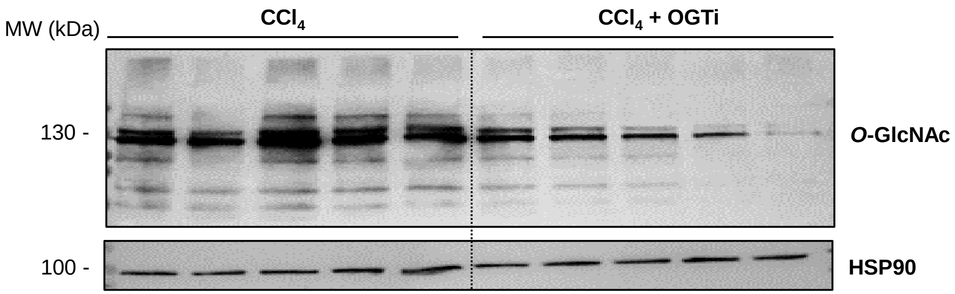
MW (kDa)
CCl4
CCl4 + OGTi
130 -
O-GlcNAc
100 -
HSP90

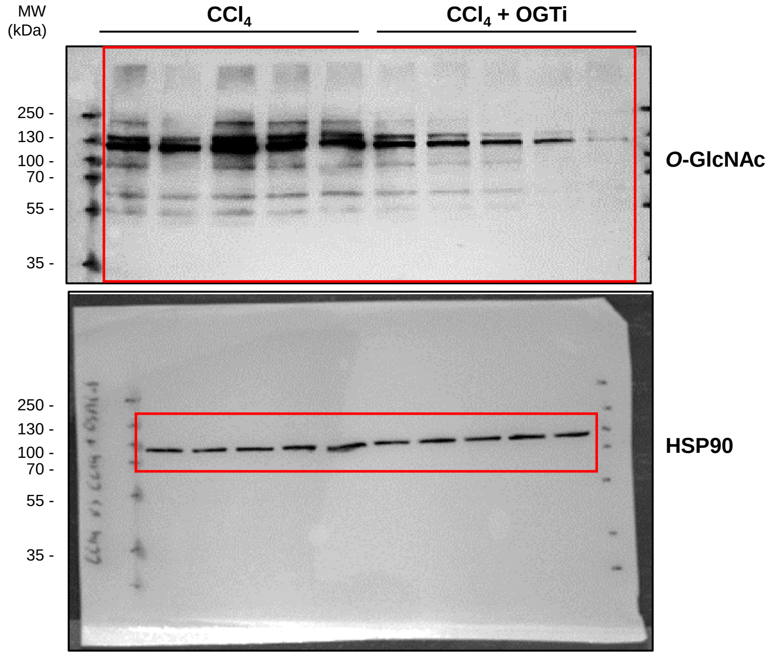
MW
(kDa)
CCl4
CCl4 + OGTi
250 -
130 -
100 -
70 -
55 -
35 -
O-GlcNAc
250 -
130 -
100 -
70 -
55 -
35 -
HSP90

Figure 6H

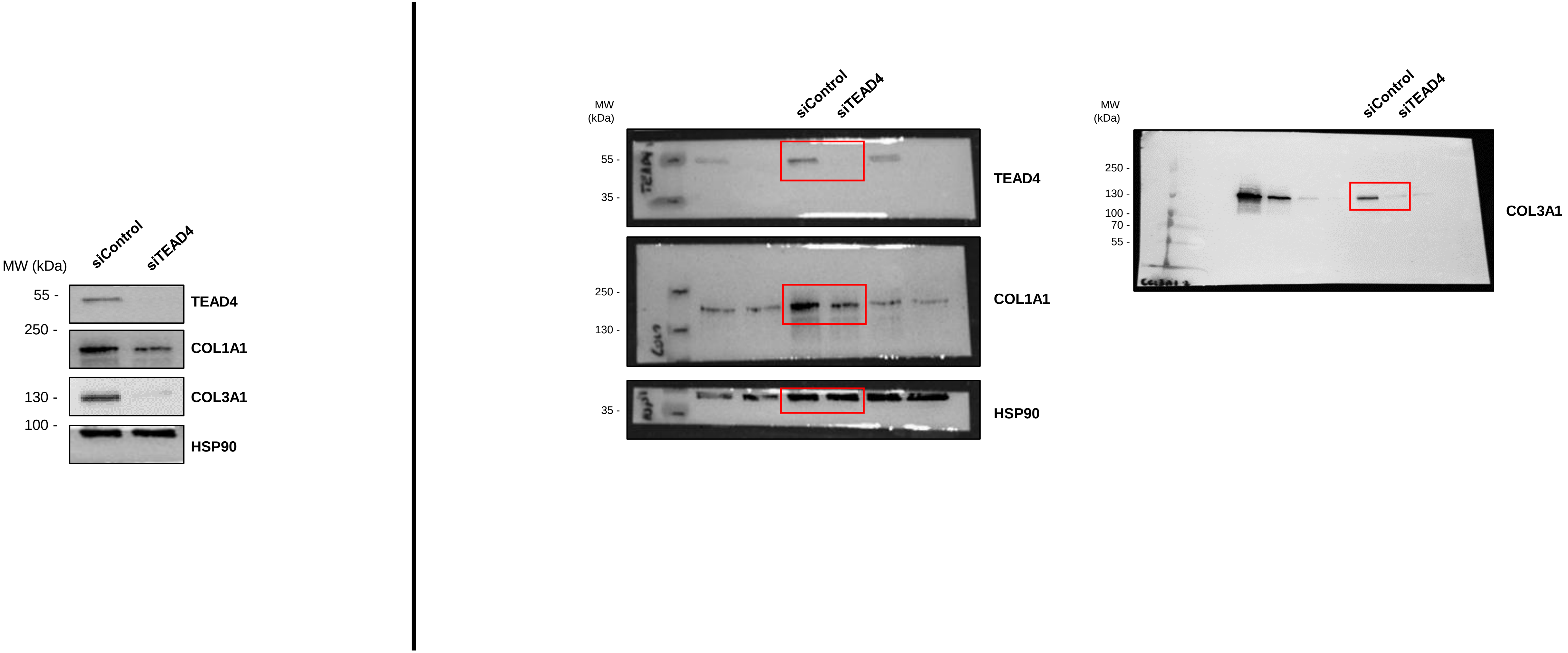

MW (kDa)
siControl
siTEAD4
55 -
TEAD4
250 -
COL1A1
130 -
COL3A1
100 -
HSP90
MW
(kDa)
siControl
siTEAD4
55 -
35 -
TEAD4
250 -
130 -
COL1A1
35 -
HSP90
MW
(kDa)
siControl
siTEAD4
250 -
130 -
100 -
70 -
55 -
COL3A1

Figure 6J

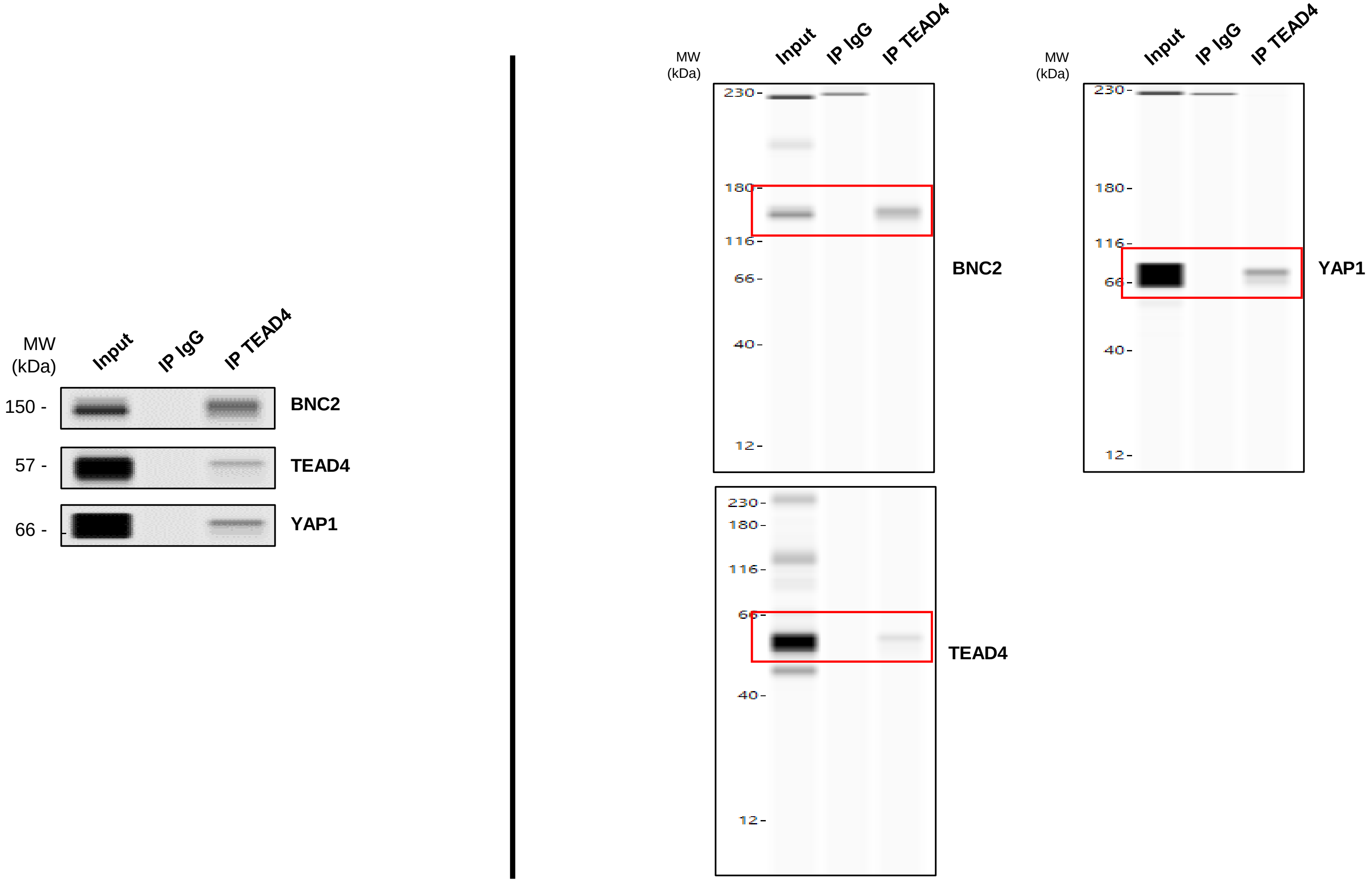
MW (kDa)
Input
IP IgG
IP TEAD4
150 -
BNC2
57 -
TEAD4
66 -
YAP1
230-
180-
116-
66-
40-
12-
BNC2
TEAD4
YAP1

Supplementary Figure 6F

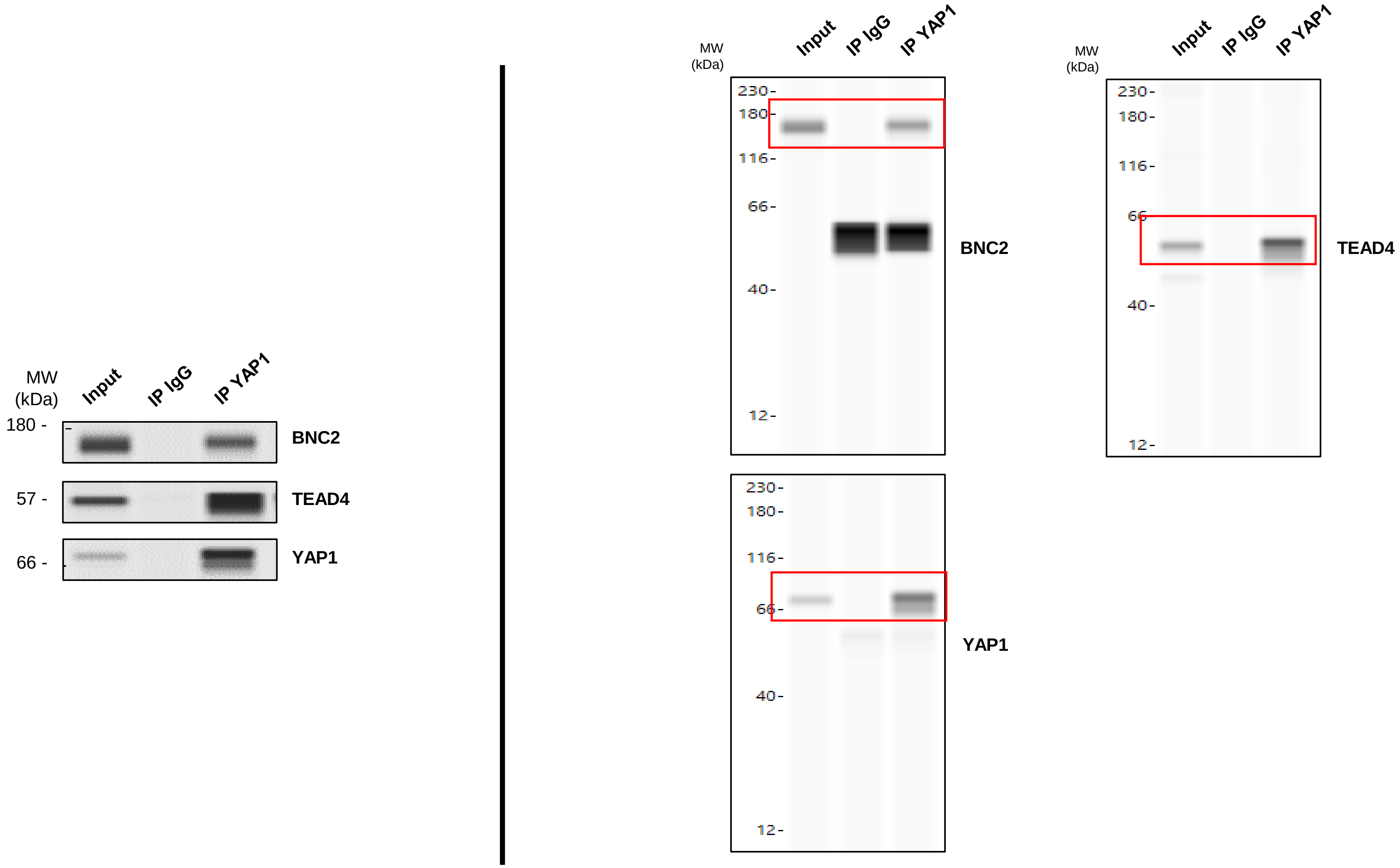

Supplementary Figure 7

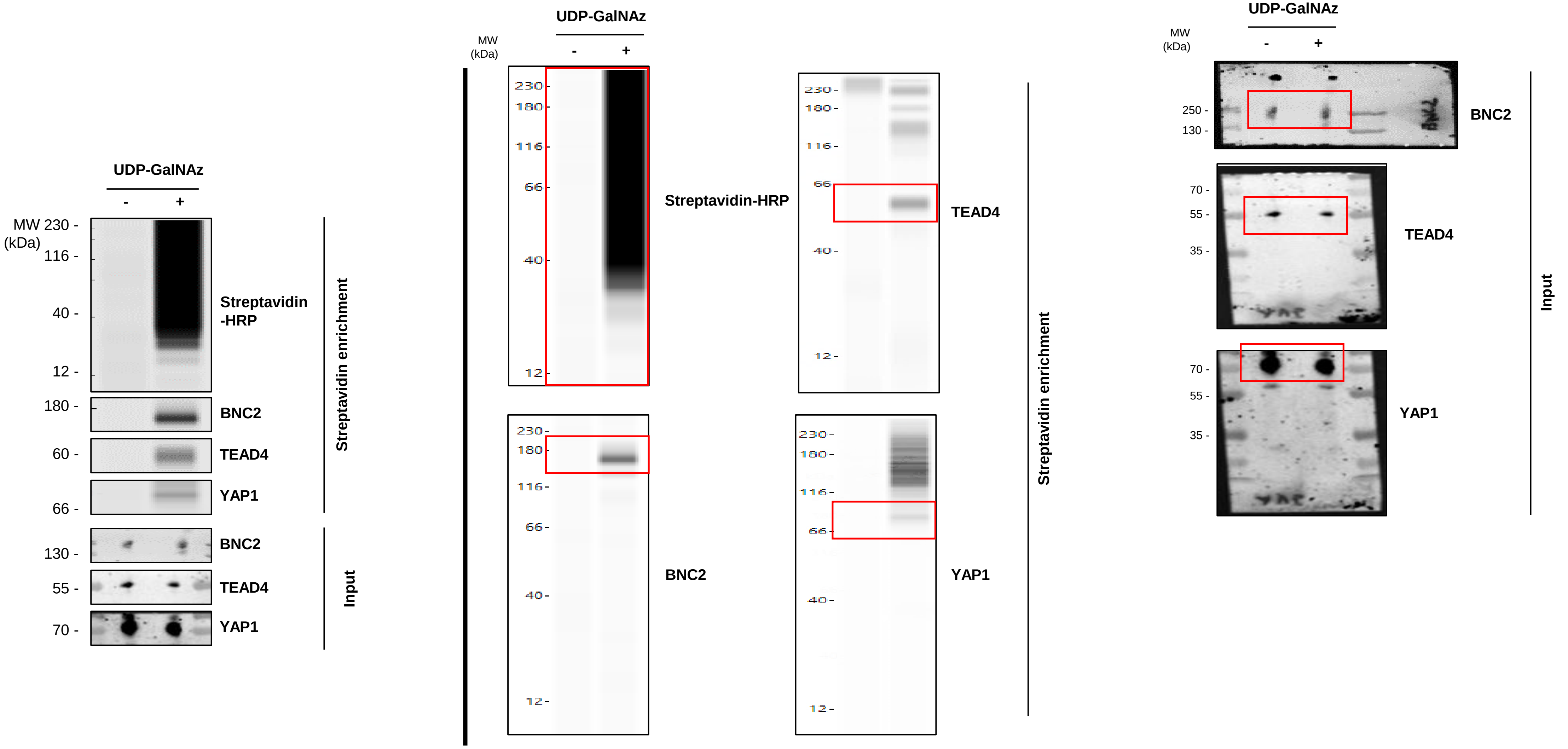

Figure 7A

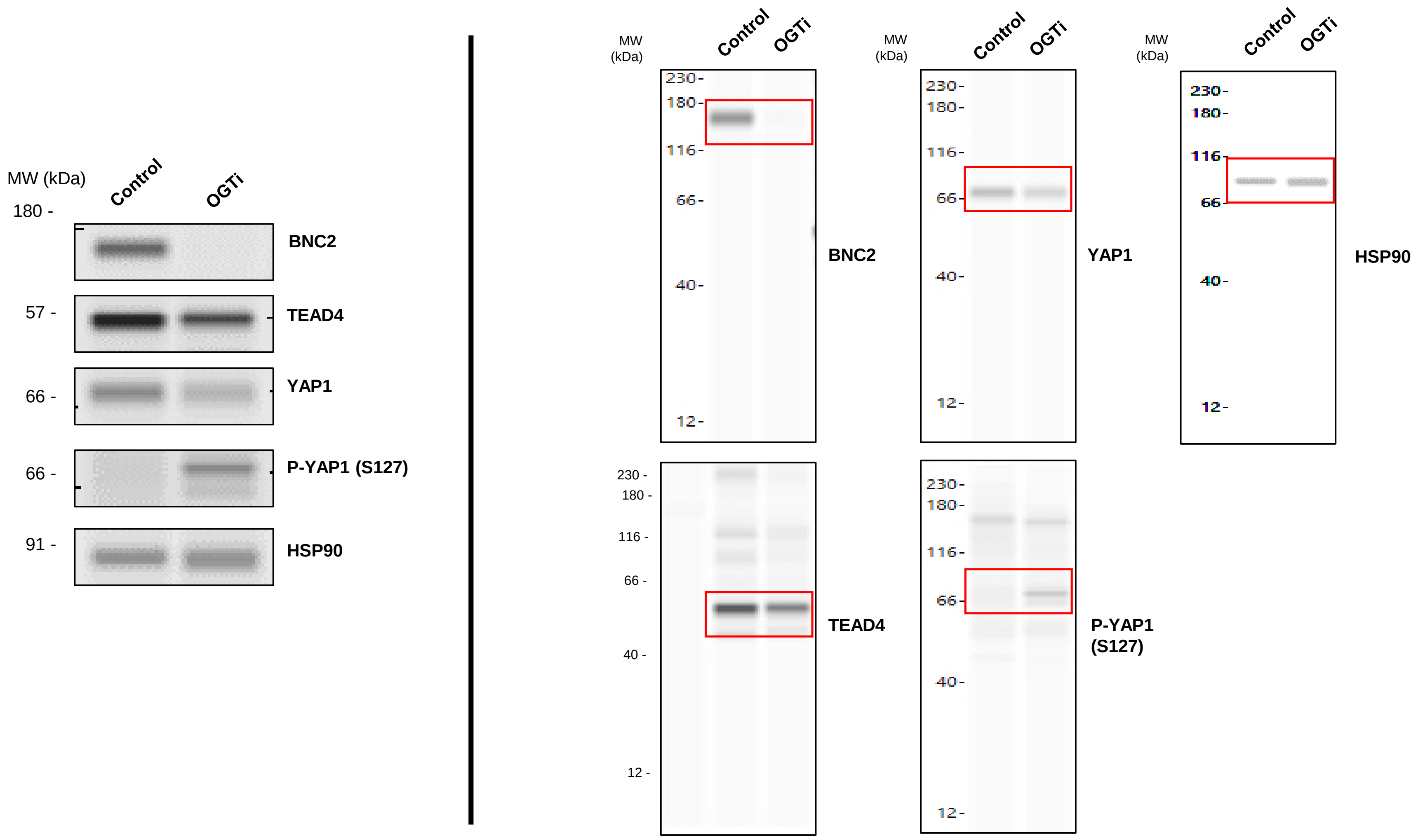
MW (kDa)
Control
OGTi
180 -
BNC2
57 -
TEAD4
66 -
YAP1
66 -
P-YAP1 (S127)
91 -
HSP90
MW
(kDa)
230-
180-
116-
66-
40-
12-
BNC2
YAP1
HSP90
230 -
180 -
116 -
66 -
40 -
12 -
TEAD4
P-YAP1
(S127)

Figure 7C

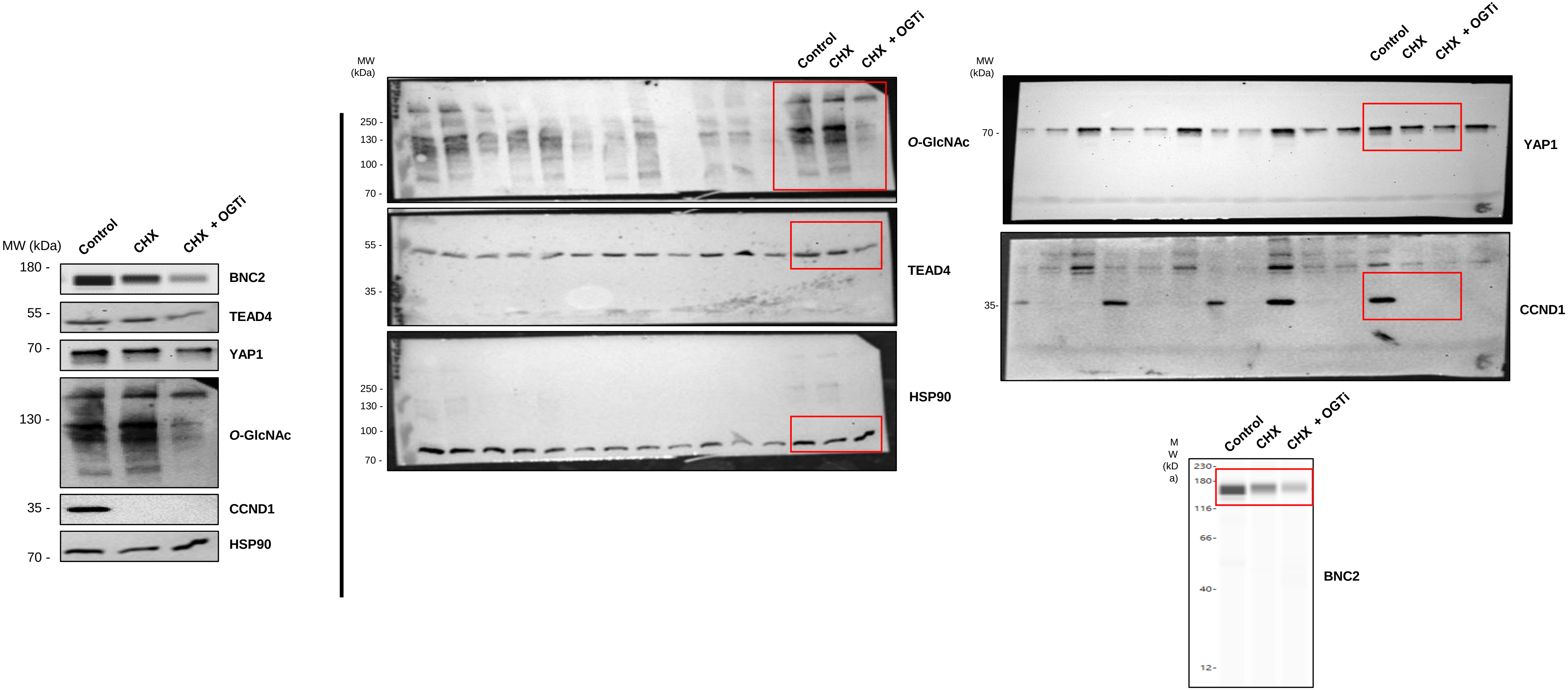
MW (kDa)
Control
CHX
CHX + OGTi
180 -
BNC2
55 -
TEAD4
70 -
YAP1
130 -
O-GlcNAc
35 -
CCND1
HSP90
70 -
MW (kDa)
250 -
130 -
100 -
70 -
O-GlcNAc
55 -
35 -
TEAD4
250 -
130 -
100 -
70 -
HSP90
70 -
YAP1
35-
CCND1
MW (kDa)
230-
180-
116-
66-
40-
12-
BNC2

Figure 7D

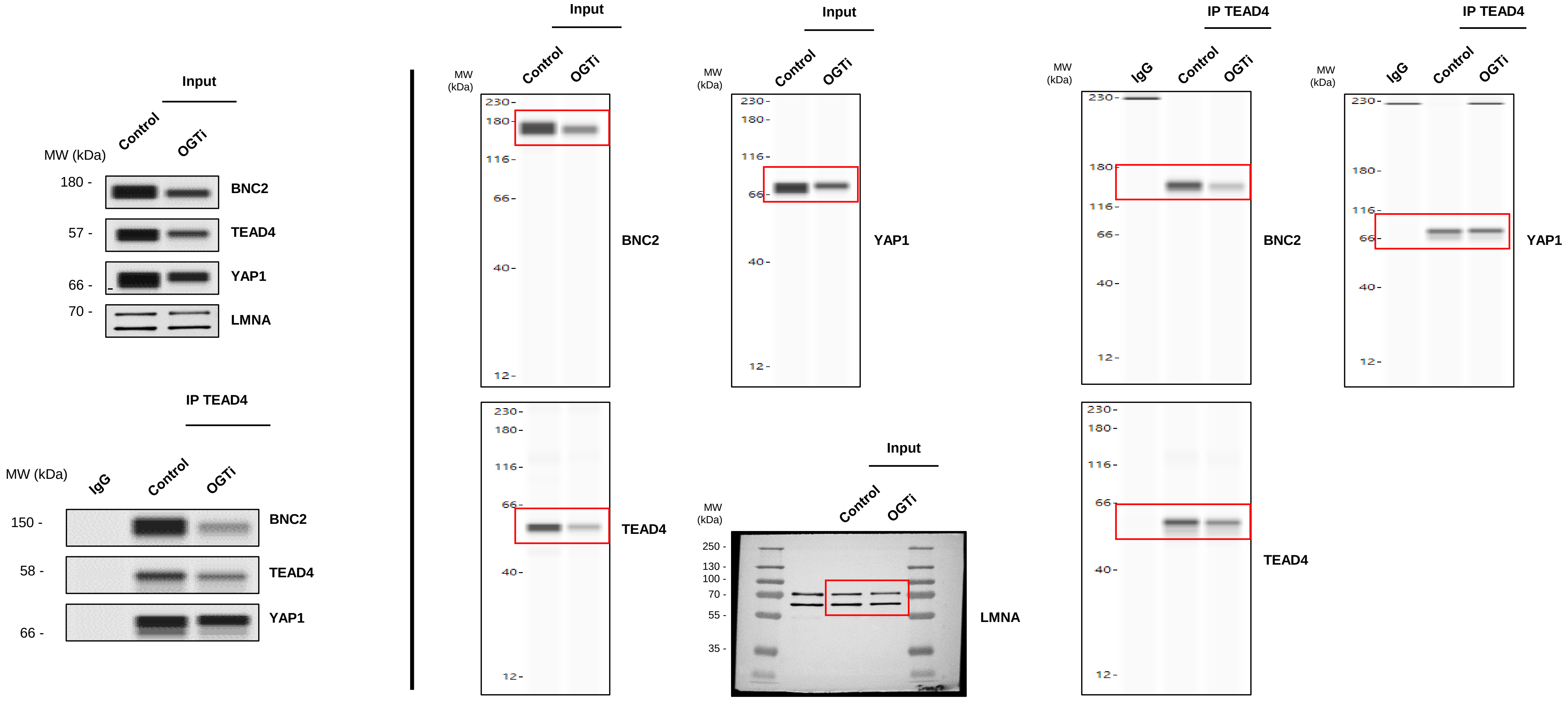
Input
MW (kDa)
Control
OGTi
180 -
BNC2
57 -
TEAD4
66 -
YAP1
70 -
LMNA
IP TEAD4
MW (kDa)
IgG
Control
OGTi
150 -
BNC2
58 -
TEAD4
66 -
YAP1
Input
MW (kDa)
230-
180-
116-
66-
40-
12-
BNC2
TEAD4
Input
YAP1
Input
250 -
130 -
100 -
70 -
55 -
35 -
LMNA
IP TEAD4
BNC2
TEAD4
IP TEAD4
YAP1

Figure 7E

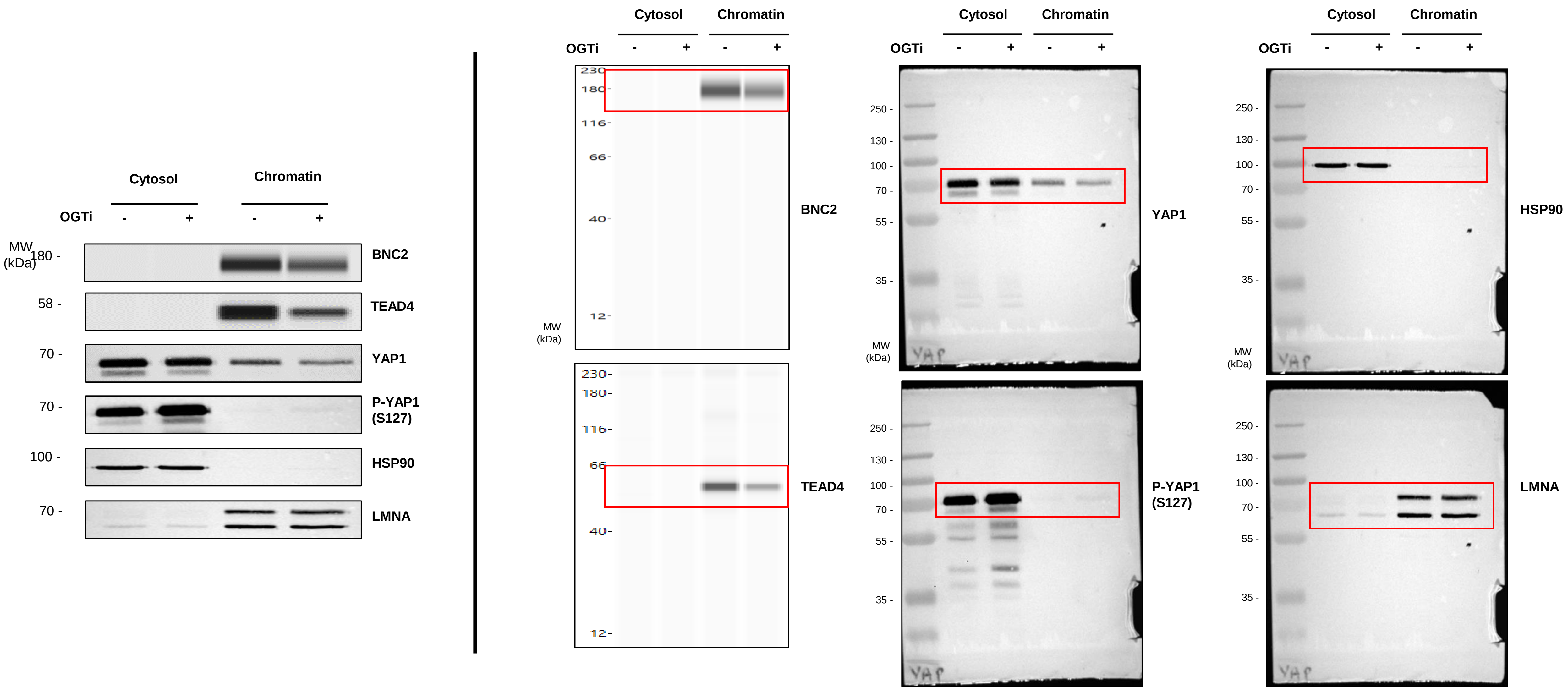

Cytosol
Chromatin
OGTi
-
+
-
+
MW
(kDa)
180 -
58 -
70 -
70 -
100 -
70 -
BNC2
TEAD4
YAP1
P-YAP1
(S127)
HSP90
LMNA
230
180
116
66
40
12
250 -
130 -
100 -
70 -
55 -
35 -

Figure 8B

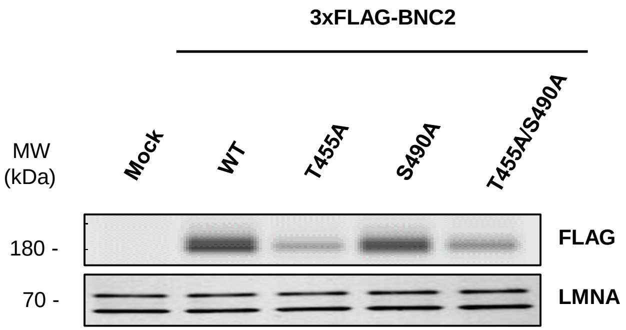
3xFLAG-BNC2
MW (kDa)
Mock
WT
T455A
S490A
T455A/S490A
180 -
70 -
FLAG
LMNA

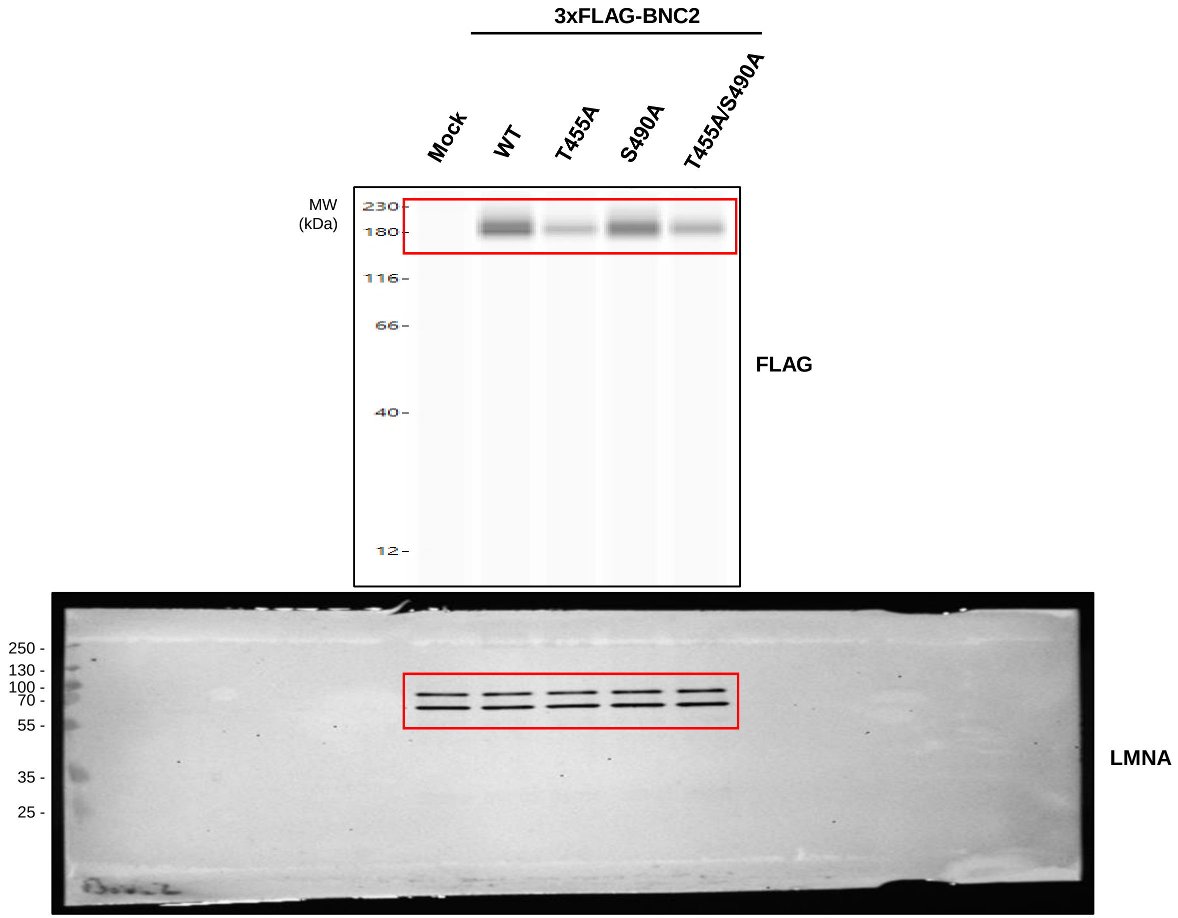
3xFLAG-BNC2
Mock
WT
T455A
S490A
T455A/S490A
MW (kDa)
230
180
116
66
40
12
FLAG
250 -
130 -
100 -
70 -
55 -
35 -
25 -
LMNA

Figure 8C

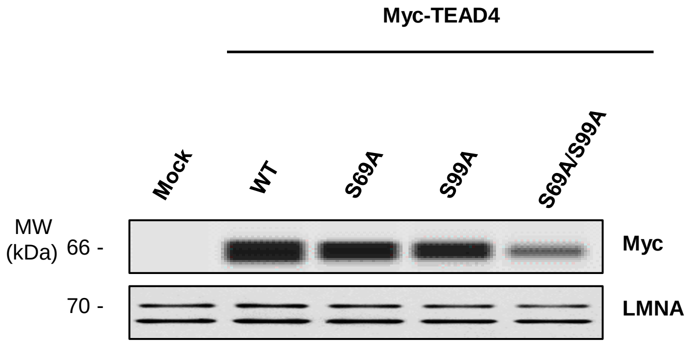
Myc-TEAD4
Mock
WT
S69A
S99A
S69A/S99A
MW (kDa)
66 -
70 -
Myc
LMNA

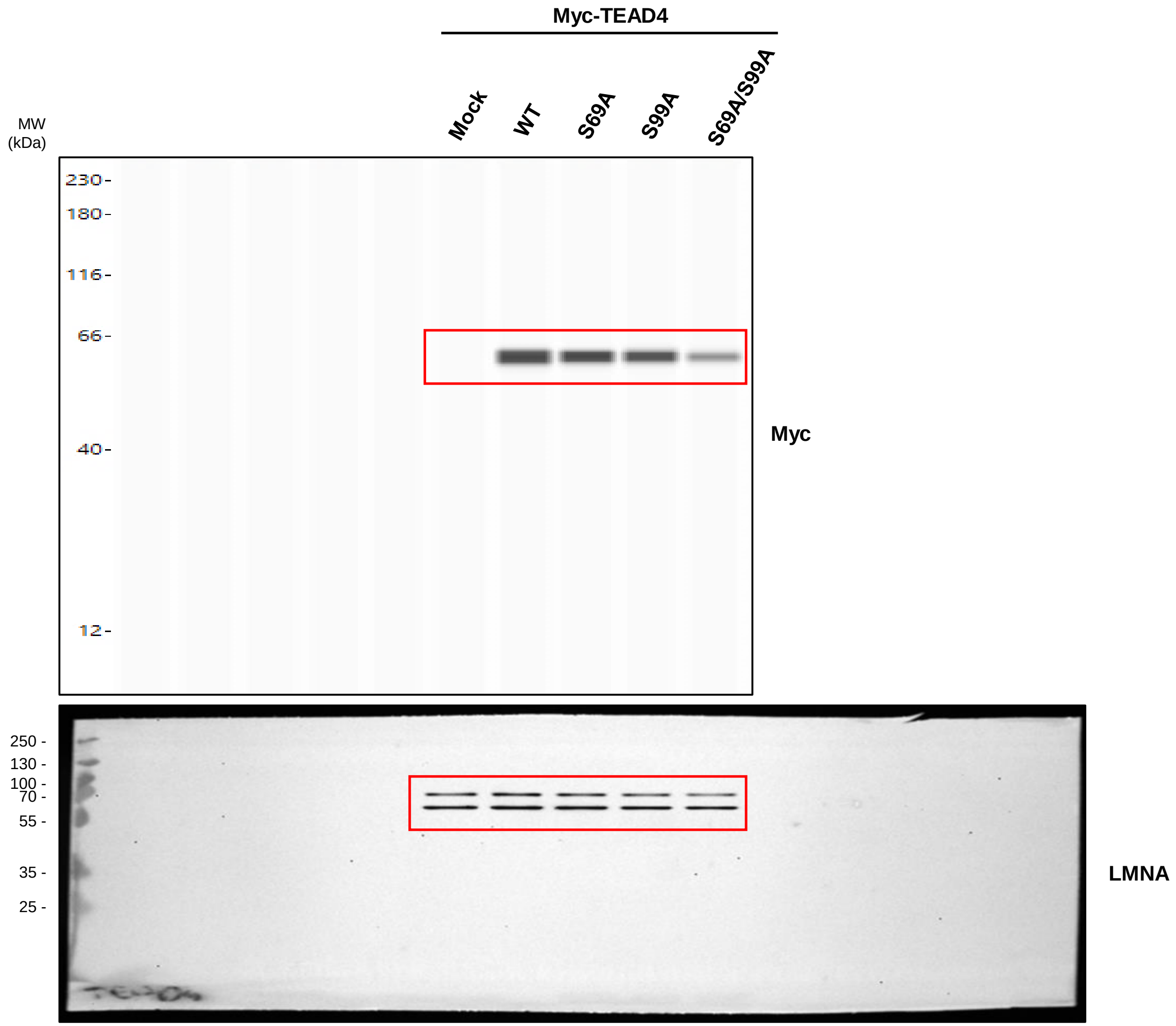
Myc-TEAD4
Mock
WT
S69A
S99A
S69A/S99A
MW (kDa)
230-
180-
116-
66-
40-
12-
Myc
250 -
130 -
100 -
70 -
55 -
35 -
25 -
LMNA

Figure 8D

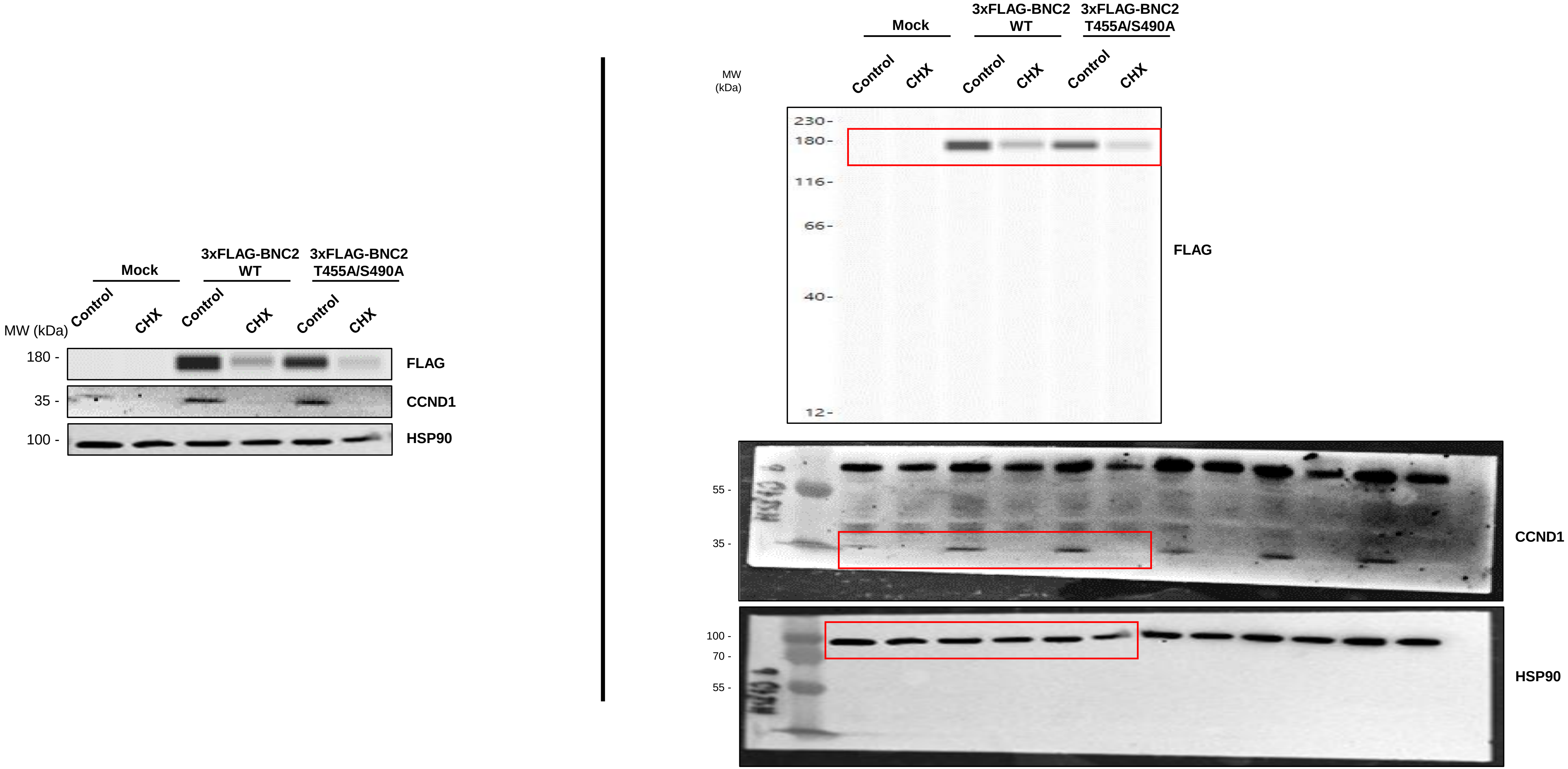

Mock
3xFLAG-BNC2 WT
3xFLAG-BNC2 T455A/S490A
Control
CHX
Control
CHX
Control
CHX
MW (kDa)
180 -
35 -
100 -
FLAG
CCND1
HSP90
MW (kDa)
230-
180-
116-
66-
40-
12-
55 -
35 -
100 -
70 -
55 -

Figure 8E

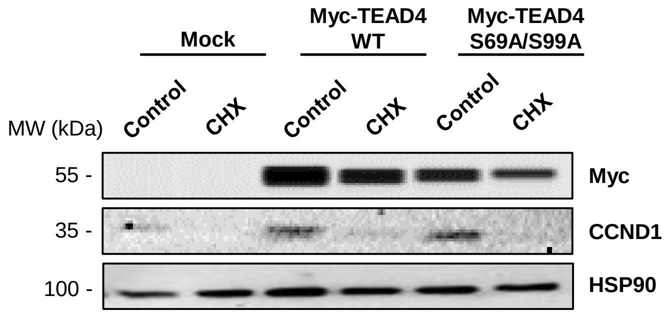

Mock
Myc-TEAD4 WT
Myc-TEAD4 S69A/S99A
Control
CHX
Control
CHX
Control
CHX
MW (kDa)
55 -
35 -
100 -
Myc
CCND1
HSP90

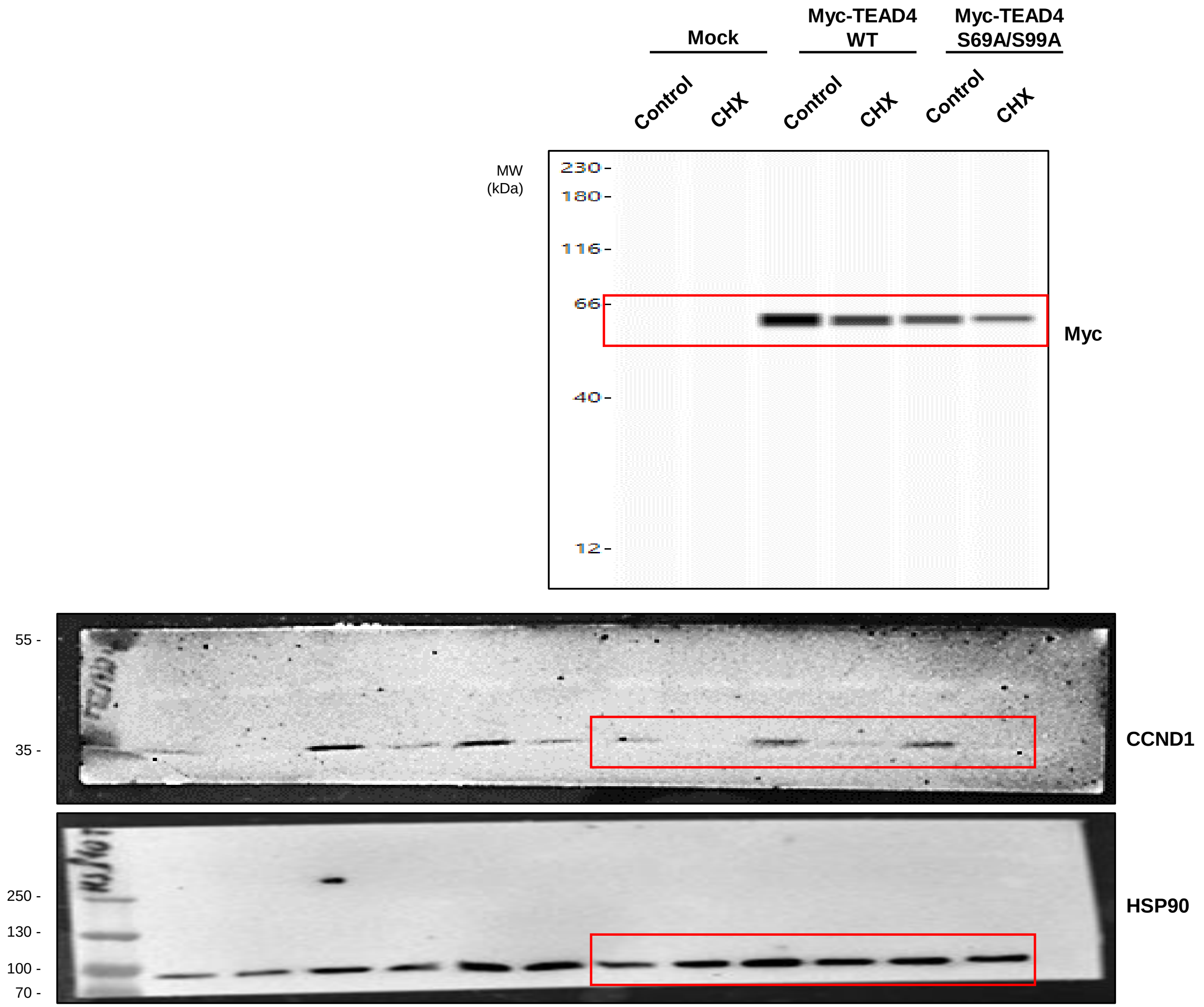

Mock
Myc-TEAD4 WT
Myc-TEAD4 S69A/S99A
Control
CHX
Control
CHX
Control
CHX
MW (kDa)
230 -
180 -
116 -
66 -
40 -
12 -
Myc
55 -
35 -
CCND1
250 -
130 -
100 -
70 -
HSP90

Supplementary Figure 8A

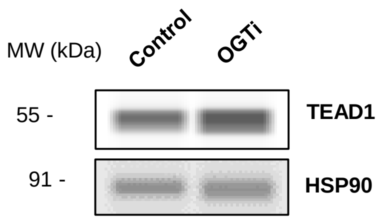

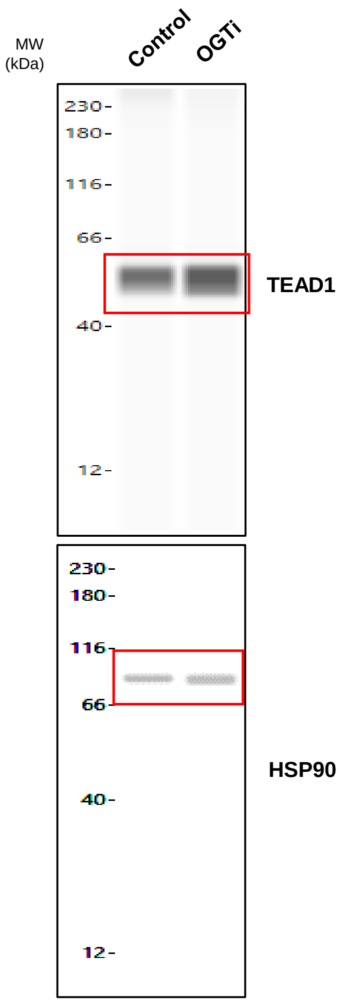

Supplementary Figure 8B

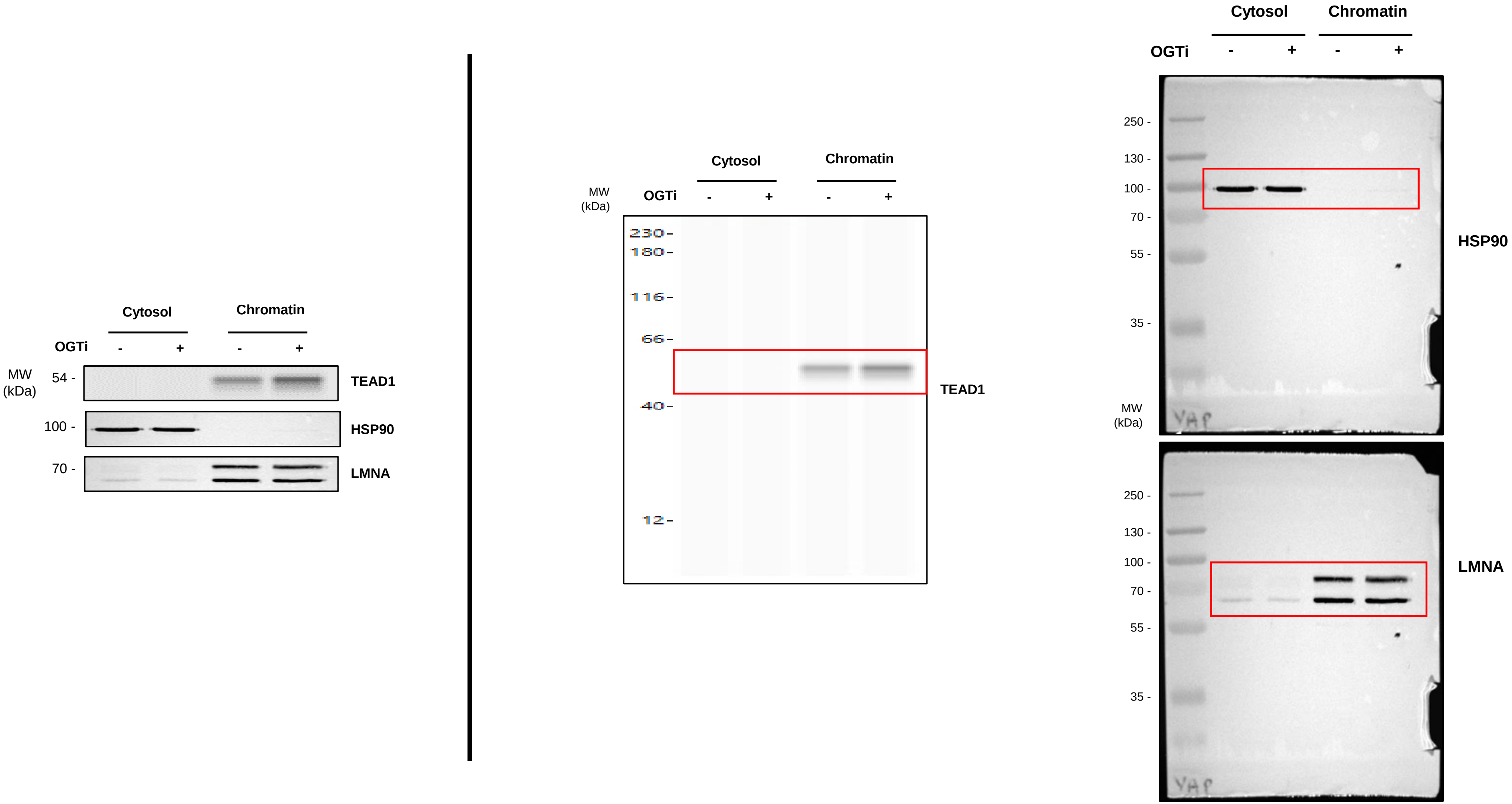

Figure 9A

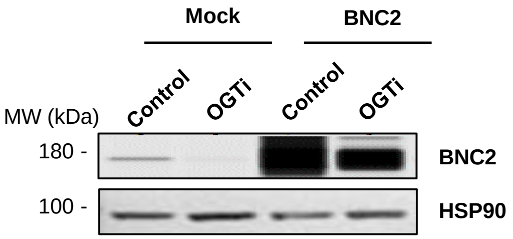
Mock
BNC2
Control
OGTi
Control
OGTi
MW (kDa)
180 -
100 -
BNC2
HSP90

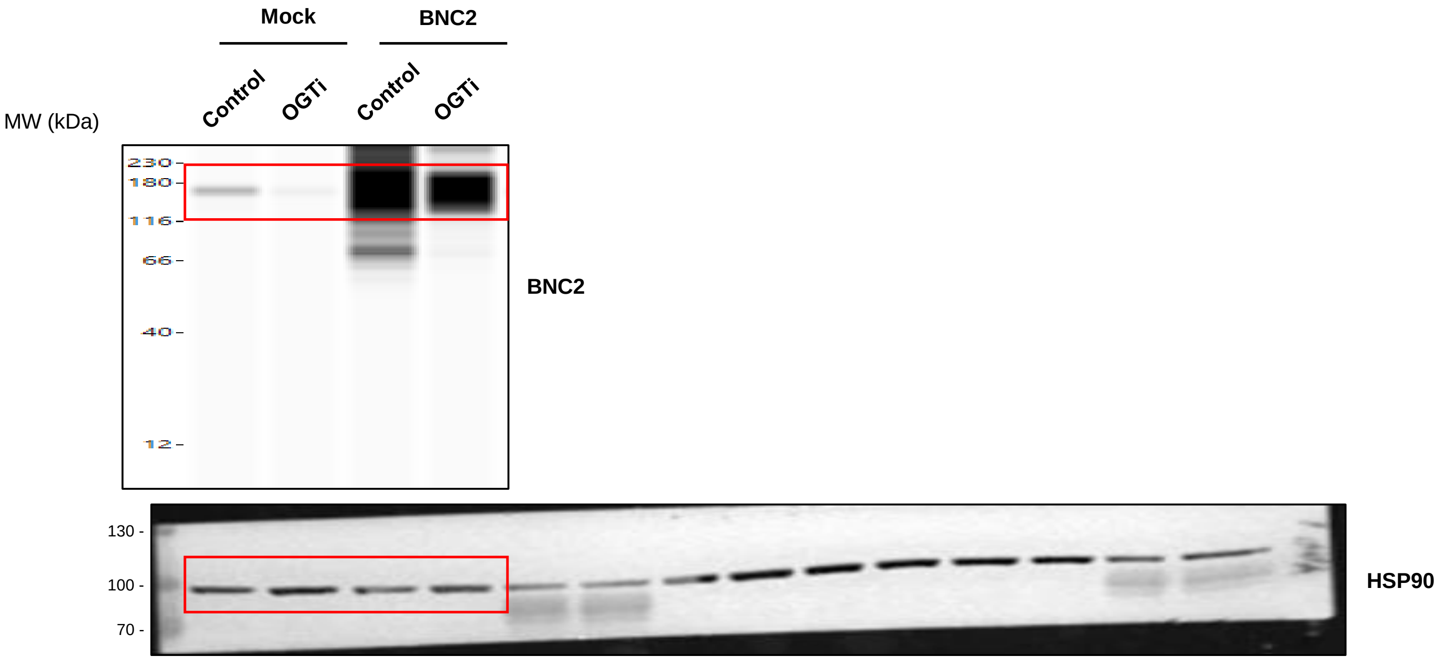
Mock
BNC2
Control
OGTi
Control
OGTi
MW (kDa)
230
180
116
66
40
12
BNC2
130 -
100 -
70 -
HSP90

Supplementary Figure 10

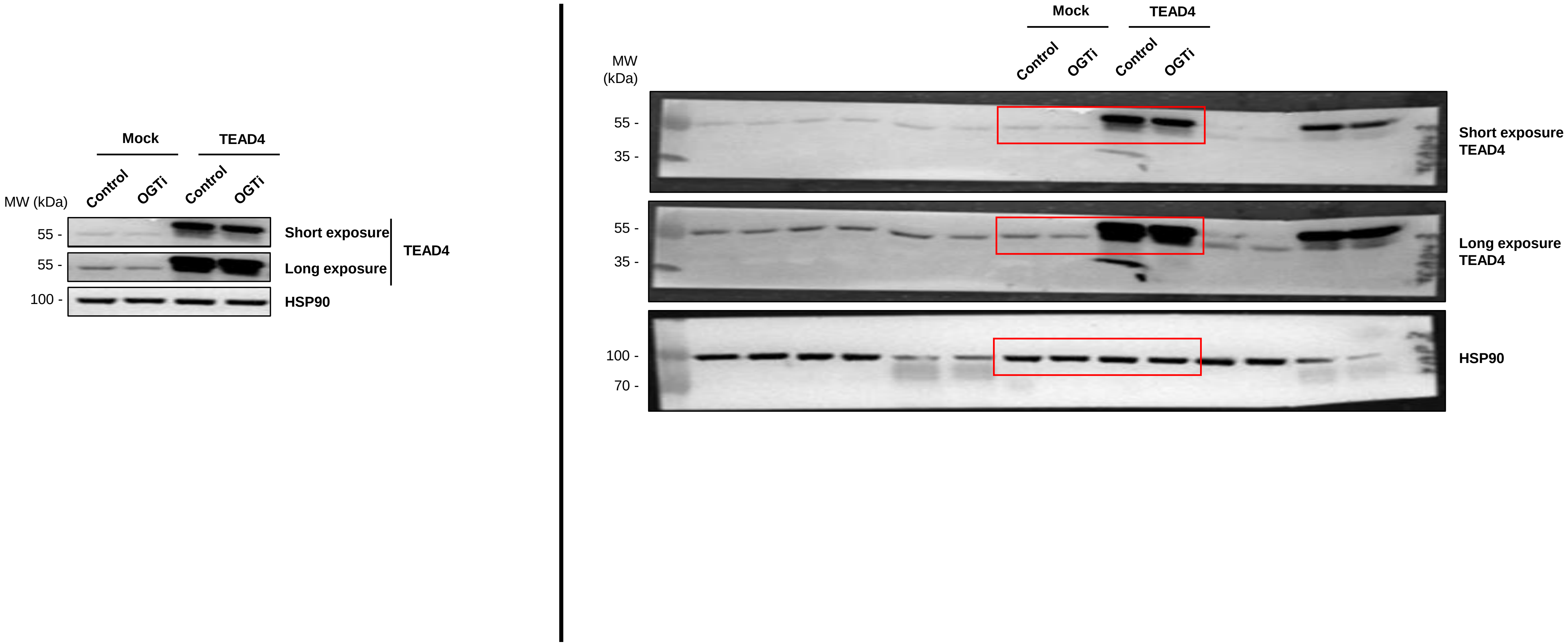

Supplementary Figure 11

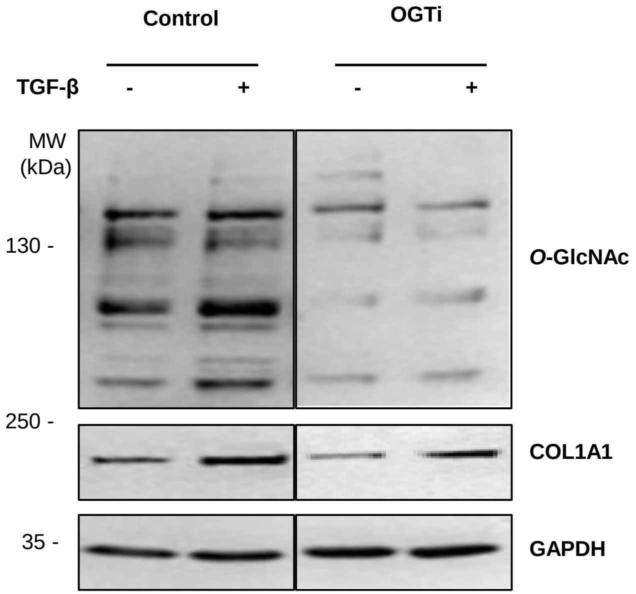

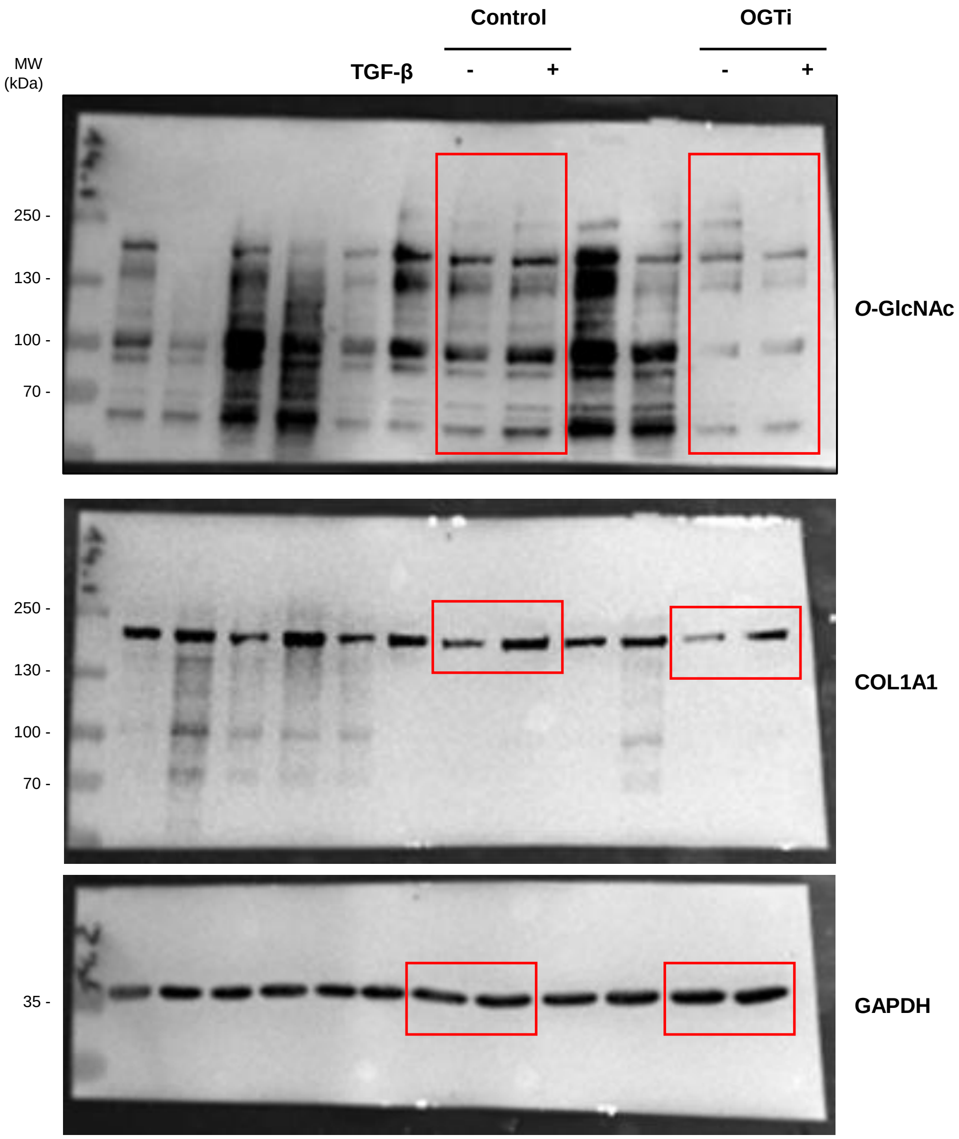

Supplement: Supplementary file 3 — Uncropped images [file 41419_2024_6773_MOESM3_ESM.pdf]
